# A chromosome-level assembly supports genome-wide investigation of the DMRT gene family in the golden mussel (*Limnoperna fortunei*)

**DOI:** 10.1093/gigascience/giad072

**Published:** 2023-09-30

**Authors:** João Gabriel R. N. Ferreira, Juliana A. Americo, Danielle L. A. S. do Amaral, Fábio Sendim, Yasmin R. da Cunha, Tree of Life Programme, Mark Blaxter, Marcela Uliano-Silva, Mauro de F. Rebelo

**Affiliations:** Bio Bureau Biotecnologia, Rio de Janeiro 21941-850, Brazil; Instituto de Biofísica Carlos Chagas Filho, Universidade Federal do Rio de Janeiro, Rio de Janeiro 21941-170, Brazil; Bio Bureau Biotecnologia, Rio de Janeiro 21941-850, Brazil; Bio Bureau Biotecnologia, Rio de Janeiro 21941-850, Brazil; Bio Bureau Biotecnologia, Rio de Janeiro 21941-850, Brazil; Instituto de Biofísica Carlos Chagas Filho, Universidade Federal do Rio de Janeiro, Rio de Janeiro 21941-170, Brazil; Bio Bureau Biotecnologia, Rio de Janeiro 21941-850, Brazil; Instituto de Biofísica Carlos Chagas Filho, Universidade Federal do Rio de Janeiro, Rio de Janeiro 21941-170, Brazil; Tree of Life, Wellcome Sanger Institute, Hinxton CB10 1RQ, UK; Tree of Life, Wellcome Sanger Institute, Hinxton CB10 1RQ, UK; Tree of Life, Wellcome Sanger Institute, Hinxton CB10 1RQ, UK; Instituto de Biofísica Carlos Chagas Filho, Universidade Federal do Rio de Janeiro, Rio de Janeiro 21941-170, Brazil

**Keywords:** golden mussel, *Limnoperna fortunei*, genome, invasive species, sex differentiation, DMRT

## Abstract

**Background:**

The golden mussel (*Limnoperna fortunei*) is a highly invasive species that causes environmental and socioeconomic losses in invaded areas. Reference genomes have proven to be a valuable resource for studying the biology of invasive species. While the current golden mussel genome has been useful for identifying new genes, its high fragmentation hinders some applications.

**Findings:**

In this study, we provide the first chromosome-level reference genome for the golden mussel. The genome was built using PacBio HiFi, 10X, and Hi-C sequencing data. The final assembly contains 99.4% of its total length assembled to the 15 chromosomes of the species and a scaffold N50 of 97.05 Mb. A total of 34,862 protein-coding genes were predicted, of which 84.7% were functionally annotated. A significant (6.48%) proportion of the genome was found to be in a hemizygous state. Using the new genome, we have performed a genome-wide characterization of the Doublesex and Mab-3 related transcription factor gene family, which has been proposed as a target for population control strategies in other species.

**Conclusions:**

From the applied research perspective, a higher-quality genome will support genome editing with the aim of developing biotechnology-based solutions to control invasion. From the basic research perspective, the new genome is a high-quality reference for molecular evolutionary studies of Mytilida and other Lophotrochozoa, and it may be used as a reference for future resequencing studies to assess genomic variation among different golden mussel populations, unveiling potential routes of dispersion and helping to establish better control policies.

## Data Description

### Context

*Limnoperna fortunei* (NCBI:txid356393)—popularly known as the golden mussel—is a freshwater bivalve species native to Southeast China that has successfully established itself as an invasive species in other Asian countries (Cambodia, Japan, Laos, South Korea, Taiwan, and Thailand) and in several South American countries (Argentina, Brazil, Paraguay, and Uruguay) [1]. Because of its impact on ecosystem structure and function, the golden mussel is considered an efficient ecosystem engineer, and its establishment is associated with changes in local biodiversity and nutrient recycling [2, 3]. Socioeconomic impacts are also relevant where golden mussel aggregates bind and obstruct net cages and hydroelectric power plant equipment [4, 5]. In the Brazilian hydroelectric sector alone, it is estimated that the golden mussel causes an annual $120 million loss due to longer and more frequent stops for maintenance [6]. Current control strategies have proven to be ineffective, and the species has continued to spread. Alternative biotechnological solutions have been proposed [6], and one possibility is to apply molecular tools to disrupt genes involved in reproductive behavior. This has been tested in other species, such as the malaria mosquito, where a disrupted genotype is rapidly spreading through the population using a gene drive system [7, 8].

The Doublesex and Mab-3 related transcription factor (DMRT) gene family is highly conserved in animals and contains members that play important roles in sexual differentiation. DMRT genes regulate gene expression through a conserved zinc finger DNA binding domain named DM. Most animals contain multiple DMRT genes, which act in developmental processes such as somitogenesis, neurogenesis, and gametogenesis [9–11]. The doublesex (Dsx) gene is present in insects and is required for both male and female sexual differentiation according to the sex-specific isoform that is produced after alternative splicing [12–14]. In nematodes, *mab-3* (male abnormal 3) acts as a critical factor for male sex determination [15, 16]. In vertebrates, DMRT1 is required for masculinization of somatic cells [17, 18]. In mollusks, it is assumed that DMRT1-like genes are involved in male sex differentiation, given the male-biased expression pattern in the gonads shared by many different species [19–21]. DMRT is an attractive candidate to disrupt golden mussel reproduction.

Reference genomes are an important resource for the study of invasive species. They have been used to study invasion dynamics, identifying molecular mechanisms conferring adaptiveness as well as promising genes for biotechnology-based control strategies [22]. The current genome assembly for the golden mussel [23] is a highly fragmented representation of the 15 chromosomes (2n = 30) of the species [24, 25] assembled mostly from Illumina sequencing reads. Limitations of this draft genome constrain its applications in resequencing and comparative genomic studies and may lead to incomplete or erroneous gene models, as indicated by the high number of missing (10%) and fragmented (7%) BUSCO genes reported in the original study [23].

Recent advances in library preparation protocols, sequencing technologies, and bioinformatics algorithms have made the development of high-quality reference genomes scalable and affordable. In this study, we present a high-quality genome for the golden mussel where we have identified a widespread occurrence of hemizygosity over the chromosomes. We identified 4 DMRT genes in the golden mussel genome, which have been compared to DMRT genes from other bivalve species to study the evolution of this gene family in the class. One golden mussel DMRT is a putative sex differentiation gene showing male-biased expression in the gonads; therefore, it is a potential target for biotechnology-based control strategies. The new golden mussel genome is expected to be a valuable reference for future studies on the species.

### Sample collection

Golden mussel specimens were collected from the Taquari River, São Paulo, Brazil (23°16′′45.7′′S 49°12′′01.7′′W), on 17 March 2021. Three representative specimens were deposited in the molluscan collection of the National Museum administered by the Federal University of Rio de Janeiro (identification numbers: IB UFRJ 19950, IB UFRJ 19952, and IB UFRJ 19954). Other specimens were taxonomically identified by Dr. Igor Christo Miyahira. Finally, a set of specimens had their tissues—gonads, adductor muscle, digestive gland, gills, and foot—dissected and preserved in dry ice at −80ºC until and during transportation to the Wellcome Sanger Institute (WSI) in Hinxton, Cambridgeshire, United Kingdom, for further processing and sequencing.

### DNA extraction

DNA extraction was performed at the WSI's Tree of Life laboratory. Golden mussel samples were weighed and disrupted using a Covaris cryoPREP Automated Dry Pulveriser that subjects tissue—gill tissue was selected—to multiple impacts until it becomes a fine powder. In total, 25 mg of this powder was used for DNA extraction and 50 mg was set aside for Hi-C. DNA extraction was performed using a Qiagen MagAttract HMW DNA extraction kit on a KingFisher APEX liquid-handling system. Then, 50 ng DNA was submitted to 10X genomic sequencing with any low-molecular-weight DNA removed prior to sequencing using a 0.8× AMpure XP purification kit. Similarly, prior to submission to PacBio sequencing, high-molecular-weight DNA was sheared to an average fragment size of between 12 and 20 kb using a MegaRuptor 3 (speed setting 30). The sheared DNA was purified by solid-phase reversible immobilization using AMpure PB beads with a 1.8× ratio of beads to sample. The concentration of sheared DNA was assessed using a Qubit Fluorometer with Qubit dsDNA High Sensitivity Assay kit and Nanodrop spectrophotometer, while the fragment size distribution was assessed using an Agilent FemtoPulse.

### Sequencing

All sequencing libraries were constructed using DNA extracted from a single specimen, a female golden mussel with the unique Tree of Life identifier xbLimFort5. PacBio HiFi circular consensus and Chromium 10X Genomics linked-read sequencing libraries were constructed according to the manufacturers’ instructions. Sequencing was performed by the Scientific Operations core at WSI on PacBio SEQUEL II (HiFi) and Illumina NovaSeq (10X) instruments. Hi-C data were generated using the Arima v2.0 kit and sequenced on a NovaSeq 6000 instrument (RRID:SCR_016387).

### Overall genome characteristics

A *k*-mer–based approach was used to estimate overall genome statistics from the PacBio HiFi data. Jellyfish [26] was used to calculate the frequencies of 31-bp-long *k*-mers and GenomeScope (RRID:SCR_017014) [27] was used to build a model to infer genome characteristics. The genome was inferred to be diploid, with an estimated haploid size around 1.3 Gb (Supplementary Fig. S1). The expected repeat content was 43% and a high heterozygosity rate was estimated (2.4%).

### Genome assembly

**Figure 1:**
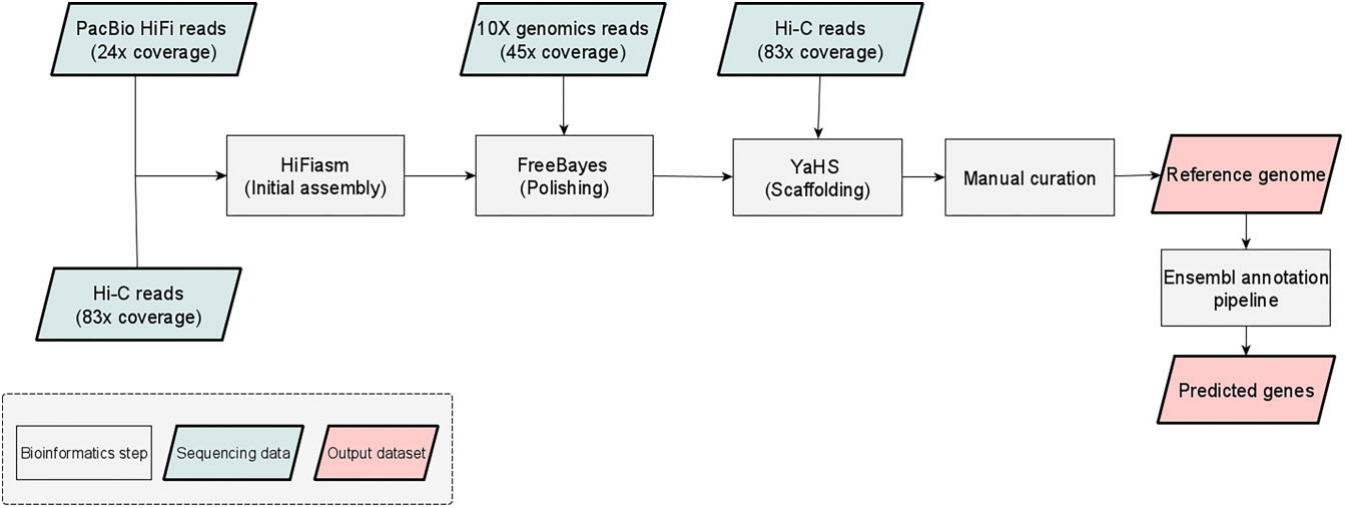
Genome assembly pipeline.

The genome assembly pipeline is summarized in Fig. 1. The initial set of contigs was assembled using HiFiasm (RRID:SCR_021069) v0.16.1 combining HiFi and Hi-C reads in the Hi-C integrated mode [28]. The 10X linked reads were mapped to contigs using LongRanger v2.2.2 [29], and then Freebayes (RRID:SCR_010761) v1.3.1 [30] was used to polish the contigs based on the 10X mapping. The polished contigs were then scaffolded using the YaHS pipeline v1.0 [31]. Finally, scaffolds were manually curated by WSI's Genome Reference Informatics Team (GRIT) following the protocol described by Howe et al. [32]. The curated scaffolds represent the final genome assembly, which was then annotated using the Ensembl Rapid Annotation Pipeline [33]. The mitochondrial genome was assembled using the MitoHiFi pipeline [34].

**Figure 2:**
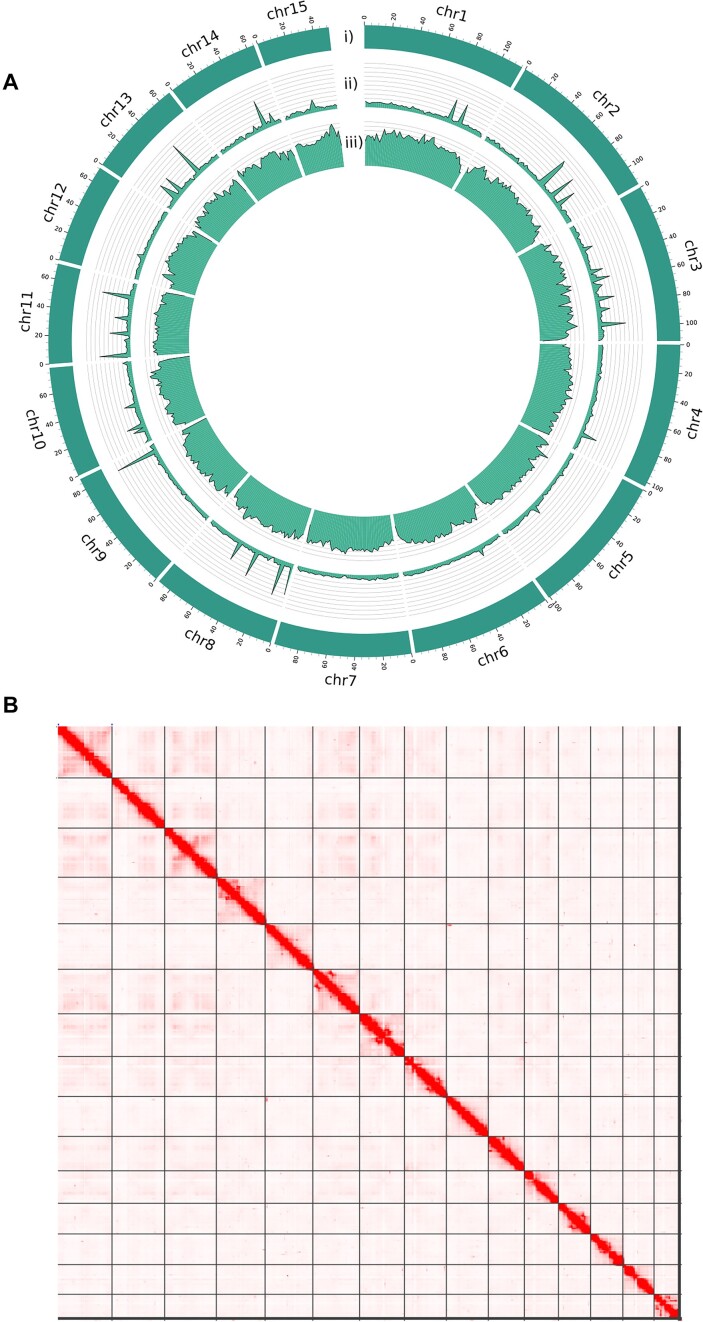
The genome landscape. (A) Circos representation of the 15 chromosomes assembled in this study. Each track represents (i) the size of each chromosome, (ii) the gene density, and the (iii) repeat density over the chromosome sequences, calculated using a 2-Mb window size. (B) Hi-C contact map with chromosomes displayed in size order from top to bottom and from left to right.

The size of the final genome assembly is 1.34 Gb (Supplementary Table S1). Most (99.24%) of its total length is distributed over the 15 largest scaffolds (Fig. 2), which correspond to the haploid chromosome number (*n* = 15) of the species [24]. The largest contig and the largest scaffold are 8.3 Mb and 115 Mb long, respectively, and the genome GC content is 33.6%. BUSCO (RRID:SCR_015008) v5.0 [33] completeness was 95.6% (with the metazoa_odb10 dataset). We used Merqury (RRID:SCR_022964) [34] with the PacBio HiFi reads and calculated an assembly-contained *k*-mer completeness of 99.23% and a quality value (QV) of 53, representing a base accuracy of 99.999% (Merqury plots can be found in Supplementary Fig. S2). All the quality metrics calculated for the new assembly conform to the standards of the Vertebrate Genomes Project (VGP) for what is considered a high-quality genome [35].

Table 1 presents genomic statistics of the previous draft assembly and the chromosome-level reference produced in this study. The chromosome-level reference scaffold N50 is 313-fold greater than its predecessor draft genome. An improvement has also been achieved in genome completeness, as shown by an increase in both *k*-mer–based completeness assessment and the percentage of complete BUSCO genes found.

**Table 1:** Comparison of assembly metrics between the draft and the new golden mussel genome

	Draft genome (GCA_003130415.1)	Chromosome-level genome (GCA_944474755.1)
Total assembly length (Gb)	1.67	1.34
GC content (%)	33.6	33.8
Number of scaffolds	20,580	309
Scaffold N50 (Mb)	0.31	97.05
Scaffold L50	1,489	7
Number of contigs	61,175	1,838
Contig N50 (Mb)	0.03	1.50
Contig L50	16,521	277
QV	14.89	53.36
Completeness (%)	47.83	68.55 (primary assembly) 99.23 (primary + alternate haplotype*)
BUSCO (metazoa_odb10)	C: 66.8% [S: 65.0%, D: 1.8%], F: 19.3%, M: 13.9%, *n* = 954	C: 95.6% [S: 95.0%, D: 0.6%], F: 2.2%, M: 2.2%, *n* = 954

BUSCO statistics. C, complete; D, complete and duplicated; F, fragmented; M, missing; S, complete and single-copy. *n* = number of BUSCO genes from reference dataset.

* Primary assembly: GCA_944474755.1; alternate haplotype: GCA_944589985.1.

### Repeat annotation

Detection and classification of repeat elements was done using the Earl Grey pipeline v1.3 [36]. Earl Grey was run with the RepeatMasker (RRID:SCR_012954) search term (-r) set to “mollusca.” Almost half (46.93%) of the genome was annotated as repetitive sequences, with 35.80% of the genome labeled as unclassified repeats (Table 2). Similarly, high proportions of unclassified repeats have been reported in other mussels [37, 38]. The second most frequent repeat class detected was long interspersed nuclear elements (LINEs), representing 4.51% of the total genome.

**Table 2:** Repetitive elements identified in the golden mussel genome

Classification*	Total sequence length (bp)	Sequences count	Proportion of genome (%)	Number of distinct classifications
DNA	46,269,487	64,938	3.46	201
LINE	60,245,208	64,949	4.51	224
LTR	28,342,781	50,395	2.12	112
Other (simple repeat, microsatellite, RNA)	216,907	244	0.02	2
Penelope	11,115,342	24,065	0.83	23
Rolling Circle	1,640,436	1,503	0.12	6
SINE	831,095	723	0.06	3
Unclassified	478,115,783	883,466	35.80	1,494

LINE, long interspersed nuclear element; LTR, long terminal repeat; SINE, short interspersed nuclear element.

* Classification in alphabetical order.

### Gene prediction

The Ensembl rapid annotation pipeline [33] was used to predict genes (Tables 3 and 4). The prediction was supported by homologous proteins and preexisting golden mussel RNA sequencing (RNA-seq) data, including RNA-seq data from the draft genome project and from the same specimen (xbLimFort5) sequenced for the chromosome-level genome assembly (Supplementary Table S2). A total of 34,862 protein-coding genes were predicted, with 68,899 proteins inferred. Most (53.5%) genes were associated with a single protein, with about 21.8% associated with 2 proteins and 24.7% with 3 or more proteins (Supplementary Table S3). In addition to the protein-coding genes, 58,911 noncoding genes were predicted, most of which (56.5%) were classified as long noncoding RNA (lncRNA) (Table 3).

**Table 3:** Categories of predicted genes

Statistics	Value
Protein-coding genes	34,862
Noncoding genes	58,911
lncRNA	33,258
Y_RNA	9,316
tRNA	7,582
Ribozyme	5,091
misc_RNA	1,641
rRNA	1,410
snRNA	565
snoRNA	47
scaRNA	1

lncRNA, long noncoding RNA; misc_RNA, miscellaneous RNA; rRNA, ribosomal RNA; scaRNA, small Cajal body-specific RNA; snRNA, small nuclear RNA; snoRNA, small nucleolar RNA; tRNA, transfer RNA.

**Table 4:** Gene prediction statistics

Statistics	Value
Average gene length (bp)	9,426
Protein-coding genes (bp)	17,765
Noncoding genes (bp)	4,492
Exons	719,821
Average exon length (bp)	229
Proteins	68,899
Average protein length (aa)	462
Gene density (No. genes/100 kb)	7.02
Protein-coding genes	2.61
Noncoding genes	4.41

The number of predicted genes was significantly lower compared to the one reported for the draft genome (60,717) [23]. The draft nature of the previous genome could be a factor influencing prediction, because the genome was assembled in more than 20,000 scaffolds and the QV was low, and predictions can become fragmented and/or truncated, overinflating the number of genes. This is in line with our new results, as even though the number of predicted genes for the chromosome-level assembly is lower (34,862), this prediction is more complete, as evidenced by a (i) 2.3-fold increase in the number of BUSCO genes found (Supplementary Table S4), (ii) decrease of duplicated and missing BUSCOs, and (iii) 1.8-fold increase in the number of RNA-seq reads mapped (Supplementary Data Note 1–Table 1).

### Functional annotation

**Figure 3:**
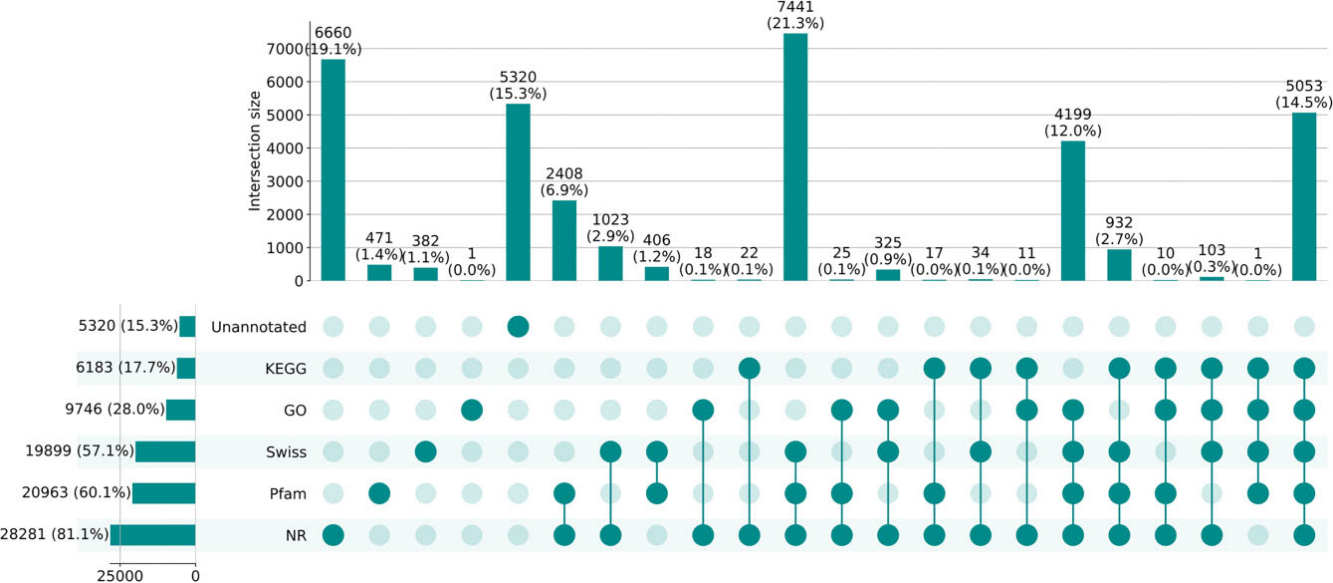
UpSetPlot representing the different functional annotations. Horizontal bars on the left represent the total number of genes annotated according to each database. Vertical bars represent overlapping annotations (i.e., genes annotated by a single or a combination of databases), as indicated by the connected dark green circles.

The longest protein inferred from each gene was selected using the primary_transcript.py script from OrthoFinder (RRID:SCR_017118) v2.5.4 [39]. These proteins were aligned against the SwissProt database (downloaded on 2 June 2022) using BLASTP v2.12.0+ from blast+ package [40] and against the NR database (downloaded on 24 June 2022) using Diamond v2.0.15.153 [41]. Both alignments were done using a threshold of 1e^−5^ for the e-value parameter. Out of the 34,862 protein-coding genes, 19,899 (57.08%) had at least 1 hit against the curated SwissProt database (Fig. 3). The eggNOG mapper v2 web server [42] was used to attribute Gene Ontology (GO) terms and KEGG pathways to each gene. At least 1 GO term and at least 1 KEGG pathway were associated with 9,746 (27.96%) and 6,183 (17.74%) genes, respectively. To annotate protein domains, an alignment against Pfam (RRID:SCR_004726) was done using the hmmsearch (e-value threshold of 1e^−5^) command from HMMER v3.3.1 [43]. A total of 20,963 (60.13%) genes were associated with at least 1 protein domain. Sequences were labeled as “unannotated” when they did not have a hit to any of the 5 databases searched (NR, SwissProt, GO, KEGG, and Pfam).

### Comparative genomics with other mollusks

Seven bivalves and 1 gastropod (*Pomacea canaliculata*) species were chosen to search for orthologs to the golden mussel genes (Supplementary Table S5). All proteomes were processed with OrthoFinder's primary_transcript.py script to retrieve only the longest protein associated with each gene. The processed proteomes were then used as input to run OrthoFinder v2.5.4 [39] with default parameters.

**Figure 4:**
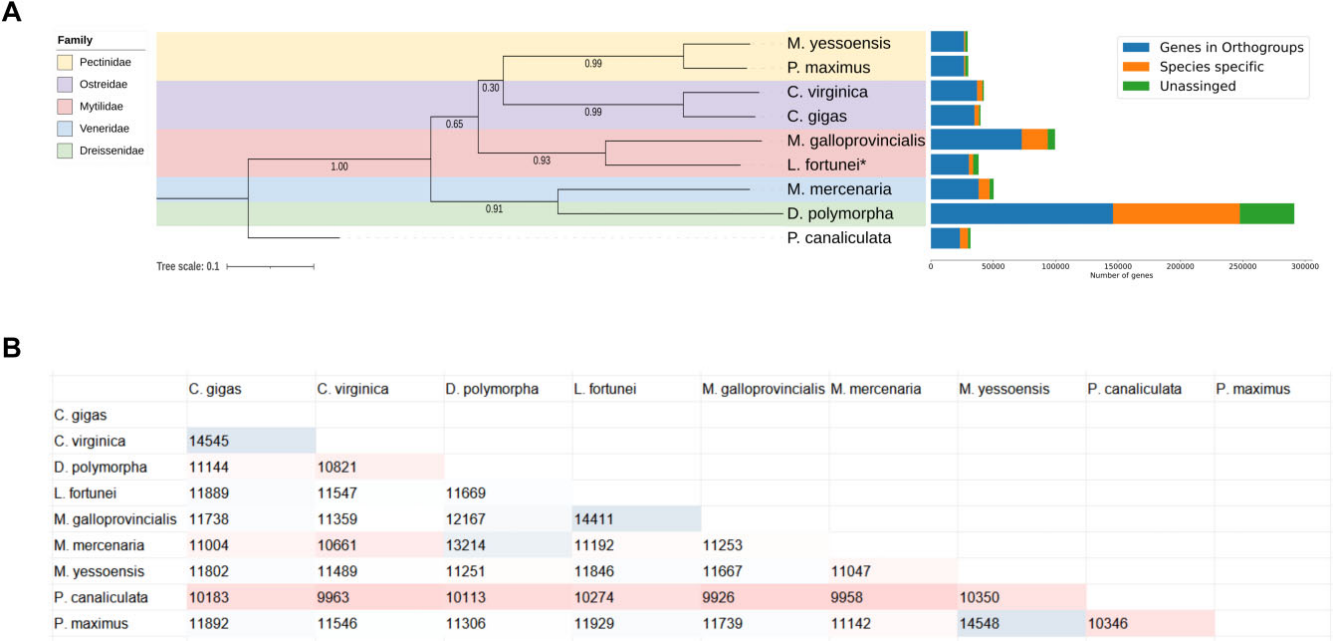
OrthoFinder results for the 9 mollusk species studied. (A) (left) Species tree constructed based on the inferred orthogroups and (right) gene counts for different categories. (B) Number of genes shared between each pair of species. The darker the red, the smaller the number of shared genes, while the darker the blue, the greater their number.

Overall, OrthoFinder was able to assign 436,439 genes to orthogroups, representing 86.9% of all mollusks’ genes (Supplementary Table S6). The species tree, built with STAG based on the orthogroups, placed species in the expected families, with the gastropod *P. canaliculata* used as the outgroup to root the tree (Fig. 4A). Most species had a high proportion of genes assigned to orthogroups, with *Dreissena polymorpha* showing an inflated number of genes (Fig. 4A and Supplementary Table S7). Of all 50,219 orthogroups identified, 7,616 (15.2%) had genes from all 9 mollusk species (Supplementary Table S6). For the golden mussel, 30,508 genes (87.5%) were assigned to an orthogroup, with 823 orthogroups assigned as golden mussel specific (Supplementary Table S7). As expected, the species that shared the largest number of genes (14,411) with the golden mussel was *Mytilus galloprovincialis*, which belong to the same family (Mytilidae) (Fig. 4B).

### Hemizygosity investigation

**Figure 5:**
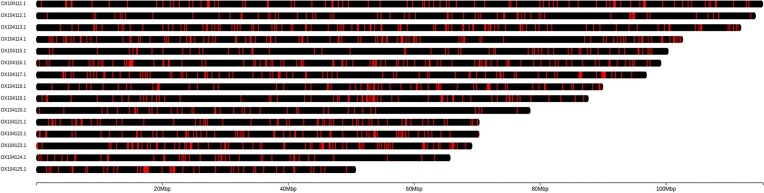
Distribution of hemizygous regions over the 15 chromosome-level scaffolds. The vertical red lines represent the location of the hemizygous regions.

We searched for hemizygous regions in the golden mussel genome applying the pipeline of Calcino et al. [44] for structural variant detection with a few modifications to use HiFi reads in the analyses (protocol in Supplementary Data Note 2). The pipeline maps reads back to the reference and identifies structural variations using *pbsv* [45] and further scripts. Hemizygous regions can be insertions (subset of reads that have a sequence that is not present in the reference) or deletions (where the reference has a sequence not present in a subset of the mapped reads). Considering only the detected deletions, the percentage of the golden mussel genome flagged as hemizygous was 6.48%, which is in the range observed for other molluscan species (0.17–6.69%) [56]. If we also consider insertions, the hemizygous content increases to 9.79%, which is also in the range of other molluscan species (0.37–10.81%) [56]. The chromoMap package was used to plot the distribution of the hemizygous regions over the chromosome-level scaffolds (more details in Supplementary Data Note 2). As observed in other molluscan species, the hemizygous regions were widespread and not restricted to specific chromosomes or chromosomal regions (Fig. 5) [56].

**Figure 6:**
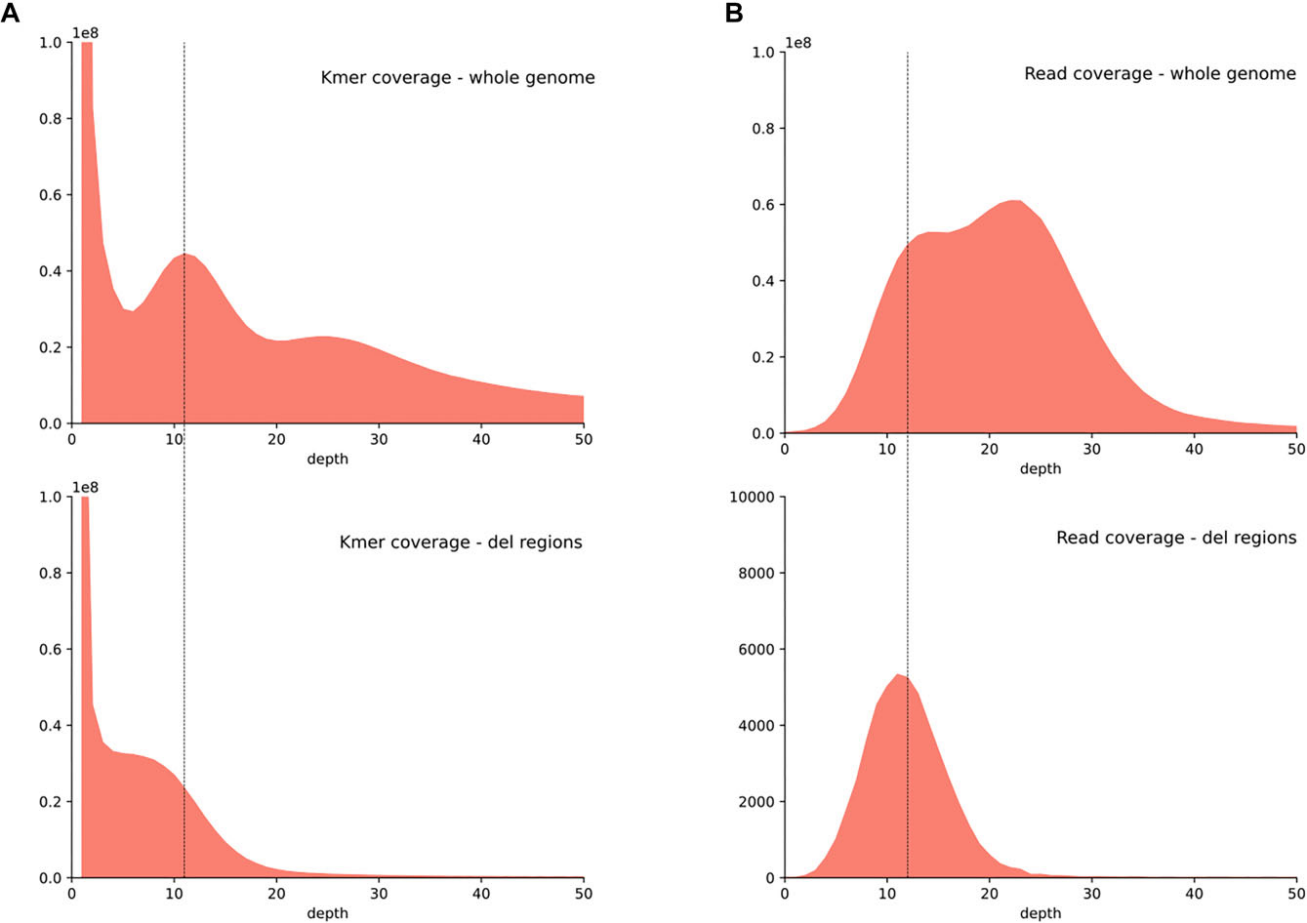
Analysis of *k*-mer and read coverage of hemizygous regions. (A) The *k*-mer plots represent the *k*-mer counts for a *k* = 21. The upper plot was built from all reads mapped to the genome, while the lower plot was built using only reads mapped to hemizygous (more specifically, deletions) regions. (B) The read coverage plots were built from a median read coverage calculation of 1-Kp windows. The upper plot represents the coverage over the whole genome and the lower plot over the hemizygous regions. For all plots, the black vertical lines represent the 1n coverage peak.

A *k*-mer count analysis of the sequences in hemizygous regions was performed. A *k*-mer coverage plot was built for (i) the reads mapped to the whole genome (i.e., to any genomic region) and (ii) the subset of reads that mapped only to the hemizygous regions (Supplementary Data Note 2). The mapped reads used for *k*-mer coverage analysis were also employed to calculate the read coverage (over sliding windows of 1 kb) of hemizygous regions and to compare it with the read coverage over the whole genome (Supplementary Data Note 2). For both analyses (*k*-mers and read coverage), we see hemizygous reads falling in the coverage of the heterozygous (1n) regions when compared with the whole-genome analysis (Fig. 6), affirming that they occur only in 1 haplotype of the assembly.

### DMRT gene family analysis

In addition to the 7 bivalve species used in the orthology inference analysis, 7 non-bivalve model organisms were chosen to search for potential DMRT genes (Supplementary Table S8). Those species were included because they already have well-characterized DMRT genes that could be used to guide the interpretation of the phylogeny. All non-bivalve and bivalve proteomes were processed with the primary_transcripts.py script to get a single (the longest) protein per gene. The processed proteomes were aligned against the Pfam-A database to annotate protein domains. The alignment was done using the hmmscan command from the HMMER (RRID:SCR_005305) v3.1b2 program [43] with a threshold value of 1e^−5^ for the -E parameter. After protein domain annotation, all proteins that had one of the following domains were selected as potential DMRT genes: DM (PF00751), DMA (PF03474), DMRT-like (PF15791), or Dmrt1 (PF12374). Additionally, MAB-3 sequence from *Caenorhabditis elegans* (Uniprot Accession O18214) was included due to its well-established role in sex differentiation. The potential DMRT proteins were aligned using the clustalw command from CLUSTAL v2.1 [45], and the alignment was trimmed using the trimAl tool (RRID:SCR_017334) v1.4 [46] with the “-automated1” option. After an initial phylogeny tree inference, sequences belonging to clades with no bivalve sequences were removed. Manual inspection to check for remaining isoforms and split gene models was also carried out (Supplementary Data Note 3). The remaining proteins were aligned and trimmed using CLUSTAL and trimAI, followed by manual inspection of the trimmed alignment. The VG+I+G4 model was chosen according to ModelTest-NG v0.1.7 [47] and used to build the final tree with MrBayes (RRID:SCR_012067) v3.2.7a [48, 49] for 10,000,000 Markov Chain Monte Carlo (MCMC) generations. Convergence was evaluated by checking if the standard deviation of split frequencies was <0.01. The consensus tree was then manipulated using iToL (RRID:SCR_018174) [50, 51] to generate the final figure.

**Figure 7:**
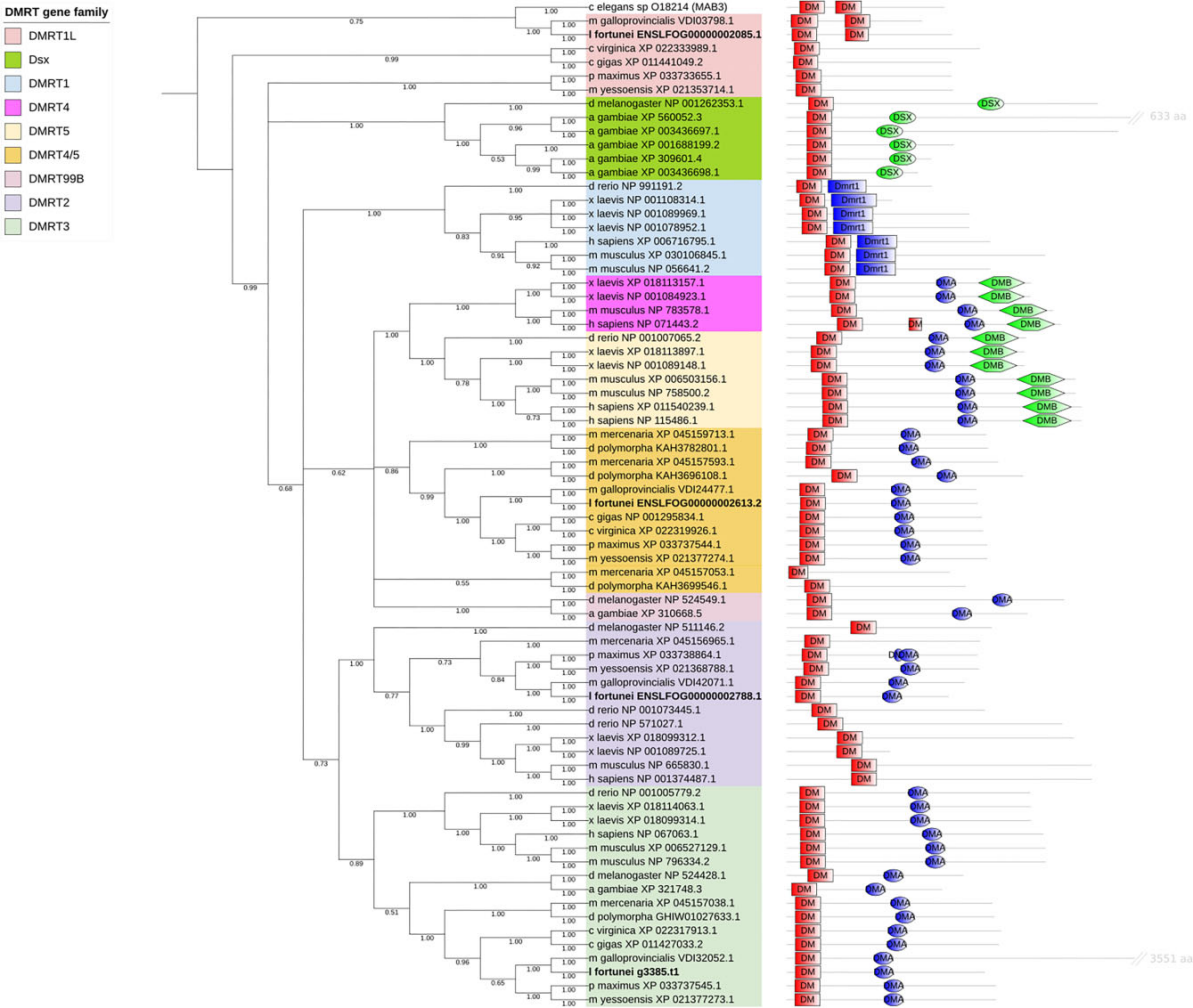
Phylogenetic tree of DMRT genes. Golden mussel genes are marked in bold. The domain representation of the *M. galloprovincialis* gene (VDI32052.1) was shortened (represented by a double slash) due to its significantly larger length for better visualization.

The final DMRT tree was midrooted since no a priori outgroup could be set. Bivalve orthologs to DMRT1L, DMRT2, DMRT3, and DMRT4/5 genes were found (Fig. 7). The golden mussel genome contains a single copy for each of the 4 DMRT genes, as well as *Mytilus galloprovincialis, Mizuhopecten yessoensis*, and *Pecten maximus*. A single DMRT2 gene was found in all bivalve species, except for *Crassostrea gigas, Crassostrea virginica*, and *Dreissena polymorpha*, for which no DMRT2 gene was found (Fig. 7; Supplementary Table S9). While DMRT2 genes in vertebrates and insects have shown a single DM domain, most bivalve DMRT2 genes have also shown a C-terminal DMA domain.

After manual correction of a false duplication in *D. polymorpha* (details in Supplementary Data Note 3), DMRT3 genes were found in a single copy in all bivalve species. Just like vertebrate and insect genes, DMRT3 from bivalves have both a DM and a DMA domain. DMRT4/5 genes were also found in single copy in all species, except *D. polymorpha* and *Mercenaria mercenaria*, in which 3 potential DMRT4/5 genes were identified. Most bivalve DMRT4/5 genes have a DM and a DMA domain, except a gene from *D. polymorpha* (KAH3699546.1) and a gene from *M. mercenaria* (XP_045157053.1) that are evolutionarily more distant to the other bivalve DMRT4/5 genes and could therefore represent a different DMRT gene type.

After manual removal of a false duplication in *C. virginica* (details in Supplementary Data Note 3), the DMRT1L genes were found in single copy in all bivalve species except in *M. mercenaria* and *D. polymorpha*, where DMRT1L seems to be missing. Bivalve DMRT1L genes missed both the Dmrt1 domain (vertebrate related) and the Dsx domain (insect related), containing only DM domains. Some bivalve DMRT1L genes contain a single DM domain, while others (e.g., the golden mussel) contain 2 DM domains, like MAB-3 from *C. elegans*. DMRT1L bivalve sequences were split into 3 monophyletic clades: (i) a clade containing Mytilidae (*L. fortunei* and *M. galloprovincialis*) sequences, (ii) another clade containing genes from *C. virginica* and *C. gigas*, and (iii) a clade containing sequences from *P. maximus* and *M. yessoensis*. All DMRT1L clades contained genes whose expression was shown to be male biased. *C. gigas* DMRT1L has shown to have significantly higher expression in male gonads [19], the same pattern observed for *M. yessoensis* [20]. Regarding the golden mussel, a DMRT-like transcript (GGt_299830_c0_g1_i1) has shown to have male-biased expression in the gonads [52]. We have aligned that transcript against the chromosome-level genome of the golden mussel and verified that it matches the ENSLFOG00000002085.1 gene, which is part of the putative DMRT1L clade. Despite the relevant changes in the sequences of the DMRT1L genes in different bivalve species, it seems that they have kept the characteristic feature of having male-biased expression, which we assume has to do with their role in male sex differentiation.

### Reuse potential

In this study, we present a chromosome-level genome for the golden mussel. The high quality and contiguity of this genome will benefit downstream studies that focus on either studying individual gene families of interest or genomic evolution at the chromosome level. One project that will immediately benefit from the new genome is a biotechnology-based solution to control invasive golden mussel populations, which is under development [6]. In the current study, we have identified a putative sex determination/differentiation gene (DMRT1L) in the golden mussel that stands out as a potential target for the control strategy. Further studies should be conducted to confirm that DMRT1L disruption induces incapacity of male golden mussels to sexually develop.

However, a reference genome based on the sequencing of a single specimen does not encompass the genetic diversity of the species. This limitation can compromise the development of efficient biotechnology-based control solutions that rely on specific target gene sequences. For example, single-nucleotide polymorphisms (SNPs) at the target site of a CRISPR-Cas9–based gene drive strategy can confer resistance to Cas9 cleavage, rendering the control strategy ineffective [53]. To identify potential critical SNPs and select invariant target sites, it will be necessary to resequence regions of interest in multiple individuals.

Structural variants have been detected in other mollusc species. In those species, a pattern of presence/absence variation (PAV) has been reported, in which some genes are present only in some individuals of the population [44]. The chromosome-level genome may be used as a reference for future studies resequencing multiple golden mussel individuals to check whether the species is also under PAV and, if so, which parts of the genome are more or less conserved between individuals. Ultimately, only genes that are not subject to PAV should be considered potential targets for biotechnology-based control strategies.

The chromosome-level genome can also be used as a reference for future population genomic studies. Understanding genomic variation among different golden mussel populations may unveil the routes of dispersion in invaded areas and support better control policies. Besides that, the new genome will support the study of chromosome evolution within Lophotrochozoan and Mytilidae through the comparison to chromosome-level genomes of related species. Lastly, the higher contiguity and accuracy of the new genome greatly benefits gene prediction quality aiming for more reliable studies of the evolution of genes and gene families.

## Discussion

The new reference genome for *L. fortunei* reported in this study has better contiguity, completeness, and accuracy metrics compared to the draft assembly, meaning that it is a more complete and reliable resource of information for the study of the golden mussel. Previous studies have shown that highly fragmented draft genomes can contain errors even in coding regions, jeopardizing experimental and *in silico* studies that use its sequences as a reference. For instance, Korlach et al. [54] have shown that the draft genome of 2 avian species had a series of misassemblies that generated issues (e.g., missing sequences and base call errors) in coding sequences and/or its flanking regions, and those issues could be resolved after a new assembly based on PacBio long reads. The high-quality reference genome reported in this study increases the accuracy and completeness of genes of interest for the study of the golden mussel, supporting both fundamental and applied research on this invasive species. In addition to that, the high contiguity of the assembly opens the door to comparative studies at a chromosome scale, shedding light on the evolution of the golden mussel and other genomes.

Structural variation analysis showed that a significant (6.48%) proportion of the golden mussel genome was in a hemizygous state (i.e., the region is present in only one of the homologous chromosomes). The presence of hemizygous regions has already been seen in other molluscan species [44, 55]. Compared to the 8 molluscan species analyzed in Calcino et al. [44], the golden mussel showed the second largest proportion of hemizygosity, only lower than the bivalve *Scapharca* (*Anadara*) *broughtonii* (6.69%). In another Mytilidae species (*M. galloprovincialis*), it has been shown that the presence of hemizygous regions is correlated with the occurrence of gene PAV [55], which, as discussed in the “Reuse potential” section, may have impacts on biotechnology-based control strategies. Future resequencing studies should allow us to move from a genome to a pangenome scenario and to check what set of golden mussel genes (if any) is under PAV.

The DMRT gene family is known for its role in sex determination and differentiation, and it has been proposed as a target for biotechnological population control strategies in the malaria mosquito [8]. Using the chromosome-level genome assembled in this study, we have done the first genome-wide characterization of the DMRT gene family in the golden mussel, and we were able to identify DMRT1L, DMRT2, DMRT3, and DMRT4/5 orthologs. DMRT2/DMRT11E genes show varying functions. In mouse, DMRT2 is involved in axial skeleton development, while in zebrafish, DMRT2a/2b play roles in left–right patterning [56]. However, in arthropods, DMRT11E has shown to play a role in sex differentiation. Knockdown of *Drosophila melanogaster* DMRT11E causes sperm malformation [57], while DMRT11E is required for proper oogenesis in the silkworm *Bombyx mori* [58]. DMRT2 function in mollusks is still unclear, but studies of expression profiles suggest its participation in spermatogenic cell differentiation in the pearl oyster *Pinctada fucata* and in the scallop *Chlamys nobilis* [59, 60].

In mammals, DMRT3 plays a role in neurogenesis, with mutations associated with locomotion problems in horses and spinal circuit malfunction in mice [61]. DMRT3 has high expression in testis in some mammalian and fish species, suggesting a potential role in testicular development [62, 63]. DMRT4/5/99B genes have a well-conserved function in different species being mainly involved in neurogenesis. Mutations of DMRT4 and DMRT5 in vertebrates cause neuronal abnormalities [64–66], just like mutations do in the DMRT99B in arthropods [67, 68]. As far as we know, no mutation study has been carried out on mollusks to explore DMRT5 function, although its tissue-wide distribution and expression indicates it may play a role in early embryonic development and various biological processes in *C. nobilis* [59].

Dsx (arthropods), MAB-3 (nematodes), and DMRT1 (vertebrates) genes are members of the DMRT family historically associated with sex determination and differentiation roles [15, 69, 70]. Although sharing the same function, there is some debate as to whether those genes share a common ancestor. Based on phylogenetic and synteny analyses, Mawaribuchi et al. [71] concluded that those 3 genes form separate clusters and therefore might have emerged independently in each clade. The phylogenetic analysis for the DMRT family in our study is in agreement, with the addition of a cluster of sex differentiation genes specific to mollusks named DMRT1L. Those genes consistently share a pattern of male-biased expression in the gonads in many other mollusk species [19–21, 72], and a recent study has confirmed that knockdown of the DMRT1L in *C. gigas* causes male gonads to fail to differentiate [73]. If DMRT1L knockdown in the golden mussel shows the same consequences, it can be a strong target for population control strategies of this invasive species.

## Supplementary Material

giad072_GIGA-D-22-00343_Original_Submission

giad072_GIGA-D-22-00343_Revision_1

giad072_Response_to_Reviewer_Comments_Original_Submission

giad072_Reviewer_1_Report_Original_Submission Marco Gerdol -- 2/12/2023 Reviewed

giad072_Reviewer_1_Report_Revision_1 Marco Gerdol -- 6/13/2023 Reviewed

giad072_Reviewer_2_Report_Original_Submission Qingzhi Wang -- 2/14/2023 Reviewed

giad072_Supplemental_Files

## Figures and Tables

**Table 6:** A list of the Wellcome Sanger Tree of Life contributors to this Data Note. The author list can also be found in *Zenodo* [75]

**Programme Lead:** Mark Blaxter
**Associate Director: Delivery and Operations:** Ed Symons
**Head of Production Genomics:** Kerstin Howe
**Tree of Life Samples**
Nancy Holroyd, Edel Sheerin, Sophie Potter, Catherine McCarthy
**Tree of Life Laboratory**
Lead: Caroline Howard
Adam Bates, Isabelle Clayton-Lucey, Amy Denton, Andrew Griffiths, Benjamin Jackson, Haddijatou Mbye, Graeme Oatley, Juan Pablo Narváez Gomez, Liam Prestwood, David Rowland, Abitha Thomas, Aarushi Vaidya
**Tree of Life Assembly**
Lead: Shane A. McCarthy
Eerik Aunin, William Eagles, Noah Gettle, Ksenia Krasheninnikova, Eugene Myers, Damon-Lee Pointon, Ying Sims, James Torrance, Marcela Uliano-Silva, Chenxi Zhou
**Genome Reference Informatics Team**
Lead: Jonathan Wood
Dominic Absolon, Joanna Collins, Michael Paulini, Sarah Pelan, Alan Tracey, Bethan Manley

## Data Availability

The genome sequence is available in the NCBI under accession GCA_944474755.1, while contigs representing the alternative haplotype are available as GCA_944589985.1. Raw data accessions are summarized in Table 5. All supporting data are available in the *GigaScience* GigaDB database [74]. Accession numbers of raw sequencing data used for the genome assembly project

## Additional Files

**Supplementary Data Note 1**. RNAseq mapping against gene models.

**Supplementary Data Note 2**. Hemizygosity investigation.

**Supplementary Data Note 3**. Manual curation of DMRT gene models.

**Supplementary Fig. S1**. GenomeScope profile built from PacBio HiFi data.

**Supplementary Fig. S2**. Merqury plots for (A) the chromossome-level genome and (B) the chromosome-level genome concatenated with its haplotigs.

**Supplementary Table S1**. General statistics for the golden mussel genome sequence.

**Supplementary Table S2**. Run accessions for the RNA-seq dataset used to support the gene prediction for the chromosome-level genome.

**Supplementary Table S3**. Number of proteins associated with each gene.

**Supplementary Table S4**. BUSCO statistics of gene models from the draft and from the chromosome-level genome assembly.

**Supplementary Table S5**. Mollusk species selected for the OrthoFinder analysis.

**Supplementary Table S6**. Overall OrthoFinder statistics.

**Supplementary Table S7**. Per species OrthoFinder statistics.

**Supplementary Table S8**. Non-bivalve proteomes selected to search for potential DMRT genes.

**Supplementary Table S9**. DMRT genes found for each bivalve species analyzed.

## Abbreviations

BUSCO: Benchmarking Universal Single-Copy Orthologs; DMRT: Doublesex and Mab-3 related transcription factor; GO: Gene Ontology; GRIT: Genome Reference Informatics Team; KEGG: Kyoto Encyclopedia of Genes and Genomes; LINE: long interspersed nuclear element; lncRNA: long noncoding RNA; LTR: long terminal repeat; misc_RNA: miscellaneous RNA; PAV: presence/absence variation; RNA-seq: RNA sequencing; RPTP: receptor-type protein tyrosine phosphatase; rRNA: ribosomal RNA; scaRNA: small Cajal body-specific RNA; SINE: short interspersed nuclear element; snRNA: small nuclear RNA; snoRNA: small nucleolar RNA; tRNA: transfer RNA; WSI: Wellcome Sanger Institute; VGP: Vertebrates Genome Project.
