# Supplementary material for: A chromosome-level assembly supports genome-wide investigation of the DMRT gene family in the golden mussel (Limnoperna fortunei)
Source: Gigascience. 2023 Sep 30;12:giad072. doi: 10.1093/gigascience/giad072 (PMC10541798; doi:10.1093/gigascience/giad072)
Supplement: giad072_GIGA-D-22-00343_Original_Submission [file giad072_giga-d-22-00343_original_submission.pdf]

GigaScience

A chromosome-level assembly supports genome-wide investigation of the DMRT gene family in the golden mussel (Limnoperna fortunei)

--Manuscript Draft--

**Manuscript Number:**	GIGA-D-22-00343
**Full Title:**	A chromosome-level assembly supports genome-wide investigation of the DMRT gene family in the golden mussel (Limnoperna fortunei)
**Article Type:**	Data Note
**Funding Information:**	Agência Nacional de Energia Elétrica (PD-10381-0419/2019): Not applicable<br>Coordenação de Aperfeiçoamento de Pessoal de Nível Superior: Mr João Gabriel Rodinho Nunes Ferreira; Mr Fábio Sendim; Ms. Yasmin R. da Cunha
**Abstract:**	Background<br>The golden mussel (Limnoperna fortunei) is a highly adaptive species that causes environmental and socioeconomic losses in invaded areas. Reference genomes have proven to be a valuable resource for studying the biology of invasive species. While the current golden mussel genome has been useful for identifying new genes, its high fragmentation hinders some applications.<br>Findings<br>In this study, we provide the first chromosome-level reference genome for the golden mussel. The genome was built using PacBio HiFi,10X and Hi-C sequencing data. The final assembly contains 99.4% of its total length assembled to the 15 chromosomes of the species and a scaffold N50 of 97.05 Mb. A total of 34 862 protein-coding genes were predicted, of which 84.7% were functionally annotated. We performed Orthofinder and CAFE v5 analyses across mollusks and found expanded gene families involved in structural and defense mechanisms. Using the new genome, we have performed a genome-wide characterization of the DMRT gene family, which has been proposed as a target for population control strategies in other species.<br>Conclusions<br>From the applied research perspective, a higher quality genome will support genome editing with the aim of developing biotechnology-based solutions to control the invasion. From the basic research perspective, the new genome may be used as a reference for future resequencing studies to assess the genomic variation among different golden mussel populations, unveiling potential routes of dispersion and helping to establish better control policies.
**Corresponding Author:**	Juliana Alves Americo, Ph.D<br>Bio Bureau Biotecnologia<br>Rio de Janeiro, RJ BRAZIL
**Corresponding Author Secondary Information:**	
**Corresponding Author's Institution:**	Bio Bureau Biotecnologia
**Corresponding Author's Secondary Institution:**	
**First Author:**	João Gabriel Rodinho Nunes Ferreira, MSc
**First Author Secondary Information:**	
**Order of Authors:**	João Gabriel Rodinho Nunes Ferreira, MSc
	Juliana Alves Americo, Ph.D
	Danielle L. A. S. do Amaral, Ph.D
	Fábio Sendim, MSc
	Yasmin R. da Cunha, BSc

Powered by Editorial Manager® and ProduXion Manager® from Aries Systems Corporation

	Darwin Tree of Life Consortium<br>Marcela Uliano-Silva, Ph.D<br>Mauro de Freitas Rebelo
**Order of Authors Secondary Information:**	
**Additional Information:**	
**Question**	**Response**
Are you submitting this manuscript to a special series or article collection?	No
**Experimental design and statistics**<br><br>Full details of the experimental design and statistical methods used should be given in the Methods section, as detailed in our Minimum Standards Reporting Checklist. Information essential to interpreting the data presented should be made available in the figure legends.<br><br>Have you included all the information requested in your manuscript?	Yes
**Resources**<br><br>A description of all resources used, including antibodies, cell lines, animals and software tools, with enough information to allow them to be uniquely identified, should be included in the Methods section. Authors are strongly encouraged to cite Research Resource Identifiers (RRIDs) for antibodies, model organisms and tools, where possible.<br><br>Have you included the information requested as detailed in our Minimum Standards Reporting Checklist?	Yes
**Availability of data and materials**<br><br>All datasets and code on which the conclusions of the paper rely must be either included in your submission or deposited in publicly available repositories (where available and ethically	Yes

appropriate), referencing such data using a unique identifier in the references and in the “Availability of Data and Materials” section of your manuscript.<br><br>Have you have met the above requirement as detailed in our Minimum Standards Reporting Checklist?	

Manuscript

Click here to access/download;Manuscript;Ferreira_et_al_2022.docx

A chromosome-level assembly supports genome-wide investigation of the DMRT gene family in the golden mussel (*Limnoperna fortunei*)

João Gabriel R. N. Ferreira[1,2], Juliana A. Americo[1&], Danielle L. A. S. do Amaral[1], Fábio Sendim[1,2], Yasmin R. da Cunha[1,2], The Darwin Tree of Life Project Consortium, Marcela Uliano-Silva[3]* & Mauro de F. Rebelo[2]*

[1] Bio Bureau Biotecnologia, Rio de Janeiro, Brazil,
[2] Instituto de Biofísica Carlos Chagas Filho, Universidade Federal do Rio de Janeiro, RJ, Brazil,
[3] Wellcome Sanger Institute, Hinxton, United Kingdom
[&] Corresponding author
*Contributed equally to this work

Abstract

Background

The golden mussel (*Limnoperna fortunei*) is a highly adaptive species that causes environmental and socioeconomic losses in invaded areas. Reference genomes have proven to be a valuable resource for studying the biology of invasive species. While the current golden mussel genome has been useful for identifying new genes, its high fragmentation hinders some applications.

Findings

In this study, we provide the first chromosome-level reference genome for the golden mussel. The genome was built using PacBio HiFi,10X and Hi-C sequencing data. The final assembly contains 99.4% of its total length assembled to the 15 chromosomes of the species and a scaffold N50 of 97.05 Mb. A total of 34 862 protein-coding genes were predicted, of which 84.7% were functionally annotated. We performed Orthofinder and CAFE v5 analyses across mollusks and found expanded gene families involved in structural and defense mechanisms. Using the new genome, we have performed a

genome-wide characterization of the DMRT gene family, which has been proposed as a target for population control strategies in other species.

Conclusions

From the applied research perspective, a higher quality genome will support genome editing with the aim of developing biotechnology-based solutions to control the invasion. From the basic research perspective, the new genome may be used as a reference for future resequencing studies to assess the genomic variation among different golden mussel populations, unveiling potential routes of dispersion and helping to establish better control policies.

Keywords

Golden mussel; *Limnoperna fortunei*; genome; invasive species; sex differentiation; DMRT

Data description

Context

Limnoperna fortunei — popularly known as the golden mussel — is a freshwater bivalve species native to Southeast China which has successfully established itself as an invasive species in other Asian countries (Cambodia, Japan, Laos, South Korea, Taiwan, and Thailand) and in several South American countries (Argentina, Brazil, Paraguay, and Uruguay) [1]. Because of its impact on ecosystem structure and function, the golden mussel is considered an efficient ecosystem engineer, and its establishment is associated with changes in local biodiversity and nutrient recycling [2,3]. Socioeconomic impacts are also relevant where golden mussel aggregates bind and obstruct net cages and equipment of hydroelectric power plants [4,5]. In the Brazilian hydroelectric sector alone it is estimated that the golden mussel causes an annual 120 million dollar loss due to longer and more frequent stops for maintenance [6]. Current control strategies have proven to be ineffective and the species has continued to spread. Alternative

biotechnological solutions have been proposed [6] and one possibility is to apply molecular tools to disrupt genes involved in the reproductive behavior. This has been tested in other species, such as the malaria mosquito, whose disrupted genotype is rapidly spreading through the population using a gene drive system [7,8].

The Doublesex and Mab-3 related transcription factor (DMRT) gene family is well conserved in animals and contains members that play important roles in sexual differentiation. DMRT genes have a conserved zinc finger DNA binding domain named DM and most animals contain multiple DMRT genes, which act in developmental processes such as somitogenesis, neurogenesis and gametogenesis [9–11]. The doublesex (Dsx) gene is present in insects and is required for both male and female sexual differentiation according to the sex-specific isoform that is produced after alternative splicing [12–14]. In nematodes, the Mab-3 gene acts as a critical factor for male sex determination [15,16], just like DMRT1 in vertebrates, which is required for somatic cells masculinization [17,18]. In mollusks, it is assumed that DMRT1L genes are involved in male sex differentiation, given the male-biased expression pattern in the gonads shared by many different species [19–21].

Reference genomes are an important resource for the study of invasive species. They have been used to study invasion dynamics, identifying molecular mechanisms conferring adaptiveness as well as promising genes for biotechnology-based control strategies [22]. There currently is a genome assembly for the golden mussel [23], but it is a highly fragmented representation of the 15 chromosomes (2n=30) of the species [24,25] assembled mostly based on Illumina sequencing reads. Limitations of the Illumina-based genome constrain its applications in resequencing and comparative genomic studies and may lead to incomplete or erroneous gene models.

Recent advances in sequencing technologies and bioinformatics algorithms have made the development of high quality reference genomes scalable and affordable. In this

study, we present such a high quality genome developed for the golden mussel. Based on the new genome, we have identified expanded golden mussel gene families. We have also identified four DMRT genes in the golden mussel genome, which have been compared to DMRT genes from other bivalve species to study the evolution of this gene family in the class. One golden mussel DMRT is a putative sex differentiation gene showing male-biased expression in the gonads; therefore, it is a potential target for biotechnology-based control strategies. The new golden mussel genome is expected to be a valuable reference for future studies on the species.

Sample collection

Golden mussel specimens were collected from the Taquari River, São Paulo, Brazil (23°16'45.7"S 49°12'01.7"W) on March 17, 2021. Three representative specimens were deposited in the molluscan collection of the National Museum administered by the Federal University of Rio de Janeiro (identification numbers: IB UFRJ 19950, IB UFRJ 19952 and IB UFRJ 19954). Other specimens were taxonomically identified by Dr. Igor Christo Miyahira. Finally, a set of specimens had their tissues – gonads, adductor muscle, digestive gland, gills, and foot – dissected and preserved in dry ice at -80ºC until and during transportation to the Wellcome Sanger Institute (WSI) in Hinxton, Cambridgeshire, United Kingdom for further processing and sequencing.

DNA extraction

DNA extraction was performed at the WSI's Tree of Life laboratory. Golden mussel samples were weighed and disrupted using a Covaris cryoPREP Automated Dry Pulveriser that subjects tissue – gill tissue was selected – to multiple impacts until it becomes a fine powder. Twenty-five mg of this powder were used for DNA extraction and 50 mg were set aside for Hi-C. DNA extraction was performed using a Qiagen MagAttract HMW DNA extraction kit on a KingFisher APEX. Fifty nanograms of DNA were submitted to 10X sequencing with any low molecular weight DNA removed prior to

sequencing using a 0.8X AMpure XP purification kit. Similarly, prior to submission to PacBio sequencing, high molecular weight DNA was sheared to an average fragment size of between 12 kb and 20 kb using a MegaRuptor 3 (speed setting 30). The sheared DNA was purified by solid-phase reversible immobilization using AMpure PB beads with a 1.8X ratio of beads to sample. The concentration of sheared DNA was assessed using a Qubit Fluorometer with Qubit dsDNA High Sensitivity Assay kit and Nanodrop spectrophotometer, while the fragment size distribution was assessed using an Agilent FemtoPulse.

Sequencing

All sequencing libraries were constructed using DNA extracted from a single specimen, a female golden mussel identified as xbLimFort5. Pacific Biosciences HiFi circular consensus and Chromium 10X Genomics linked-reads sequencing libraries were constructed according to the manufacturers' instructions. Sequencing was performed by the Scientific Operations core at the Wellcome Sanger Institute on Pacific Biosciences SEQUEL II (HiFi) and Illumina NovaSeq (10X) instruments. Hi-C data were generated using the Arima v2.0 kit and sequenced on a NovaSeq 6000 instrument.

Genome assembly

The genome assembly pipeline is summarized in Figure 1. The initial set of contigs was assembled using HiFiasm v0.16.1 combining HiFi and Hi-C reads in the Hi-C integrated mode [26]. 10X linked-reads were mapped to contigs using LongRanger v2.2.2 [27] and then Freebayes v1.3.1 [28] was used to polish the contigs based on the 10X mapping. The polished contigs were then scaffolded using the YaHS pipeline v1.0 [29]. Finally, scaffolds were manually curated by WSI's Genome Reference Informatics Team (GRIT) following the protocol described by Kerstin and colleagues (2021) [30]. The curated scaffolds represent the final genome assembly, which was then annotated using

Ensembl Rapid Annotation Pipeline [31]. The mitochondrial genome was assembled using the MitoHiFi pipeline [32].

Figure 1. Genome assembly pipeline.

Completeness stats were calculated using BUSCO [33] version 5.0 with two datasets: metazoa_odb10 and mollusca_odb10. QV statistics was calculated using the Merqury software [34] using HiFi data (for the new genome) and Illumina paired-end data (for the Illumina-based genome).

The size of the final genome assembly is 1.34 Gb (Supplementary Table S1); 99.24% of its total length is distributed over the 15 largest scaffolds (Figure 2), which correspond to the haploid chromosome number (n=15) of the species [24]. The largest contig and the largest scaffold are 8.3 Mb and 115 Mb long, respectively, and the genome GC content is 33.6%.

Figure 2. The genome landscape. A) Circos representation of the 15 chromosomes assembled in this study. Each track represents: i) the size of each chromosome, ii) the gene density, and the iii) repeat density over the chromosome sequences, calculated using a 2 Mb window size. B) Hi-C contact map with chromosomes displayed in size order from top to bottom and from left to right.

Table 1 presents genomic statistics of the Illumina-based assembly and the chromosome-level reference produced in this study. The chromosome-level reference scaffold N50 is 313-fold greater than its Illumina-based predecessor. An improvement has also been achieved in genome completeness as shown by an increase in the percentage of BUSCO genes. The QV score of 53 of the chromosome-level genome represents a base call accuracy of 99.999%. All the quality metrics calculated for the new assembly conform to the standards of the Vertebrates Genome Project (VGP) for what is considered a high-quality genome [35].

Table 1. Comparison of assembly metrics between the Illumina-based and the new golden mussel genome.

	Illumina-based genome (GCA_003130415.1)	Chromosome-level genome (GCA_944474755.1)

**Total assembly length (Gb)**	1.67	1.34
**GC content (%)**	33.6	33.8
**Number of scaffolds**	20 580	309
**Scaffold N50 (Mb)**	0.31	97.05
**Scaffold L50**	1 489	7
**Number of contigs**	61 175	1 838
**Contig N50 (Mb)**	0.03	1.50
**Contig L50**	16 521	277
**QV**	14.89	53.36
**BUSCO (metazoa_odb10)**	C:66.8% [S:65.0%,D:1.8%], F:19.3%,M:13.9%,n:954	C:95.6% [S:95.0%,D:0.6%], F:2.2%,M:2.2%,n:954
**BUSCO (mollusca_odb10)**	C:56.0% [S:54.7%,D:1.3%], F:7.4%,M:36.6%,n:5295	C:87.0% [S:86.0%,D:1.0%], F:3.2%,M:9.8%,n:5295

BUSCO statistics. C=complete; S=complete and single-copy; D=complete and duplicated; F=fragmented; M=missing; n=number of BUSCO genes from reference dataset.

Repeat annotation

Detection and classification of repeat elements was done using the Earl Grey pipeline v1.3 [36]. Earl Grey was run with the RepeatMasker search term (-r) set to “mollusca”. Almost half (46.93%) of the genome was annotated as repetitive sequences, with 35.80% of the genome labeled as unclassified repeats (Table 2). Similarly high proportions of unclassified repeats have been reported in other mussels [37,38]. The second most frequent repeat class detected was Long interspersed nuclear elements (LINE), representing 4.51% of the total genome.

Table 2. Repetitive elements identified in the golden mussel genome.

Classification*	Total sequence length (bp)	Sequences count	Proportion of genome (%)	Number of distinct classifications
DNA	46 269 487	64 938	3.46	201
LINE	60 245 208	64 949	4.51	224
LTR	28 342 781	50 395	2.12	112
Other (Simple Repeat, Microsatellite, RNA)	216 907	244	0.02	2
Penelope	11 115 342	24 065	0.83	23
Rolling Circle	1 640 436	1 503	0.12	6
SINE	831 095	723	0.06	3
Unclassified	478 115 783	883 466	35.80	1 494

LINE=Long interspersed nuclear elements; LTR=Long terminal repeats; SINE=Short interspersed nuclear element
* Classification in alphabetical order.

Gene prediction

A total of 34 862 protein-coding genes were predicted by the Ensembl rapid annotation pipeline [30], with 68 899 proteins inferred. Most (53.5%) genes were associated with a single protein, with about 21.8% associated with two proteins, and 24.7% with three or more proteins (Supplementary Table S2). In addition to the protein-coding genes, 58 911 non-coding genes were predicted, most of which (56.5%) were classified as long non-coding RNA (lncRNA) (Table 3).

Table 3. Categories of predicted genes.

Statistics	Value
Protein-coding genes	34 862
Non-coding genes	58 911
lncRNA	33 258
Y_RNA	9 316
tRNA	7 582
ribozyme	5 091
misc_RNA	1 641
rRNA	1 410
snRNA	565
snoRNA	47
scaRNA	1

lncRNA=long non-coding RNA; tRNA=transfer RNA; misc_RNA=miscellaneous RNA; rRNA=ribosomal RNA; snRNA=small nuclear RNA; snoRNA=small nucleolar RNA; scaRNA=small Cajal body-specific RNA.

Table 4. Gene prediction statistics.

Statistics	Value
Average gene length (bp)	9 426
Protein-coding genes (bp)	17 765
Non-coding genes (bp)	4 492
Exons	719 821
Average exon length (bp)	229
Proteins	68 899
Average protein length (aa)	462
Gene density (No. genes/100kb)	7.02
Protein-coding genes	2.61
Non-coding genes	4.41

Functional annotation

The longest protein inferred from each gene was selected using the primary_transcript.py script from OrthoFinder v2.5.4 [39]. Those proteins were aligned against the SwissProt database (downloaded on June 2, 2022) using BLASTP v2.12.0+ from blast+ package [40] and against the NR database (downloaded on June 24, 2022) using Diamond v2.0.15.153 [41]. Both alignments were done using a threshold of $1e^{-5}$ for the e-value parameter. Out of the 34 862 protein-coding genes, 19 899 (57.08%) had at least one hit against the curated SwissProt database (Figure 3). The eggNOG mapper v2 web server [42] was used to attribute GO terms and KEGG pathways to each gene. At least one GO term and at least one KEGG pathway was associated with 9 746 (27.96%) and 6 183 (17.74%) genes, respectively. To annotate protein domains, an alignment against Pfam was done using the *hmmsearch* (e-value threshold of $1e^{-5}$) command from HMMER v3.3.1 [43]. A total of 20 963 (60.13%) genes was associated with at least one protein domain. Sequences were labeled as “unannotated” when they did not have a hit to any of the five databases searched (NR, SwissProt, GO, KEGG and Pfam).

Figure 3. UpSetPlot representing the different functional annotations. Horizontal bars represent the total number of genes annotated according to each database. Vertical bars represent overlapping annotations (i.e. genes annotated by a single or a combination of databases), as indicated by the connected dark green circles.

Comparative genomics with other mollusks

Seven bivalves and one gastropod (*Pomacea canaliculata*) species were chosen to search for orthologs to the golden mussel genes (Supplementary Table S3). All proteomes were processed with OrthoFinder’s primary_transcript.py script to retrieve only the longest protein associated with each gene. The processed proteomes were then used as input to run OrthoFinder v2.5.4 [39] with default parameters.

Overall, OrthoFinder was able to assign 436,439 genes to orthogroups, representing 86.9% of all mollusks’ genes (Supplementary Table S4). The species tree built with STAG based on the orthogroups placed species in the expected families, with the

gastropod *Pomacea canaliculata* used as the outgroup to root the tree (Figure 4A). Most species had a high proportion of genes assigned to orthogroups, with *D. polymorpha* showing an inflated number of genes (Figure 4A and Supplementary Table S5). Of all 50 219 orthogroups identified, 7,616 (15.2%) had genes from all nine mollusk species (Supplementary Table S4). For the golden mussel, 30 508 genes (87.5%) were assigned to an orthogroup, with 823 orthogroups assigned as golden mussel specific (Supplementary Table S5). As expected, the species that shared the largest number of genes with the golden mussel was *Mytilus galloprovincialis*, with 14 411 genes (Figure 4B).

Figure 4. OrthoFinder results for the nine mollusk species studied. A) (left) Species tree constructed based on the inferred orthogroups and (right) gene counts for different categories. B) Number of genes shared between each pair of species. The darker the red the smaller the number of shared genes, while the darker the blue the greater their number.

Gene family evolution

CAFE [44,45] v5 was used to analyze changes in gene family size from OrthoFinder's orthogroups. Orthogroups were first filtered to remove the ones that had more than 100 genes for any species. OrthoFinder's species tree was turned into an ultrametric tree using the "make_ultrametric.py" script. CAFE was then run with the filtered orthogroups and the ultrametric species tree to infer an error model. CAFE was then rerun using the error model and testing different evolution models (base model and gamma model with different numbers of K categories); it was found that the most likely model was gamma at K=2 categories. Gene families found to expand at a significant rate according to CAFE results ($p<0.05$) were analyzed to search for enriched gene ontology (GO) terms. First, all golden mussel genes were annotated for GO terms with Interproscan version 5.59-91.0 [46]. Then, GOATOOLS [47] version 1.2.3 was used to detect GO terms significantly enriched ($p<0.05$ after Bonferroni correction) in the expanded dataset related to the complete gene dataset.

A total of 11 150 gene families were analyzed by CAFE for changes in gene family size after initial CAFE filtering of families with at least one gene at the root of the tree. Of the 407 expanded gene families in the golden mussel, 14 were predicted to be evolving at a significant ($p<0.05$) rate (Additional File 1). Of the 1,698 contracted gene families, 61 were predicted to be evolving at a significant rate (Additional File 2). The significantly expanded gene families were analyzed for gene ontology (GO) enrichment, which found the "protein dephosphorylation" (GO:0006470) term as enriched. The term mapped to genes from expanded orthogroups OG0000342 and OG0000049, both of which were annotated as "Receptor-type protein tyrosine phosphatases" according to alignment against the SwissProt database. Another enriched term from the expanded families was "cell-cell adhesion via plasma-membrane adhesion molecules" (GO:0098742), corresponding to the expanded orthogroup OG0000076, annotated as "Cadherin EGF LAG seven-pass G-type receptor" or "Protocadherin-like protein". Finally, the term "metalloendopeptidase activity" (GO:0004222) was also enriched in the expanded families set, being associated to an orthogroup (OG0000450) annotated as metalloproteases.

DMRT gene family analysis

In addition to the eight mollusk species used for the orthology inference analysis, seven non-mollusk model organisms were chosen to search for potential DMRT genes. Those species were included because they already have well characterized DMRT genes that could be used to guide the interpretation of the phylogeny (Supplementary Table S6). All non-mollusk and mollusk proteomes were processed with the primary_transcripts.py script to get a single (the longest) protein per gene. The processed proteomes were aligned against the Pfam-A database to annotate protein domains. The alignment was done using the *hmmscan* command from the HMMER v3.1b2 program [43] with a threshold value of $1e^{-5}$ for the -E parameter. After protein domain annotation, all proteins that had one of the following domains were selected as potential DMRT genes: DM

(PF00751), DMA (PF03474), DMRT-like (PF15791) or Dmrt1 (PF12374). Additionally, MAB3 sequence from *Caenorhabditis elegans* (Uniprot Accession O18214) was included due to its well established role in sex differentiation. The potential DMRT proteins were aligned using the clustalw command from CLUSTAL v2.1 [48] and the alignment was trimmed using the trimAl tool v1.4 [49] with the "-automated1" option. After an initial phylogeny tree inference, sequences belonging to non-mollusk clades (i.e., clades with no mollusk sequences) were removed. The remaining proteins were again aligned and trimmed using CLUSTAL and trimAI. The VG+I+G4 model was chosen according to ModelTest-NG v0.1.7 [50] and used to build the final tree with MrBayes v3.2.7a [51,52] for 10 000 000 MCMC generations. The consensus tree was then manipulated using iToL [53,54] to generate a final image of the tree.

The final DMRT tree was midrooted since no *a priori* outgroup could be set. Mollusk orthologs to DMRT1L, DMRT2, DMRT3 and DMRT4/5 genes were found (Figure 5). The golden mussel genome contains a single copy for each of the four DMRT genes, as well as *Mytilus galloprovincialis*, *Mizuhopecten yessoensis* and *Pecten maximus*. A single DMRT2 gene was found in all mollusk species, except for *Crassostrea gigas*, *Crassostrea virginica* and *Dreissena polymorpha,* for which no DMRT2 gene was found (Figure 5; Supplementary Table S7). While DMRT2 genes in vertebrates and insects have shown a single DM domain, most mollusk DMRT2 genes have also shown a C-terminal DMA domain.

DMRT3 genes were found in single copy in all species but *Dreissena polymorpha*, where two putative DMRT3 genes were found. Just like vertebrate and insect genes, DMRT3 from mollusks have shown both a DM and a DMA domain. The exceptions to this were two *D. polymorpha* sequences (KAH3721156.1 and KAH3721157.1). While KAH3721156.1 was missing the DMA domain, KAH3721157.1 was missing the DM domain. Those two sequences are neighbors in chromosome 13 (NC_068367.1) separated by a distance of 880 bp, therefore it is possible that they were incorrectly split

during annotation, but should instead be merged into a single gene, although further investigation is needed.

DMRT4/5 genes were also found in single copy in all species, except *D. polymorpha* and *Mercenaria mercenaria*, in which three potential DMRT4/5 genes were identified. Most mollusk DMRT4/5 genes have shown a DM and a DMA domain, except a gene from *D. polymorpha* (KAH3699546.1) and a gene from *M. mercenaria (*XP_045157053.1*)* that are evolutionarily more distant to the other mollusk DMRT4/5 genes and could therefore represent a different DMRT gene type. The DMRT4/5 from *Pomacea canaliculata* (XP_025110328.1) is also missing the DMA domain, raising the possibility that this domain may be absent in gastropods.

Figure 5. Phylogenetic tree of DMRT genes. Golden mussel genes are marked in bold. The domain representation of the *M. galloprovincialis* gene (VDI32052.1) was shortened (represented by a double slash) due to its significantly larger length for better visualization.

DMRT1L genes were found in all species but *M. mercenaria* and *D. polymorpha*. *Pomacea canaliculata* and *C. virginica* contained two putative DMRT1L genes, while the remaining mollusks contained a single DMRT1L gene. A manual inspection has shown that *C. virginica* genes actually mapped to the same loci in *C. virginica*'s genome (location: LOC111130972), i.e., they are actually isoforms from the same gene that OrthoFinder's script to remove isoform duplicates was not able to detect. Mollusks' DMRT1L genes missed both Dmrt1 domain (vertebrate-related) and Dsx domain (insect-related), containing only DM domains. Some mollusk DMRT1L genes contain a single DM domain, while others (e.g. the golden mussel) contain two DM domains, like the MAB3 from *Caenorhabditis elegans*. DMRT1L mollusk sequences were split into three monophyletic clades: i) a clade containing *Crassostrea sp.* genes and MAB3; ii) another clade containing genes from the golden mussel, *M. galloprovincialis* and *P. canaliculata* (gastropod); and iii) a clade containing sequences from *P. maximus* and *M. yessoensis*, which formed a monophyletic group with vertebrates DMRT1 genes. All clades contained

genes whose expression was shown to be male-biased. *C. gigas* DMRT1L has shown to have significantly higher expression in male gonads [19], the same pattern observed for *M. yessoensis [20]*. Regarding the golden mussel, a DMRT-like transcript (GGt_299830_c0_g1_i1) has shown to have male-biased expression in the gonads [55]. We have aligned that transcript against the chromosome-level genome of the golden mussel and verified that it matches the ENSLFOG00000002085.1 gene, which is part of the putative DMRT1L clade. Despite the relevant changes in the sequences of the DMRT1L genes in different mollusk species, it seems that they have kept the characteristic feature of having male-biased expression, which we assume has to do with their role in male sex differentiation.

Re-use potential

In this study, we present a chromosome-level genome for the golden mussel. The high quality and contiguity of this genome will benefit downstream studies that focus on either studying individual gene families of interest or genomic evolution at the chromosome level. One project that will immediately benefit from the new genome is the development of a biotechnology-based solution to control invasive golden mussel populations, which our group is currently developing [6]. In the current study, we have identified a putative sex determination/differentiation gene (DMRT1L) in the golden mussel that stands out as a potential target for the control strategy. Further studies should be conducted to confirm that DMRT1L disruption induces incapacity of male golden mussels to sexually develop.

Additionally, the new genome can be used as a reference for future population genomic studies. Understanding genomic variation among different golden mussel populations may unveil the routes of dispersion in invaded areas and support better control policies. Moreover, resequencing regions of interest will be necessary since mutations in target regions could render the biotechnology-based solution ineffective and require further

customization to answer to sequence changes. Lastly, structural variants have been detected in other mollusk species. In those species, a pattern of presence absence variation (PAV) has been reported, in which some genes are present only in some individuals of the population [56]. The chromosome-level genome may be used as a reference for studies resequencing multiple golden mussel individuals to check whether the species is also under the PAV and, if so, which parts of the genome are more or less conserved between individuals.

Discussion

The new reference genome reported in this study has shown better contiguity, completeness and accuracy metrics compared to the Illumina-based assembly, meaning that it is a more complete and reliable resource of information for the study of the golden mussel. Previous studies have shown that highly fragmented draft genomes can contain errors even in coding regions, endangering experimental and *in silico* studies that use its sequences as a reference. For instance, Korlach et al. have shown that the draft genome of two avian species had a series of misassemblies that generated issues (e.g. missing sequences and base call errors) in coding sequences and/or its flanking regions, and those issues could be solved after a new assembly based on PacBio long reads [57]. The high quality reference genome reported in this study increases the accuracy and completeness of genes of interest for the study of the golden mussel, supporting both fundamental and applied research on this invasive species. In addition to that, the high contiguity of the assembly opens the door to comparative studies at a chromosome scale, shedding light on the evolution of the golden mussel genome.

Analysis of gene family evolution in the golden mussel has shown two “Receptor-type protein tyrosine phosphatases” (RPTPs) families expanding at a significant rate. RPTPs counterbalance the phosphorylation promoted by kinases, which is an important signaling information that is involved in many different biological processes, such as

neuronal, immune and metabolic [58]. Other expanding families were annotated as either "Cadherin EGF LAG seven-pass G-type receptor" or “Protocadherin-like protein”. Proteins from the cadherin family are important for cell-cell adhesion. A previous study on the zebra mussel (*Dreissena polymorpha*) has shown that moderate thermal stress induces several genes involved in cellular adhesion, amongst them a cadherin protein (N-cadherin) [37]. Another study on the green mussel (*Perna viridis*) has shown that cadherins play a key role as foot proteins [59]. Finally, significant expansion was associated with a family of metalloproteases. In a study on *Crassostrea gigas*, it was shown that several metalloproteinase genes were upregulated in response to an infection with the herpes virus OsHV-1 [60]. The study argued that those proteinases may act to degrade viral or host-damaged proteins.

The DMRT gene family is known for its role in sex determination and differentiation and it has been proposed as a target for biotechnological population control strategies in the malaria mosquito [8]. Using the chromosome-level genome assembled in this study, we have done the first genome-wide characterization of the DMRT gene family in the golden mussel and we were able to identify DMRT1L, DMRT2, DMRT3 and DMRT4/5 orthologs. DMRT2/DMRT11E genes show varying functions. In mice, DMRT2 is involved in axial skeleton development, while in zebrafish DMRT2a/2b play roles in left-right patterning [61]. However, in arthropoda DMRT11E has shown to play a role in sex differentiation. Knockdown of *Drosophila* DMRT11E causes sperm malformation [62], while DMRT11E is required for proper oogenesis in the silkworm *Bombyx mori* [63]. DMRT2 function in mollusks is still unclear, however studies of expression profiles suggest its participation in spermatogenic cell differentiation in the pearl oyster *Pinctada fucata* and in the scallop *Chlamys nobilis [64,65]*.

In mammals, DMRT3 plays a role in neurogenesis, with related mutations associated with locomotion problems in horses and spinal circuit malfunction in mice [66]. DMRT3 have shown high expression in testis in some mammalian and fish species, suggesting

a potential role in testicular development [67,68]. DMRT4/5/99B genes have a well-conserved function in different species being mainly involved in neurogenesis. Mutations of DMRT4 and DMRT5 in vertebrates cause neuronal abnormalities [69–71], just like mutations do in the DMRT99B in arthropods [72,73]. As far as we know, no mutation study has been carried out on mollusks to support the DMRT5 function, although its tissue-wide distribution and expression indicates it may play a role in early embryonic development and various biological processes in *C. nobilis [64]*.

Dsx (arthropods), MAB-3 (nematodes) and DMRT1 (vertebrates) genes are members of the DMRT family historically associated with sex determination and differentiation roles [15,74,75]. Although sharing the same function, there is some debate as to whether those genes share a common gene ancestor. Based on phylogenetic and synteny analyses, Mawaribuchi et al conclude that those three genes form separate clusters and therefore might have emerged independently in each clade [76]. The phylogenetic analysis for the DMRT family in our study is in agreement with that, with the addition of a cluster of sex differentiation genes specific to mollusks named DMRT1L. Those genes consistently share a pattern of male-biased expression in the gonads in many other mollusk species [19–21,77] and a recent study has confirmed that knockdown of the DMRT1L in *Crassostrea gigas* cause male gonads to fail to differentiate [78]. If DMRT1L knockdown in the golden mussel shows the same consequences, it can be a strong target for population control strategies of this invasive species.

Data availability

The genome sequence is available in the NCBI under accession GCA_944474755.1, while contigs representing the alternative haplotype are available as GCA_944589985.1. Raw data accessions are summarized in Table 5.

Table 5. Accession numbers of raw sequencing data used for the genome assembly project.

Library	Accession(s)

Pacific Biosciences SEQUEL II (HiFi)	ERR9713989-91, ERR9713993
10X Genomics Illumina	ERR9503462-65
Hi-C Illumina	ERR9503466

List of abbreviations

DMRT - Doublesex and Mab-3 related transcription factor

GO - gene ontology

GRIT - Genome Reference Informatics Team

LINE - Long interspersed nuclear elements

lncRNA - long non-coding RNA

LTR - Long terminal repeats

misc_RNA - miscellaneous RNA

PAV - presence-absence variation

RPTP - Receptor-type protein tyrosine phosphatase

rRNA - ribosomal RNA

scaRNA - small Cajal body-specific RNA

SINE - Short Interspersed nuclear element

snRNA - small nuclear RNA

snoRNA - small nucleolar RNA

tRNA - transfer RNA

WSI - Wellcome Sanger Institute

VGP - Vertebrates Genome Project

Competing interests

The authors declare that they have no competing interests.

Funding

This work was financed by the Brazilian National Electric Energy Agency ANEEL R&D program (grant PD-10381-0419/2019). We also thank CTG Brasil, Tijoá Energia and Spic Brasil for funding this project through the ANEEL R&D Program. João Gabriel R. N. Ferreira and Fábio Sendim were recipients of Ph.D. fellowships and Yasmin R. da Cunha was a recipient of a Master's fellowship from CAPES, a federal government agency of the Brazilian Ministry of Education, which supports graduate students and faculty. Genome sequencing and assembly was provided by the Wellcome Sanger Institute Tree of Life Programme in collaboration with the Bio Bureau Biotechnology company.

Author's contributions

J.A.A., M.F.R. and M.U-S. designed the project. M.U-S. and J.G.R.N.F. planned the bioinformatics analyses. J.G.R.N.F. performed the bioinformatics and data analyses. J.G.R.N.F. wrote the first version of the manuscript. D.L.A.S.A, F.S. and Y.R.C. worked on the collection of golden mussel specimens and tissue dissection. All authors contributed to writing and approved the final manuscript.

Ethics/compliance issues

The materials that have contributed to this Research Article have been supplied by a Darwin Tree of Life Partner. The submission of materials by a Darwin Tree of Life Partner is subject to the Darwin Tree of Life Project Sampling Code of Practice (https://www.darwintreeoflife.org/wp-content/uploads/2021/03/DToL-Sampling-Code-of-Practice.pdf). By agreeing with and signing up to the Sampling Code of Practice, the Darwin Tree of Life Partner agrees they will meet the legal and ethical requirements and standards set out within this document in respect of all samples acquired for, and supplied to, the Darwin Tree of Life Project. Each transfer of samples is further undertaken according to a Research Collaboration Agreement or Material Transfer Agreement entered into by the Darwin Tree of Life Partner, Genome Research Limited

(operating as the Wellcome Sanger Institute), and in some circumstances other Darwin Tree of Life collaborators.

References

1. CBEIH. Centro de Bioengenharia de Espécies Invasoras de Hidrelétricas. https://base.cbeih.org/index.php Accessed 2022 Dec 14.

2. Boltovskoy D, Karatayev A, Burlakova L, Cataldo D, Karatayev V, Sylvester F, et al.. Significant ecosystem-wide effects of the swiftly spreading invasive freshwater bivalve Limnoperna fortunei. *Hydrobiologia*. 636:271–842009;

3. Cataldo D, O´ Farrell I, Paolucci E, Sylvester F, Boltovskoy D. Impact of the invasive golden mussel (Limnoperna fortunei) on phytoplankton and nutrient cycling. *Aquat Invasions*. Regional Euro-Asian Biological Invasions Centre Oy (REABIC); 7:91–1002012;

4. De Nys R, Guenther J. 8 - The impact and control of biofouling in marine finfish aquaculture. In: Hellio C, Yebra D, editors. *Advances in Marine Antifouling Coatings and Technologies*. Woodhead Publishing; p. 177–221.

5. Prescott TH, Claudi R, Prescott KL. Impact of dreissenid mussels on the infrastructure of dams and hydroelectric power plants. *Quagga and zebra mussels: biology, impacts, and control*. CRC Press Boca Raton, FL; :315–292013;

6. Rebelo MF, Afonso LF, Americo JA, da Silva L, Neto JLB, Dondero F, et al.. A sustainable synthetic biology approach for the control of the invasive golden mussel (Limnoperna fortunei). PeerJ Preprints; 2018 Sep. Report No.: e27164v3.

7. Hammond A, Galizi R, Kyrou K, Simoni A, Siniscalchi C, Katsanos D, et al.. A CRISPR-Cas9 gene drive system targeting female reproduction in the malaria mosquito vector Anopheles gambiae. *Nat Biotechnol*. 34:78–832016;

8. Kyrou K, Hammond AM, Galizi R, Kranjc N, Burt A, Beaghton AK, et al.. A CRISPR–Cas9 gene drive targeting doublesex causes complete population suppression in caged Anopheles gambiae mosquitoes. *Nat Biotechnol*. Nature Publishing Group; 36:1062–62018;

9. Kim S, Namekawa SH, Niswander LM, Ward JO, Lee JT, Bardwell VJ, et al.. A mammal-specific Doublesex homolog associates with male sex chromatin and is required for male meiosis. *PLoS Genet*. journals.plos.org; 3:e622007;

10. Saúde L, Lourenço R, Gonçalves A, Palmeirim I. terra is a left–right asymmetry gene required for left–right synchronization of the segmentation clock. *Nat Cell Biol*. Nature Publishing Group; 7:918–202005;

11. Yoshizawa A, Nakahara Y, Izawa T, Ishitani T, Tsutsumi M, Kuroiwa A, et al.. Zebrafish Dmrta2 regulates neurogenesis in the telencephalon. *Genes Cells*. Wiley Online Library; 16:1097–1092011;

12. Burtis KC, Baker BS. Drosophila doublesex gene controls somatic sexual differentiation by producing alternatively spliced mRNAs encoding related sex-specific polypeptides. *Cell*. 56:997–10101989;

13. Scali C, Catteruccia F, Li Q, Crisanti A. Identification of sex-specific transcripts of the Anopheles gambiae doublesex gene. *J Exp Biol*. 208:3701–92005;

14. Shukla JN, Palli SR. Doublesex target genes in the red flour beetle, Tribolium castaneum. *Sci Rep*. 2:9482012;

15. Shen MM, Hodgkin J. mab-3, a gene required for sex-specific yolk protein expression and a male-specific lineage in C. elegans. *Cell*. Elsevier; 54:1019–311988;

16. Zhou L, Ma X, Zhu N, Zou Q, Guo K, Bai L, et al.. The role of mab-3 in spermatogenesis and ontogenesis of pinewood nematode, Bursaphelenchus xylophilus. *Pest Manag Sci*. Wiley; 77:138–472021;

17. Raymond CS, Murphy MW, O'Sullivan MG, Bardwell VJ, Zarkower D. Dmrt1, a gene related to worm and fly sexual regulators, is required for mammalian testis differentiation. *Genes Dev*. 14:2587–952000;

18. Yoshimoto S, Ito M. A ZZ/ZW-type sex determination in Xenopus laevis. *FEBS J*. 278:1020–62011;

19. Zhang N, Xu F, Guo X. Genomic analysis of the Pacific oyster (Crassostrea gigas) reveals possible conservation of vertebrate sex determination in a mollusc. *G3* . 4:2207–172014;

20. Li R, Zhang L, Li W, Zhang Y, Li Y, Zhang M, et al.. FOXL2 and DMRT1L Are Yin and Yang Genes for Determining Timing of Sex Differentiation in the Bivalve Mollusk Patinopecten yessoensis. *Front Physiol*. 9:11662018;

21. Evensen KG, Robinson WE, Krick K, Murray HM, Poynton HC. Comparative phylotranscriptomics reveals putative sex differentiating genes across eight diverse bivalve species. *Comp Biochem Physiol Part D Genomics Proteomics*. Elsevier; 41:1009522022;

22. McCartney MA, Mallez S, Gohl DM. Genome projects in invasion biology. *Conserv Genet*. 20:1201–222019;

23. Uliano-Silva M, Dondero F, Dan Otto T, Costa I, Lima NCB, Americo JA, et al.. A hybrid-hierarchical genome assembly strategy to sequence the invasive golden mussel, Limnoperna fortunei. *Gigascience*. academic.oup.com; 2018; doi: 10.1093/gigascience/gix128.

24. Ieyama H. Chromosomes and nuclear DNA contents of Limnoperna in Japan (Bivalvia: Mytilidae). *Venus*. jstage.jst.go.jp; 1996;

25. Reis AC, Amaral D, Americo JA, Rebelo MF, de Sousa SM. Cytogenetic characterization of the golden mussel (Limnoperna fortunei) reveals the absence of sex heteromorphic chromosomes. *Annals of the brazilian academy of sciences*.

26. Cheng H, Concepcion GT, Feng X, Zhang H, Li H. Haplotype-resolved de novo assembly using phased assembly graphs with hifiasm. *Nat Methods*. nature.com; 18:170–52021;

27. Genomics 10x. longranger: 10x Genomics Linked-Read Alignment, Variant Calling, Phasing, and Structural Variant Calling. Github;

28. Garrison E, Marth G. Haplotype-based variant detection from short-read sequencing. arXiv [q-bio.GN].

29. Zhou C, McCarthy SA, Durbin R. YaHS: yet another Hi-C scaffolding tool. bioRxiv.

30. Howe K, Chow W, Collins J, Pelan S, Pointon D-L, Sims Y, et al.. Significantly improving the quality of genome assemblies through curation. *Gigascience*. 2021; doi: 10.1093/gigascience/giaa153.

31. Cunningham F, Allen JE, Allen J, Alvarez-Jarreta J, Amode MR, Armean IM, et al.. Ensembl 2022. *Nucleic Acids Res*. Oxford Academic; 50:D988–952021;

32. Uliano-Silva M. MitoHiFi: Find, circularise and annotate mitogenome from PacBio assemblies. Github;

33. Manni M, Berkeley MR, Seppey M, Simão FA, Zdobnov EM. BUSCO Update: Novel and Streamlined Workflows along with Broader and Deeper Phylogenetic Coverage for Scoring of Eukaryotic, Prokaryotic, and Viral Genomes. *Mol Biol Evol*. 38:4647–542021;

34. Rhie A, Walenz BP, Koren S, Phillippy AM. Merqury: reference-free quality, completeness, and phasing assessment for genome assemblies. *Genome Biol*. genomebiology.biomedcentral.com; 21:2452020;

35. Rhie A, McCarthy SA, Fedrigo O, Damas J, Formenti G, Koren S, et al.. Towards complete and error-free genome assemblies of all vertebrate species. *Nature*. nature.com; 592:737–462021;

36. Baril T, Imrie RM, Hayward A. Earl Grey: a fully automated user-friendly transposable element annotation and analysis pipeline.

37. McCartney MA, Auch B, Kono T, Mallez S, Zhang Y, Obille A, et al.. The genome of the zebra mussel, Dreissena polymorpha: a resource for comparative genomics, invasion genetics, and biocontrol. *G3* . 2022; doi: 10.1093/g3journal/jkab423.

38. Calcino AD, de Oliveira AL, Simakov O, Schwaha T, Zieger E, Wollesen T, et al.. The quagga mussel genome and the evolution of freshwater tolerance. *DNA Res*. Oxford Academic; 26:411–222019;

39. Emms DM, Kelly S. OrthoFinder: phylogenetic orthology inference for comparative genomics. *Genome Biol*. Springer; 20:2382019;

40. Camacho C, Coulouris G, Avagyan V, Ma N, Papadopoulos J, Bealer K, et al.. BLAST+: architecture and applications. *BMC Bioinformatics*. Springer; 10:4212009;

41. Buchfink B, Xie C, Huson DH. Fast and sensitive protein alignment using DIAMOND. *Nat Methods*. nature.com; 12:59–602015;

42. Cantalapiedra CP, Hernández-Plaza A, Letunic I, Bork P, Huerta-Cepas J. eggNOG-mapper v2: Functional Annotation, Orthology Assignments, and Domain Prediction at the Metagenomic Scale. *Mol Biol Evol*. academic.oup.com; 38:5825–92021;

43. Finn RD, Clements J, Eddy SR. HMMER web server: interactive sequence similarity searching. *Nucleic Acids Res*. Oxford University Press; 39:W29–372011;

44. De Bie T, Cristianini N, Demuth JP, Hahn MW. CAFE: a computational tool for the study of gene family evolution. *Bioinformatics*. academic.oup.com; 22:1269–712006;

45. Mendes FK, Vanderpool D, Fulton B, Hahn MW. CAFE 5 models variation in

evolutionary rates among gene families. *Bioinformatics*. Oxford Academic; 36:5516–82020;

46. Jones P, Binns D, Chang H-Y, Fraser M, Li W, McAnulla C, et al.. InterProScan 5: genome-scale protein function classification. *Bioinformatics*. academic.oup.com; 30:1236–402014;

47. Klopfenstein DV, Zhang L, Pedersen BS, Ramírez F, Warwick Vesztrocy A, Naldi A, et al.. GOATOOLS: A Python library for Gene Ontology analyses. *Sci Rep*. nature.com; 8:108722018;

48. Thompson JD, Gibson TJ, Higgins DG. Multiple sequence alignment using ClustalW and ClustalX. *Curr Protoc Bioinformatics*. Wiley Online Library; Chapter 2:Unit 2.32002;

49. Capella-Gutiérrez S, Silla-Martínez JM, Gabaldón T. trimAl: a tool for automated alignment trimming in large-scale phylogenetic analyses. *Bioinformatics*. academic.oup.com; 25:1972–32009;

50. Darriba D, Posada D, Kozlov AM, Stamatakis A, Morel B, Flouri T. ModelTest-NG: A New and Scalable Tool for the Selection of DNA and Protein Evolutionary Models. *Mol Biol Evol*. academic.oup.com; 37:291–42020;

51. Huelsenbeck JP, Ronquist F. MRBAYES: Bayesian inference of phylogenetic trees. *Bioinformatics*. 17:754–52001;

52. Ronquist F, Teslenko M, van der Mark P, Ayres DL, Darling A, Höhna S, et al.. MrBayes 3.2: efficient Bayesian phylogenetic inference and model choice across a large model space. *Syst Biol*. academic.oup.com; 61:539–422012;

53. Letunic I, Bork P. Interactive Tree Of Life (iTOL): an online tool for phylogenetic tree display and annotation. *Bioinformatics*. academic.oup.com; 23:127–82007;

54. Letunic I, Bork P. Interactive Tree Of Life (iTOL) v5: an online tool for phylogenetic tree display and annotation. *Nucleic Acids Res*. academic.oup.com; 49:W293–62021;

55. Afonso LF, Americo JA, Soares-Souza GB, Torres ALQ, Wajsenzon IJR, de Freitas Rebelo M. Gonad transcriptome of golden mussel Limnoperna fortunei reveals potential sex differentiation genes. bioRxiv.

56. Calcino AD, Kenny NJ, Gerdol M. Single individual structural variant detection uncovers widespread hemizygosity in molluscs. *Philos Trans R Soc Lond B Biol Sci*. 376:202001532021;

57. Korlach J, Gedman G, Kingan SB, Chin C-S, Howard JT, Audet J-N, et al.. De novo PacBio long-read and phased avian genome assemblies correct and add to reference genes generated with intermediate and short reads. *Gigascience*. 6:1–162017;

58. Senis YA, Barr AJ. Targeting Receptor-Type Protein Tyrosine Phosphatases with Biotherapeutics: Is Outside-in Better than Inside-Out? *Molecules*. 2018; doi: 10.3390/molecules23030569.

59. Inoue K, Yoshioka Y, Tanaka H, Kinjo A, Sassa M, Ueda I, et al.. Genomics and Transcriptomics of the green mussel explain the durability of its byssus. *Sci Rep*. 11:59922021;

60. He Y, Jouaux A, Ford SE, Lelong C, Sourdaine P, Mathieu M, et al.. Transcriptome

analysis reveals strong and complex antiviral response in a mollusc. *Fish Shellfish Immunol*. 46:131–442015;

61. Lourenço R, Lopes SS, Saúde L. Left-right function of dmrt2 genes is not conserved between zebrafish and mouse. *PLoS One*. 5:e144382010;

62. Yu J, Wu H, Wen Y, Liu Y, Zhou T, Ni B, et al.. Identification of seven genes essential for male fertility through a genome-wide association study of non-obstructive azoospermia and RNA interference-mediated large-scale functional screening in Drosophila. *Hum Mol Genet*. 24:1493–5032015;

63. Kasahara R, Yuzawa T, Fujii T, Aoki F, Suzuki MG. dmrt11E ortholog is a crucial factor for oogenesis of the domesticated silkworm, Bombyx mori. *Insect Biochem Mol Biol*. 129:1035172021;

64. Shi Y, Wang Q, He M. Molecular identification of dmrt2 and dmrt5 and effect of sex steroids on their expressions in Chlamys nobilis. *Aquaculture*. 426-427:21–302014;

65. Yu F-F, Wang M-F, Zhou L, Gui J-F, Yu X-Y. Molecular Cloning and Expression Characterization of Dmrt2 in Akoya Pearl Oysters, Pinctada martensii. *shre*. National Shellfisheries Association; 30:247–542011;

66. Andersson LS, Larhammar M, Memic F, Wootz H, Schwochow D, Rubin C-J, et al.. Mutations in DMRT3 affect locomotion in horses and spinal circuit function in mice. *Nature*. Nature Publishing Group; 488:642–62012;

67. Hong C-S, Park B-Y, Saint-Jeannet J-P. The function of Dmrt genes in vertebrate development: it is not just about sex. *Dev Biol*. 310:1–92007;

68. Yamaguchi A, Lee KH, Fujimoto H, Kadomura K, Yasumoto S, Matsuyama M. Expression of the DMRT gene and its roles in early gonadal development of the Japanese pufferfish Takifugu rubripes. *Comp Biochem Physiol Part D Genomics Proteomics*. 1:59–682006;

69. Ratié L, Desmaris E, García-Moreno F, Hoerder-Suabedissen A, Kelman A, Theil T, et al.. Loss of Dmrt5 Affects the Formation of the Subplate and Early Corticogenesis. *Cereb Cortex*. 30:3296–3122020;

70. Graf M, Teo Qi-Wen E-R, Sarusie MV, Rajaei F, Winkler C. Dmrt5 controls corticotrope and gonadotrope differentiation in the zebrafish pituitary. *Mol Endocrinol*. 29:187–992015;

71. Urquhart JE, Beaman G, Byers H, Roberts NA, Chervinsky E, O'Sullivan J, et al.. DMRTA2 (DMRT5) is mutated in a novel cortical brain malformation. *Clin Genet*. 89:724–72016;

72. Kasahara R, Aoki F, Suzuki MG. Deficiency in dmrt99B ortholog causes behavioral abnormalities in the silkworm, Bombyx mori. *Appl Entomol Zool* . 53:381–932018;

73. Zwarts L, Vanden Broeck L, Cappuyns E, Ayroles JF, Magwire MM, Vulsteke V, et al.. The genetic basis of natural variation in mushroom body size in Drosophila melanogaster. *Nat Commun*. Nature Publishing Group; 6:1–112015;

74. Huang S, Ye L, Chen H. Sex determination and maintenance: the role of DMRT1 and FOXL2. *Asian J Androl*. 19:619–242017;

75. Erdman SE, Burtis KC. The Drosophila doublesex proteins share a novel zinc

finger related DNA binding domain. *EMBO J*. 12:527–351993;

76. Mawaribuchi S, Ito Y, Ito M. Independent evolution for sex determination and differentiation in the DMRT family in animals. *Biol Open*. 2019; doi: 10.1242/bio.041962.

77. Li J, Zhou Y, Zhou Z, Lin C, Wei J, Qin Y, et al.. Comparative transcriptome analysis of three gonadal development stages reveals potential genes involved in gametogenesis of the fluted giant clam (Tridacna squamosa). *BMC Genomics*. 21:8722020;

78. Sun D, Yu H, Li Q. Examination of the roles of Foxl2 and Dmrt1 in sex differentiation and gonadal development of oysters by using RNA interference. *Aquaculture*. 548:7377322022;

Additional files

Additional file 1. Significantly expanded (p<0.05) golden mussel gene families according to CAFE analysis.

Additional file 2. Significantly contracted (p<0.05) golden mussel gene families according to CAFE analysis

Figure 1

Click here to access/download;Figure;Figure1.png

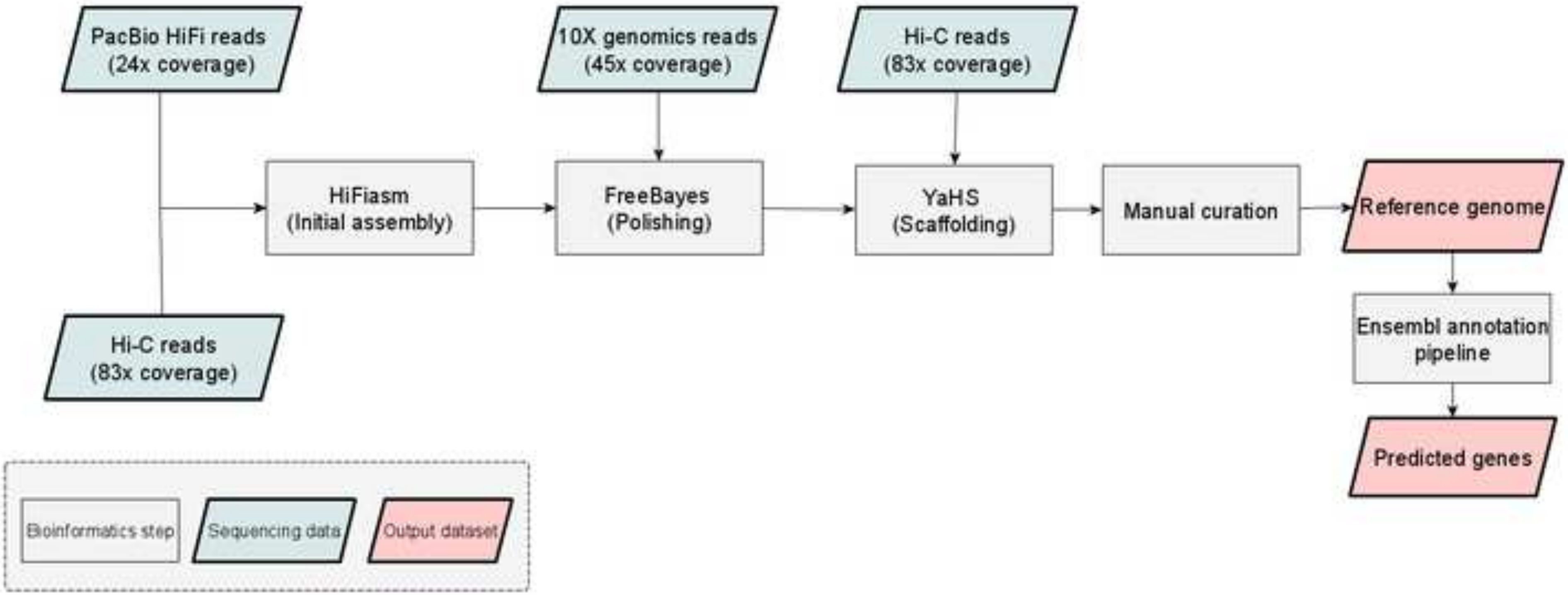

Figure 2 Click here to access/download;Figure;Figure2.png

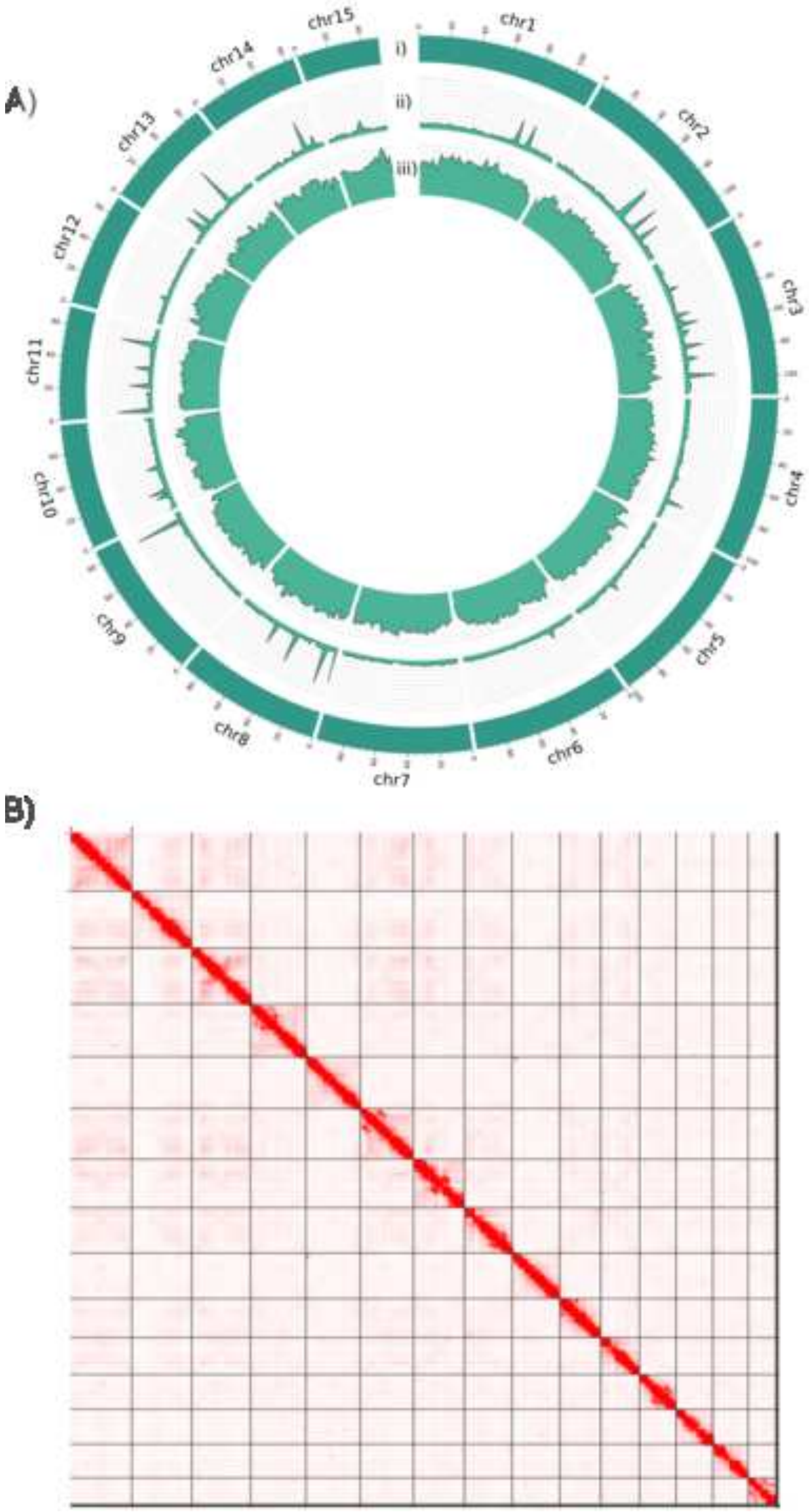

Figure 3

Click here to access/download;Figure;Figure3.png

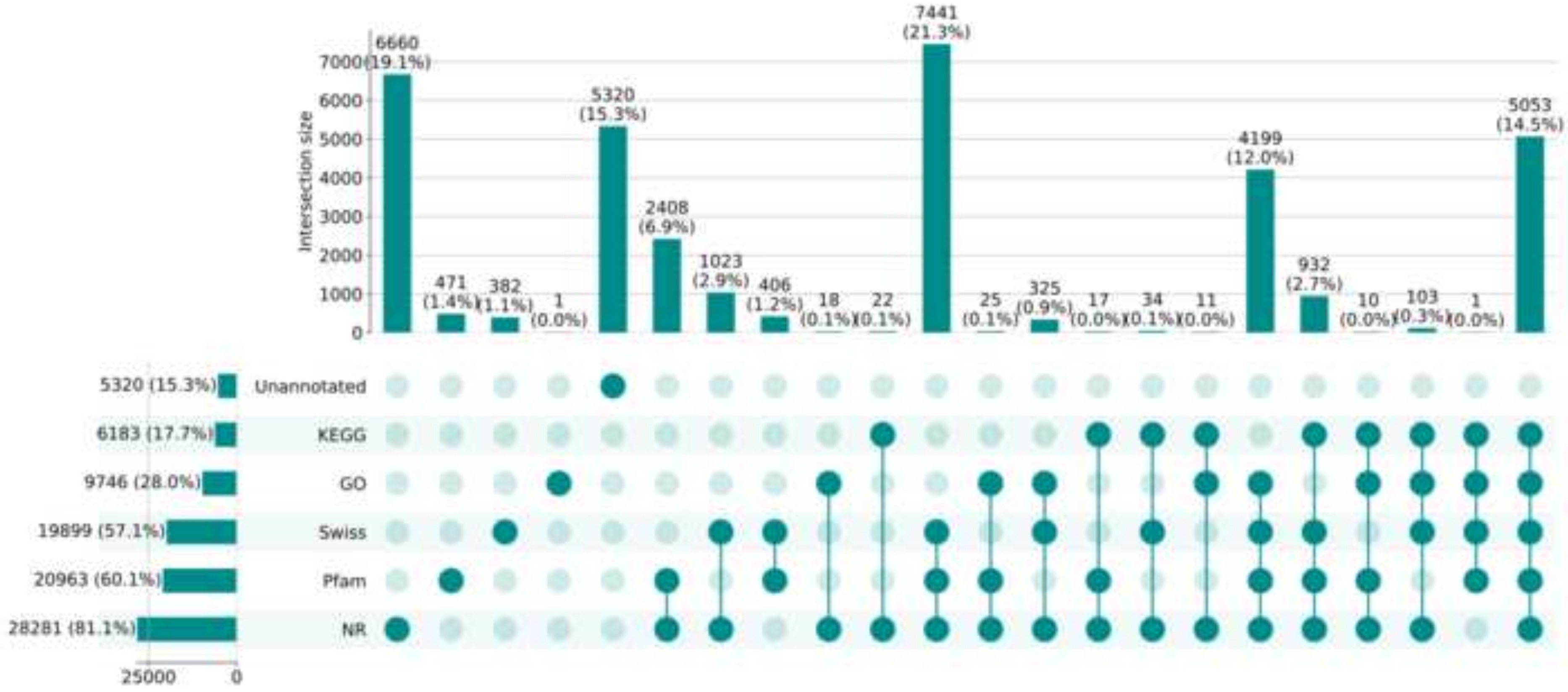

Figure 4

Click here to access/download;Figure;Figure4.png

A)

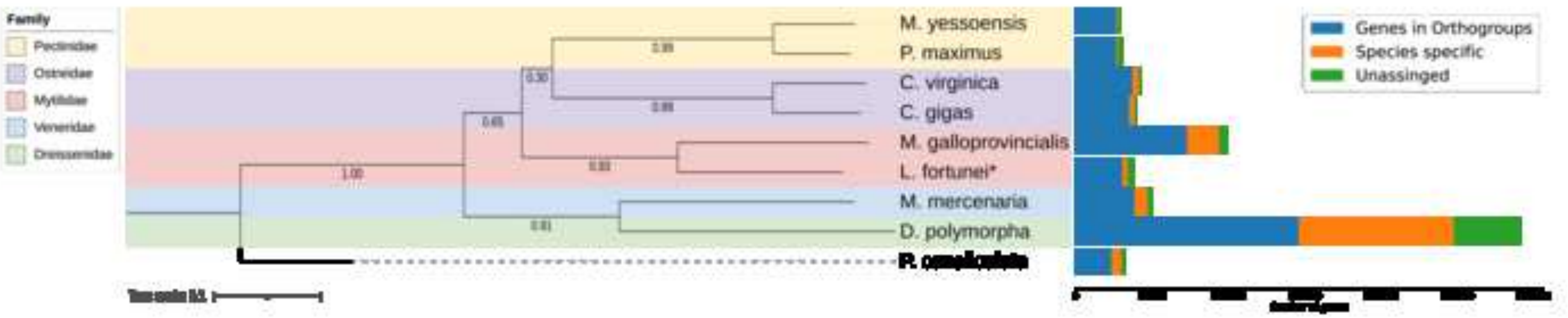

B)

	C. gigas	C. virginica	D. polymorpha	L. fortunei	M. galloprovincialis	M. mercenaria	M. yessoensis	P. canaliculata	P. maximus
C. gigas									
C. virginica	14545								
D. polymorpha	11144	10821							
L. fortunei	11889	11547	11669						
M. galloprovincialis	11738	11359	12167	14411					
M. mercenaria	11004	10661	13214	11192	11253				
M. yessoensis	11802	11489	11251	11846	11667	11047			
P. canaliculata	10183	9963	10113	10274	9926	9958	10350		
P. maximus	11892	11546	11306	11929	11739	11142	14548	10346	

Figure 5

Click here to access/download;Figure;Figure5.png

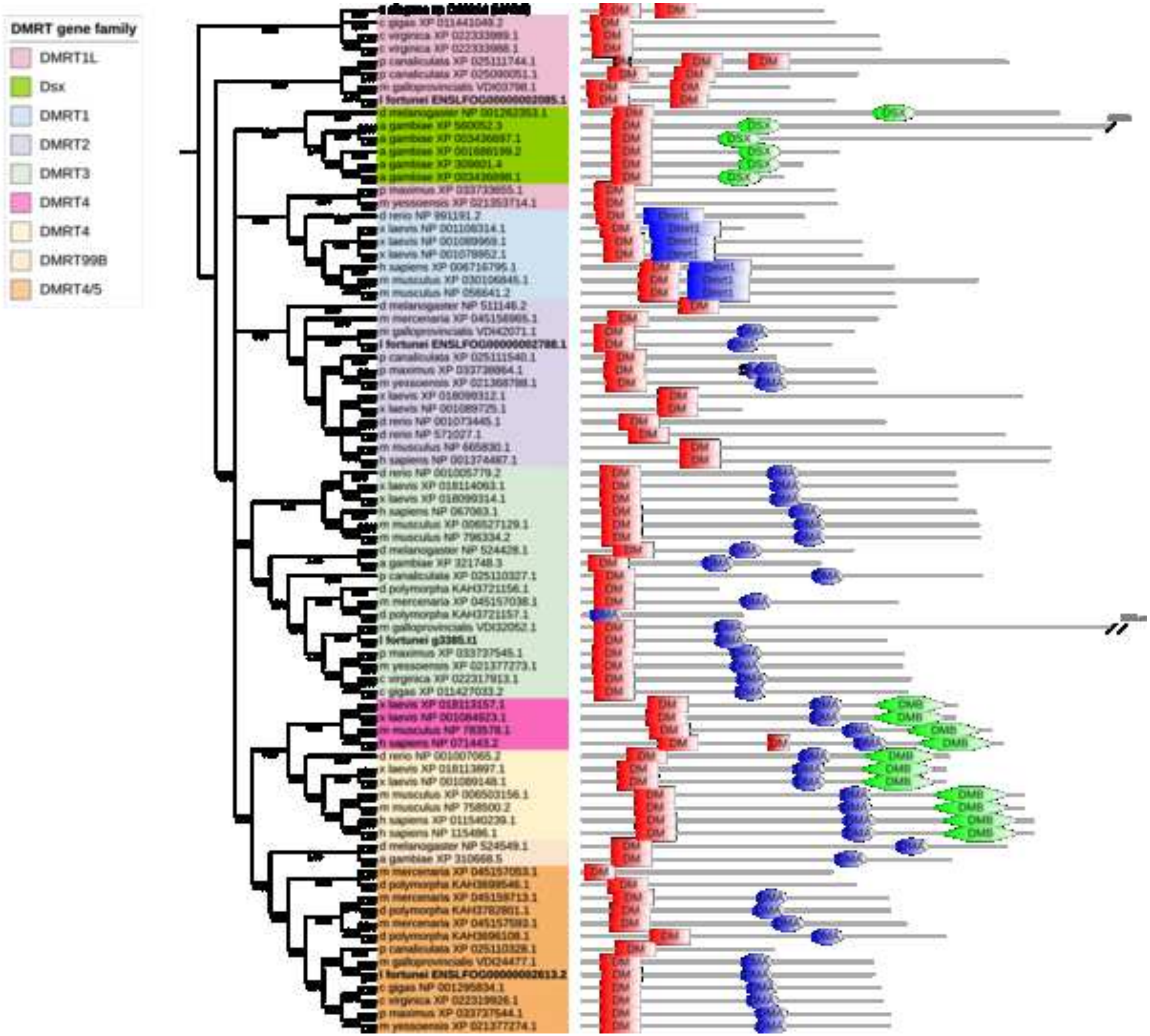

Additional file 1

Click here to access/download
Supplementary Material
Gamma_asr.Lfor_Sign.expanded.tre

Additional file 2

Click here to access/download
Supplementary Material
Gamma_asr.Lfor_Sign.contracted.tre

Click here to access/download
Supplementary Material
Ferreira_et_al_2022_supplementary.docx

Cover letter Click here to access/download;Personal Cover;cover_letter.docx

Dear Editor-in-Chief,

We are enclosing herewith a manuscript by João G. R. N. Ferreira, Juliana A. Americo, Danielle L. A. S. do Amaral, Fábio Sendim, Yasmin R. da Cunha, The Darwin Tree of Life Project Consortium, Marcela Uliano-Silva and Mauro de F. Rebelo entitled “A chromosome-level genome supports genome-wide investigation of the DMRT gene family in the golden mussel (*Limnoperna fortunei*)” submitted to GigaScience for possible evaluation. The type of submitted manuscript is Data Note.

The golden mussel is an Asian freshwater bivalve that was introduced in South America almost 30 years ago and since then has spread across the continent, causing both economic and environmental impacts. Traditional control strategies haven’t been able to stop dispersal of the golden mussel, and our group proposes a biotechnology solution based on *gene drive*. A *gene drive* solution for population control of the malaria mosquito has been showing promising results targeting genes involved in sex differentiation, however little is known about sex differentiation in the golden mussel. In addition to that, the current reference genome is highly fragmented, hindering its applications not only for the planning of genome editing experiments but also for the study of the molecular biology of the species as a whole.

In this study, we have developed a new, high-quality reference genome for the golden mussel. The assembly was achieved using a combination of PacBio HiFi, Hi-C and 10X sequencing data, and 99.4% of its sequence is distributed over the 15 largest scaffolds, that putatively represent the 15 chromosomes of the species. Based on the chromosome-level genome we detected expanded golden mussel gene families with roles in structural and defensive mechanisms. We have also done a genome-wide characterization of the DMRT gene family, identifying four DMRT genes in the golden mussel. One of those genes (DMRT1L) is assumed to play a role in sex determination and differentiation and therefore represents a potential target for biotechnology based control strategies. The new genome and the findings of this study are expected to support both basic and applied research on this invasive species.

The authors of this study declare no competing interests and all authors have approved the manuscript for submission. The content of the manuscript has not been published or submitted for publication elsewhere, although a preprint in a Genome Announcement format (i.e., containing none of the downstream analyzes) has been published at bioRxiv (doi: https://doi.org/10.1101/2022.09.29.509984)

Sincerely,

Juliana Alves Americo

Universidade Federal do Rio de Janeiro, Instituto de Biofísica Carlos Chagas Filho, Centro de Ciências da Saúde. Av. Carlos Chagas Filho, 373. Bloco G - Sala G2-050 - Cidade Universitária CEP: 21941-902 - Rio de Janeiro - RJ
