# Supplementary material for: A chromosome-level assembly supports genome-wide investigation of the DMRT gene family in the golden mussel (Limnoperna fortunei)
Source: Gigascience. 2023 Sep 30;12:giad072. doi: 10.1093/gigascience/giad072 (PMC10541798; doi:10.1093/gigascience/giad072)
Supplement: giad072_GIGA-D-22-00343_Revision_1 [file giad072_giga-d-22-00343_revision_1.pdf]

GigaScience

A chromosome-level assembly supports genome-wide investigation of the DMRT gene family in the golden mussel (Limnoperna fortunei)

--Manuscript Draft--

**Manuscript Number:**	GIGA-D-22-00343R1
**Full Title:**	A chromosome-level assembly supports genome-wide investigation of the DMRT gene family in the golden mussel (Limnoperna fortunei)
**Article Type:**	Data Note
**Funding Information:**	Wellcome Sanger Core Award (220540/Z/20/A): _ Tree of Life Programme<br>Coordenação de Aperfeiçoamento de Pessoal de Nível Superior: Mr João Gabriel Rodinho Nunes Ferreira, Mr Fábio Sendim, Ms. Yasmin R. da Cunha<br>Agência Nacional de Energia Elétrica (PD-10381-0419/2019): Not applicable
**Abstract:**	Background<br>The golden mussel (Limnoperna fortunei) is a highly invasive species that causes environmental and socioeconomic losses in invaded areas. Reference genomes have proven to be a valuable resource for studying the biology of invasive species. While the current golden mussel genome has been useful for identifying new genes, its high fragmentation hinders some applications.<br>Findings<br>In this study, we provide the first chromosome-level reference genome for the golden mussel. The genome was built using PacBio HiFi, 10X and Hi-C sequencing data. The final assembly contains 99.4% of its total length assembled to the 15 chromosomes of the species and a scaffold N50 of 97.05 Mb. A total of 34 862 protein-coding genes were predicted, of which 84.7% were functionally annotated. A significant (6.48%) proportion of the genome was found to be in a hemizygous state. Using the new genome, we have performed a genome-wide characterization of the DMRT gene family, which has been proposed as a target for population control strategies in other species.<br>Conclusions<br>From the applied research perspective, a higher quality genome will support genome editing with the aim of developing biotechnology-based solutions to control invasion. From the basic research perspective, the new genome is a high-quality reference for molecular evolutionary studies of Mytilida and other Lophotrochozoa, and it may be used as a reference for future resequencing studies to assess genomic variation among different golden mussel populations, unveiling potential routes of dispersion and helping to establish better control policies.
**Corresponding Author:**	Juliana Alves Americo, Ph.D<br>Bio Bureau Biotecnologia<br>Rio de Janeiro, RJ BRAZIL
**Corresponding Author Secondary Information:**	
**Corresponding Author's Institution:**	Bio Bureau Biotecnologia
**Corresponding Author's Secondary Institution:**	
**First Author:**	João Gabriel Rodinho Nunes Ferreira, MSc
**First Author Secondary Information:**	
**Order of Authors:**	João Gabriel Rodinho Nunes Ferreira, MSc<br>Juliana Alves Americo, Ph.D<br>Danielle L. A. S. do Amaral, Ph.D

Powered by Editorial Manager® and ProduXion Manager® from Aries Systems Corporation

	Fábio Sendim, MSc
	Yasmin R. da Cunha, BSc
	Tree of Life Programme
	Mark Blaxter, PhD
	Marcela Uliano-Silva, Ph.D
	Mauro de Freitas Rebelo, PhD
**Order of Authors Secondary Information:**	

Response to Reviewers:

Dear Editor,
We are pleased to resubmit the revised manuscript titled "A chromosome-level assembly supports genome-wide investigation of the DMRT gene family in the golden mussel (Limnoperna fortunei)" by Ferreira et al. for further evaluation and potential publication in GigaScience.

We sincerely thank the reviewers for their valuable feedback, which greatly improved the quality and clarity of our research. We appreciate their dedication, expertise, and guidance throughout this process.

The reviewers have recognized a significant improvement in the genome assembly of Limnoperna fortunei compared to its original draft version. They also recognized its importance in understanding the mussel's biology, evolutionary relationships within bivalves, and developing control strategies for this invasive species. However, they identified areas where additional metrics, evidence, and methodological improvements were needed to better showcase the strengths of the new genome assembly.

To attend the reviewers requests we have included a section (“Overall genome characteristics”) with k-mer based estimates of genomic features (e.g. heterozygosity rate). The section “Gene family evolution” was removed due to the criticism of poor selection of species, which made it limited for assessing lineage specific gene family expansions/contractions. As suggested by one of the reviewers, we have included preliminary analysis of potential hemizygosity in the golden mussel genome in a new section (“Hemizygosity investigation”). We also made some minor grammatical corrections throughout to improve the manuscript and added a section listing all authors from the Tree of Life Programme, as suggested by Gigascience recommendations on Group Authorship. Lastly, we updated the Ethics/compliance statement to make clear that the samples were obtained following the Brazilian Biodiversity Law.

In the subsequent sections of this letter, we will systematically address each point raised by the reviewers, providing comprehensive responses and incorporating the requested improvements into the revised manuscript.
Thank you for the opportunity to revise and resubmit our manuscript. We are confident that the revised version represents a significant contribution to the field and now meets the high standards for publication set by GigaScience.
Sincerely,

Juliana A. Americo
On behalf of all authors

Reviewer 1

1. "may lead to incomplete or erroneous gene models." -> I think the authors may introduce here some metrics of the first draft assembly that suggest that this was, indeed, the case, for example by mentioning the relatively high rate of missing/fragmented gene models.

Answer: We thank the reviewer for his comments and suggestions. We have now updated the manuscript, and we present such metrics. The sentence presenting these

metrics is the following: ”Limitations of this draft genome constrain its applications in resequencing and comparative genomic studies and may lead to incomplete or erroneous gene models, as indicated by the high number of missing (10%) and fragmented (7%) BUSCO genes reported in the original paper [23]”.

2. "Recent advances in sequencing technologies and bioinformatics algorithms" -> I would also add something like "improved library preparation protocols", thinking about what makes both Hi-C and Hifi reads so useful.

Answer: Agreed and added to the text, thanks. The updated version of the sentence is: “Recent advances in library preparation protocols, sequencing technologies and bioinformatics algorithms have made the development of high quality reference genomes scalable and affordable.”

3. I have no methodological concerns, as the procedures described in the paper follow well-established standardized methodologies that have been employed for multiple other invertebrate genome projects in the context of large joint sequencing initiatives. I noticed that RNA-seq has not been mentioned anywhere as a source of data to complement gene annotation. I think the authors should mention whether they have exploited pre-existing data generated for example within the frame of the previous genome sequencing project.

Answer: We added the information about the datasets used to support annotation in the section “Gene prediction” and in the (new) Supplementary Table S2. The updated manuscript reads:

“The Ensembl rapid annotation pipeline [33] was used to predict genes. The prediction was supported by homologous proteins and pre-existing golden mussel RNA-seq data, including RNA-seq data from the draft genome project and from the same specimen (xbLimFort5) sequenced for the chromosome-level genome assembly (Supplementary Table S2)”.

4. "with two datasets: metazoa_odb10 and mollusca_odb10." -> while this makes sense, the Mollusca dataset is notoriously "bad" for such assessments at the present stage due to the low number of species used for its generation and a somewhat taxonomically-biased species composition, which often leads to nonsensical underestimates of completeness even for wonderful genome assemblies. Please consider removing the completeness data generated vs the molluscan dataset altogether, reporting only metazoan BUSCOs.

Answer: We agree with the reviewer, we have now removed the comparison to the molluscan dataset.

5. Gene prediction: how do the authors interpret the discrepancy between the number of gene models obtained with this pipeline and those reported for the previous version of the genome assembly? To what extent does the different quality of the assembly (i.e. lower fragmentation, much better chances to pick intact long genes, etc.) may explain this? To what extent does the annotation pipeline make a difference? I may also suggest to add (if useful) data about the mapping rate of RNA-seq datasets against the gene models of the two assemblies, as this would be another metric supporting a higher completeness of the present version of the assembly.

Answer: We have added the discussion about the difference in the number of genes and also compared the RNAseq mapping rate. The discussion now present in the manuscript is the following: “The number of predicted genes was significantly lower compared to the one reported for the draft genome (60 717) [23]. The draft nature of the previous genome could be a factor influencing prediction, because the genome was assembled in more than 20 000 scaffolds and the QV was low, and predictions can become fragmented and or truncated, overinflating the number of genes. This is in line with our new results, as even though the number of predicted genes for the chromosome-level assembly is lower (34 862), this prediction is much more complete as evidenced by a (i) 2.3-fold increase in the number of BUSCO genes found (Supplementary Table S4), (ii) decrease of duplicated and missing BUSCOs and a (iii) 1.8-fold increase in the number of RNAseq reads mapped (Supplementary Data Note 1

- Table 1).”

6. "10 000 000 MCMC generations" -> how was tree convergence evaluated?

Answer: Added to the manuscript: “Convergence was evaluated by checking if the standard deviation of split frequencies was <0.01”.

7. About the split gene of D. polymorpha, I would say that the interpretation provided by the authors is 100% correct. Rather than diverting the attention of the reader to this technical issue, I would simply suggest the authors to fish out the full length sequence from the transcriptome of the species (this should be available on TSA, if I remember correctly, without the need to de novo reassemble RNA-seq data) and replace the two partial gene models with the single correct full-length sequence. The whole process may be explained with a brief supplementary note.

Answer: Thanks for the suggestion. We have identified a transcript (GHIW01027633.1 from the GHIW00000000.1 transcriptome) that covers both genes and replaced the partial gene models (KAH3721156.1 and KAH3721157.1) by the complete one. The process is described in the Supplementary Data Note 3 and briefly mentioned in the main text: “Manual inspection to check for remaining isoforms and split gene models was also carried out (Supplementary Data Note 3).”

8. "raising the possibility that this domain may be absent in gastropods": yes, this is a possibility, but incorrect gene annotations are also quite frequent in bivalve genomes, so I would suggest the author to either name this alternative possibility or to spend a few minutes at checking whether assembled transcripts from RNA-seq data support annotated gene models lacking this domain or not.

Answer: We have searched a Pomacea canaliculata transcriptome (datadryad.org/stash/dataset/doi:10.5061/dryad.8n776) but we were not able to find a complete transcript for the DMRT4/5 gene. Given the uncertainty related to the P. canaliculata DMRT4/5 gene model and the fact that gastropods were not the focus of our DMRT analysis, we decided to remove all P. canaliculata sequences from the phylogenetic analysis. The manuscript has also been adjusted to remove all references to those sequences.

9. "they are actually isoforms from the same gene that OrthoFinder's script to remove isoform duplicates was not able to detect." -> this is another technicality that diverts the attention of the reader. It's ok to mention this, but perhaps this would better fit in a supplementary note.

Answer: We have removed that sentence from the manuscript. In the new version of the DMRT tree we have deleted the shortest isoform, which was XP_022333988.1. Therefore, only XP_022333989.1 was kept. The removal is described in Supplementary Data Note 3.

10. The authors made an interesting remark about structural variation in the very last part of their manuscript, suggesting that this reference may be used to study whether gene PAV is present in L. fortunei. In perspective, such studies may be also useful to understand the genomic factors underlying the great invasive potential of this species, since dispensable genes are clearly enriched in functions linked with immunity and survival in Mytilus. I guess the assembly strategy used by the authors produced a primary reference assembly, plus an alternate haplotype, as it happens in many other recent genome assembly approaches that exploit Hi-C data (this is also briefly mentioned in the supplementary data file availability statement). While an in depth analysis of this data goes beyond the scope of the manuscript, I think the authors could report a few additional metrics in the section where they describe the main features of the assembly. For example, while I think high heterozygosity should be expected from this species, I have not seen any estimate reported in the text. Moreover, since heterozygosity rates estimates are usually performed with k-mer based graphs, these tend to mix-up SNP signals with hemizygosity (k-mers derived from both will end up in the same leftmost peak. The authors can easily estimate the fraction of the genome that is apparently in a hemizygous state simply by analyzing the per-base coverage calculated over relatively large window sizes. The distribution obtained with such

calculations should (in case of widespread hemizygosity) also create two peaks, i.e. one corresponding to hemizygous regions not shared by the two haplotypes and one corresponding to core regions shared by both.

Answer: We have included a k-mer based estimate of the genome heterozygosity in a new section ("Overall genome characteristics"): "A k-mer based approach was used to estimate overall genome statistics from the PacBio HiFi data. Jellyfish [26] was used to calculate the frequencies of 31-bp long k-mers and GenomeScope [27] was used to build a model to infer genome characteristics. The genome was inferred to be diploid, with an estimated haploid size around 1.3 Gb (Supplementary Figure S1). The expected repeat content was 43% and a high heterozygosity rate was estimated (2.4%)."

We agree with the concern related to the hemizygosity and we have performed a preliminary identification of hemizygous regions. The analysis can be found in the new section: "Hemizygosity investigation". We have also updated other sections of the manuscript with information related to this new analysis. More specifically, we have added a sentence in the "Findings" topic of the "Abstract" section:
"A significant (6.48%) proportion of the genome was found to be in a hemizygous state". We have also added a paragraph in the Discussion section:
"Structural variation analysis showed that a significant (6.48%) proportion of the golden mussel genome was in a hemizygous state, i.e. the region is present in only one of the homologous chromosomes. The presence of hemizygous regions has already been seen in other molluscan species [44,55]. Compared to the eight molluscan species analyzed in Calcino et al [44], the golden mussel showed the second largest proportion of hemizygosity, only lower than the bivalve Scapharca (Anadara) broughtonii (6.69%). In another Mytilidae species (Mytilus galloprovincialis), it has been shown that the presence of hemizygous regions is correlated with the occurrence of gene presence/absence variation (PAV) [55], which, as discussed in the "Re-use potential" section, may have impacts on biotechnology-based control strategies. Future re-sequencing studies should allow us to move from a genome to a pangenome scenario and to check what set of golden mussel genes (if any) is under PAV."

11. Species choice: while I don't want to heavily criticize this, as I understand that this selection of species was enough to meet the needs of the authors for the present, work, I would also say that this was somewhat limited for assessing lineage-specific gene family expansions and contractions, so the discussion should be a little bit more balanced, acknowledging the limitations of such inferences due to narrow taxonomical sampling. For example, M. philippinarum, P. viridis and B. platifrons, if added, would have provided a much better overview on possible lineage-specific expansions and contractions, intended as those that are not shared by other major mytiloid lineages. In its present form, the expansion/contraction analysis does not really adds much to the manuscript, as it fails to really identify expansion (or contraction) events that are restricted to the Limnoperna lineage, i.e. not shared by other mussels. On the other hand, another way to exploit this analysis would have been to check for convergent traits shared by Dreissena and Limnoperna, as they are both highly invasive and freshwater, despite the fact they belong to largely divergent branches of the bivalve tree of life. In summary, my suggestion would be to remove the CAFE analysis altogether, or alternatively to maintain just a brief mention of the potential usefulness of similar analyses that should however be carried out with a broader taxonomical sampling in order to be informative.

Answer: The CAFE analysis (previous section "Gene family evolution") was removed from the manuscript as suggested by the reviewer.

12. Just brief comment on the phylogenetic analysis. The text is quite clear, as far as methodology is concerned, and the choice made by the authors are scientifically sound. Yet, I see a couple of minor issues that the authors should check, by looking at domain organization in Fig. 5. MSA is quite tricky whenever proteins displaying duplicated domains are present, as it is the case of some DMRTL1 sequences. The MSA was trimmed to remove noisy bits of sequence an unalignable stuff, yet some manual curation would have been needed in these specific cases and a few other obviously truncated sequences where the DM domain (the only portion shared by all sequence groups) was partial (or even entirely missing, in one case).

Powered by Editorial Manager® and ProduXion Manager® from Aries Systems Corporation

Answer: Thanks for the suggestion. We have done manual curation of the MSA after the automatic trimming and made sure we only kept regions that were conserved over sequences. We have also made sure that all domains (DM, Dmrt1, Dsx, DMA and DMB) were properly aligned. The final MSA is being submitted to GigaDB (DMRT_seqs_trimmed.fa) to be publicly available.

Reviewer 2

1. The authors should provide more detailed descriptions of the sequencing data and the assembly process.

Answer: We thank the reviewer for providing valuable input and suggestions in their review of the paper. Details on the assembly process may be found in section “Genome assembly”, and a file containing the commands ran for the assembly was submitted to the GigaDB public database (assembly.sh). As for sequencing data, the accession numbers of all genomic datasets are available under the section “Data availability”. Information on the RNA-seq data used to support gene prediction has been added in section “Gene prediction” and in the (new) Supplementary Table S2.

2. The authors should discuss the potential limitations in using the reference genome to control the invasion.

Answer: A discussion on those limitations has been added to the section “Re-use potential”. More specifically, in the following paragraphs:

“However, a reference genome based on the sequencing of a single specimen does not encompass the genetic diversity of the species. This limitation can compromise the development of efficient biotechnology-based control solutions that rely on specific target gene sequences. For example, Single Nucleotide Polymorphisms (SNPs) at the target site of a CRISPR-Cas9-based gene drive strategy can confer resistance to Cas9 cleavage, rendering the control strategy ineffective [53]. To identify potential critical SNPs and select invariant target sites, it will be necessary to resequence regions of interest in multiple individuals.
Structural variants have been detected in other mollusc species. In those species, a pattern of presence absence variation (PAV) has been reported, in which some genes are present only in some individuals of the population [44]. The chromosome-level genome may be used as a reference for future studies resequencing multiple golden mussel individuals to check whether the species is also under PAV and, if so, which parts of the genome are more or less conserved between individuals. Ultimately, only genes that are not subject to PAV should be considered potential targets for biotechnology-based control strategies.”

3. The authors should discuss the implications of the findings in a broader context.

Answer: We have also aimed at improving that by adding some content in the “Re-use potential” section: “The chromosome-level genome can also be used as a reference for future population genomic studies. Understanding genomic variation among different golden mussel populations may unveil the routes of dispersion in invaded areas and support better control policies. Besides that, the new genome will support the study of chromosome evolution within Lophotrochozoan and Mytilidae through the comparison to chromosome-level genomes of related species. Lastly, the higher contiguity and accuracy of the new genome greatly benefits gene prediction quality aiming to more reliable studies of the evolution of genes and gene families.”

4. The results of Merqury showed that the QV score of 53 of the chromosome-level genome was 99.999%, indicating a high level of accuracy. It is also recommended to conduct analysis of genome completeness using Merqury to further assess the quality of the genome assembly. In addition, it is also suggested to add genome survey related analysis, as this can help obtain the size and heterozygosity of the genome, which are essential for guiding genome assembly and determining the redundancy of

	the assembly results.<br><br>Answer: Thanks for the suggestion. We have added a new section (“Overall genome characteristics”) containing genome statistics (e.g., heterozygosity rate) based on k-mer analysis of the PacBio HiFi reads:<br><br>“A k-mer based approach was used to estimate overall genome statistics from the PacBio HiFi data. Jellyfish [26] was used to calculate the frequencies of 31-bp long k-mers and GenomeScope [27] was used to build a model to infer genome characteristics. The genome was inferred to be diploid, with an estimated haploid size around 1.3 Gb (Supplementary Figure S1). The expected repeat content was 43% and a high heterozygosity rate was estimated (2.4%).”<br><br>We have also included more data (section “Genome assembly”) from the Merqury analysis, such as the requested completeness (Table 1) and also the Merqury plots (Supplementary Figure S2). The new sentence in the manuscript read:<br><br>“We used Merqury [34] with the PacBio HiFi reads and calculated an assembly-contained-kmer completeness of 99.23%, and a QV of 53, representing a base accuracy of 99.999% (Merqury plots can be found in Supplementary Figure S2)”<br><br><br>5. Please explain why authors chose to compare assembly metrics with Illumina-based genomes, while reference 23 reported a "hybrid-hierarchical genome assembly".<br><br>Answer: This is a confusion caused by the way we decided to call the first assembly: although the first version of the golden mussel genome (first assembly) was done with a hybrid strategy (Illumina plus low coverage of PacBio), most of the sequencing data used was Illumina, so we ended up naming it “Illumina-base”. However, we understand that it may be misleading and we have decided to change the way we name the different assemblies. In the revised version of the manuscript, the first assembly is now called “draft”, while the one reported in this study is referred to as “chromosome-level”.
**Additional Information:**	
**Question**	**Response**
Are you submitting this manuscript to a special series or article collection?	No
**Experimental design and statistics**<br><br><br>Full details of the experimental design and statistical methods used should be given in the Methods section, as detailed in our Minimum Standards Reporting Checklist. Information essential to interpreting the data presented should be made available in the figure legends.<br><br><br>Have you included all the information requested in your manuscript?	Yes
**Resources**<br><br><br>A description of all resources used, including antibodies, cell lines, animals	Yes

Powered by Editorial Manager® and ProduXion Manager® from Aries Systems Corporation

and software tools, with enough information to allow them to be uniquely identified, should be included in the Methods section. Authors are strongly encouraged to cite Research Resource Identifiers (RRIDs) for antibodies, model organisms and tools, where possible.<br><br>Have you included the information requested as detailed in our Minimum Standards Reporting Checklist?	
**Availability of data and materials**<br><br>All datasets and code on which the conclusions of the paper rely must be either included in your submission or deposited in publicly available repositories (where available and ethically appropriate), referencing such data using a unique identifier in the references and in the "Availability of Data and Materials" section of your manuscript.<br><br>Have you have met the above requirement as detailed in our Minimum Standards Reporting Checklist?	Yes

Powered by Editorial Manager® and ProduXion Manager® from Aries Systems Corporation

Manuscript Click here to access/download;Manuscript;Manuscript-Aug 22.docx

A chromosome-level assembly supports genome-wide investigation of the DMRT gene family in the golden mussel (*Limnoperna fortunei*)

João Gabriel R. N. Ferreira[1,2], Juliana A. Americo[1&], Danielle L. A. S. do Amaral[1], Fábio Sendim[1,2], Yasmin R. da Cunha[1,2], Tree of Life Programme[3], Mark Blaxter[3] , Marcela Uliano-Silva[3]* & Mauro de F. Rebelo[2]*

[1] Bio Bureau Biotecnologia, Rio de Janeiro, Brazil,

[2] Instituto de Biofísica Carlos Chagas Filho, Universidade Federal do Rio de Janeiro, RJ, Brazil,

[3] Tree of Life, Wellcome Sanger Institute, Hinxton, United Kingdom

[&] Corresponding author

*Contributed equally to this work

João Gabriel Rodinho Nunes Ferreira [0000-0002-5226-7159];

Juliana Alves Americo [0000-0001-9443-6144];

Danielle L A S do Amaral [0000-0002-8490-7464];

Fábio Sendim [0000-0002-8205-6416];

Yasmin R da Cunha [0000-0003-4832-2167];

Darwin Tree of Life Consortium;

Marcela Uliano-Silva [0000-0001-6723-4715];

Mauro de Freitas Rebelo [0000-0002-7809-0486];

// Abstract

Background

The golden mussel (*Limnoperna fortunei*) is a highly invasive species that causes environmental and socioeconomic losses in invaded areas. Reference genomes have proven to be a valuable resource for studying the biology of invasive species. While the current golden mussel genome has been useful for identifying new genes, its high fragmentation hinders some applications.

Findings

In this study, we provide the first chromosome-level reference genome for the golden mussel. The genome was built using PacBio HiFi, 10X and Hi-C sequencing data. The final assembly contains 99.4% of its total length assembled to the 15 chromosomes of the species and a scaffold N50 of 97.05 Mb. A total of 34 862 protein-coding genes were predicted, of which 84.7% were functionally annotated. A significant (6.48%) proportion of the genome was found to be in a hemizygous state. Using the new genome, we have performed a genome-wide characterization of the DMRT gene family, which has been proposed as a target for population control strategies in other species.

Conclusions

From the applied research perspective, a higher quality genome will support genome editing with the aim of developing biotechnology-based solutions to control invasion. From the basic research perspective, the new genome is a high-quality reference for molecular evolutionary studies of Mytilida and other Lophotrochozoa, and it may be used as a reference for future resequencing studies to assess genomic variation among different golden mussel populations, unveiling potential routes of dispersion and helping to establish better control policies.

Keywords

Golden mussel; *Limnoperna fortunei*; genome; invasive species; sex differentiation; DMRT

Data description

Context

Limnoperna fortunei (NCBI:txid356393) — popularly known as the golden mussel — is a freshwater bivalve species native to Southeast China which has successfully established itself as an invasive species in other Asian countries (Cambodia, Japan, Laos, South Korea, Taiwan, and Thailand) and in several South American countries (Argentina, Brazil, Paraguay, and Uruguay) [1]. Because of its impact on ecosystem structure and function, the golden mussel is considered an efficient ecosystem engineer, and its establishment is associated with changes in local biodiversity and nutrient recycling [2,3]. Socioeconomic impacts are also relevant where golden mussel aggregates bind and obstruct net cages and hydroelectric power plant equipment [4,5]. In the Brazilian hydroelectric sector alone it is estimated that the golden mussel causes an annual 120 million dollar loss due to longer and more frequent stops for maintenance [6]. Current control strategies have proven to be ineffective and the species has continued to spread. Alternative biotechnological solutions have been proposed [6] and one possibility is to apply molecular tools to disrupt genes involved in reproductive behavior. This has been tested in other species, such as the malaria mosquito, where a disrupted genotype is rapidly spreading through the population using a gene drive system [7,8].

The Doublesex and Mab-3 related transcription factor (DMRT) gene family is highly conserved in animals and contains members that play important roles in sexual differentiation. DMRT genes regulate gene expression through a conserved zinc finger DNA binding domain named DM. Most animals contain multiple DMRT genes, which act in developmental processes such as somitogenesis, neurogenesis and gametogenesis [9–11]. The doublesex (Dsx) gene is present in insects and is required for both male and

female sexual differentiation according to the sex-specific isoform that is produced after alternative splicing [12–14]. In nematodes, *mab-3* (male abnormal 3) acts as a critical factor for male sex determination [15,16]. In vertebrates, DMRT1 is required for masculinization of somatic cells [17,18]. In mollusks, it is assumed that DMRT1-like genes are involved in male sex differentiation, given the male-biased expression pattern in the gonads shared by many different species [19–21]. DMRT is an attractive candidate to disrupt golden mussel reproduction.

Reference genomes are an important resource for the study of invasive species. They have been used to study invasion dynamics, identifying molecular mechanisms conferring adaptiveness as well as promising genes for biotechnology-based control strategies [22]. The current genome assembly for the golden mussel [23] is a highly fragmented representation of the 15 chromosomes (2n=30) of the species [24,25] assembled mostly from Illumina sequencing reads. Limitations of this draft genome constrain its applications in resequencing and comparative genomic studies and may lead to incomplete or erroneous gene models, as indicated by the high number of missing (10%) and fragmented (7%) BUSCO genes reported in the original paper [23].

Recent advances in library preparation protocols, sequencing technologies and bioinformatics algorithms have made the development of high quality reference genomes scalable and affordable. In this study, we present a high quality genome for the golden mussel where we have identified a widespread occurrence of hemizygosity over the chromosomes. We identified four DMRT genes in the golden mussel genome, which have been compared to DMRT genes from other bivalve species to study the evolution of this gene family in the class. One golden mussel DMRT is a putative sex differentiation gene showing male-biased expression in the gonads; therefore, it is a potential target for biotechnology-based control strategies. The new golden mussel genome is expected to be a valuable reference for future studies on the species.

Sample collection

Golden mussel specimens were collected from the Taquari River, São Paulo, Brazil (23°16'45.7"S 49°12'01.7"W) on March 17, 2021. Three representative specimens were deposited in the molluscan collection of the National Museum administered by the Federal University of Rio de Janeiro (identification numbers: IB UFRJ 19950, IB UFRJ 19952 and IB UFRJ 19954). Other specimens were taxonomically identified by Dr. Igor Christo Miyahira. Finally, a set of specimens had their tissues – gonads, adductor muscle, digestive gland, gills, and foot – dissected and preserved in dry ice at -80ºC until and during transportation to the Wellcome Sanger Institute (WSI) in Hinxton, Cambridgeshire, United Kingdom for further processing and sequencing.

DNA extraction

DNA extraction was performed at the WSI's Tree of Life laboratory. Golden mussel samples were weighed and disrupted using a Covaris cryoPREP Automated Dry Pulveriser that subjects tissue – gill tissue was selected – to multiple impacts until it becomes a fine powder. Twenty five mg of this powder were used for DNA extraction and 50 mg were set aside for Hi-C. DNA extraction was performed using a Qiagen MagAttract HMW DNA extraction kit on a KingFisher APEX liquid handling system. Fifty nanograms of DNA were submitted to 10X genomic sequencing with any low molecular weight DNA removed prior to sequencing using a 0.8X AMpure XP purification kit. Similarly, prior to submission to PacBio sequencing, high molecular weight DNA was sheared to an average fragment size of between 12 and 20 kb using a MegaRuptor 3 (speed setting 30). The sheared DNA was purified by solid-phase reversible immobilization using AMpure PB beads with a 1.8X ratio of beads to sample. The concentration of sheared DNA was assessed using a Qubit Fluorometer with Qubit dsDNA High Sensitivity Assay kit and Nanodrop spectrophotometer, while the fragment size distribution was assessed using an Agilent FemtoPulse.

Sequencing

All sequencing libraries were constructed using DNA extracted from a single specimen, a female golden mussel with the unique Tee of Life identifier xbLimFort5. PacBio HiFi circular consensus and Chromium 10X Genomics linked-reads sequencing libraries were constructed according to the manufacturers' instructions. Sequencing was performed by the Scientific Operations core at WSI on PacBio SEQUEL II (HiFi) and Illumina NovaSeq (10X) instruments. Hi-C data were generated using the Arima v2.0 kit and sequenced on a NovaSeq 6000 instrument (RRID:SCR_016387).

Overall genome characteristics

A k-mer based approach was used to estimate overall genome statistics from the PacBio HiFi data. Jellyfish [26] was used to calculate the frequencies of 31-bp long k-mers and GenomeScope (RRID:SCR_017014) [27] was used to build a model to infer genome characteristics. The genome was inferred to be diploid, with an estimated haploid size around 1.3 Gb (Supplementary Figure S1). The expected repeat content was 43% and a high heterozygosity rate was estimated (2.4%).

Genome assembly

The genome assembly pipeline is summarized in Figure 1. The initial set of contigs was assembled using HiFiasm (RRID:SCR_021069) v0.16.1 combining HiFi and Hi-C reads in the Hi-C integrated mode [28]. 10X linked-reads were mapped to contigs using LongRanger v2.2.2 [29] and then Freebayes (RRID:SCR_010761) v1.3.1 [30] was used to polish the contigs based on the 10X mapping. The polished contigs were then scaffolded using the YaHS pipeline v1.0 [31]. Finally, scaffolds were manually curated by WSI's Genome Reference Informatics Team (GRIT) following the protocol described by Howe and colleagues (2021) [32]. The curated scaffolds represent the final genome

assembly, which was then annotated using the Ensembl Rapid Annotation Pipeline [33]. The mitochondrial genome was assembled using the MitoHiFi pipeline [34].

Figure 1. Genome assembly pipeline.

The size of the final genome assembly is 1.34 Gb (Supplementary Table S1). The vast majority (99.24%) of its total length is distributed over the 15 largest scaffolds (Figure 2), which correspond to the haploid chromosome number (n=15) of the species [24]. The largest contig and the largest scaffold are 8.3 Mb and 115 Mb long, respectively, and the genome GC content is 33.6%. BUSCO (RRID:SCR_015008) v5.0 [33] completeness was 95.6% (with the metazoa_odb10 dataset). We used Merqury (RRID:SCR_022964) [34] with the PacBio HiFi reads and calculated an assembly-contained-kmer completeness of 99.23%, and a QV of 53, representing a base accuracy of 99.999% (Merqury plots can be found in Supplementary Figure S2). All the quality metrics calculated for the new assembly conform to the standards of the Vertebrate Genomes Project (VGP) for what is considered a high-quality genome [35].

Figure 2. The genome landscape. A) Circos representation of the 15 chromosomes assembled in this study. Each track represents: i) the size of each chromosome, ii) the gene density, and the iii) repeat density over the chromosome sequences, calculated using a 2 Mb window size. B) Hi-C contact map with chromosomes displayed in size order from top to bottom and from left to right.

Table 1 presents genomic statistics of the previous draft assembly and the chromosome-level reference produced in this study. The chromosome-level reference scaffold N50 is 313-fold greater than its predecessor draft genome. An improvement has also been achieved in genome completeness as shown by an increase in both k-mer based completeness assessment and the percentage of complete BUSCO genes found.

Table 1. Comparison of assembly metrics between the draft and the new golden mussel genome.

	**Draft genome (GCA_003130415.1)**	**Chromosome-level genome (GCA_944474755.1)**
**Total assembly length (Gb)**	1.67	1.34
**GC content (%)**	33.6	33.8
**Number of scaffolds**	20 580	309
**Scaffold N50 (Mb)**	0.31	97.05
**Scaffold L50**	1 489	7
**Number of contigs**	61 175	1 838
**Contig N50 (Mb)**	0.03	1.50
**Contig L50**	16 521	277
**QV**	14.89	53.36
**Completeness (%)**	47.83	68.55 (primary assembly)<br>99.23 (primary + alternate haplotype*)
**BUSCO (metazoa_odb10)**	C:66.8% [S:65.0%,D:1.8%], F:19.3%,M:13.9%,n:954	C:95.6% [S:95.0%,D:0.6%], F:2.2%,M:2.2%,n:954

BUSCO statistics. C=complete; S=complete and single-copy; D=complete and duplicated; F=fragmented; M=missing; n=number of BUSCO genes from reference dataset.
* primary assembly: GCA_944474755.1; alternate haplotype: GCA_944589985.1

Repeat annotation

Detection and classification of repeat elements was done using the Earl Grey pipeline v1.3 [36]. Earl Grey was run with the RepeatMasker (RRID:SCR_012954) search term (-r) set to “mollusca”. Almost half (46.93%) of the genome was annotated as repetitive sequences, with 35.80% of the genome labeled as unclassified repeats (Table 2). Similarly high proportions of unclassified repeats have been reported in other mussels [37,38]. The second most frequent repeat class detected was Long interspersed nuclear elements (LINE), representing 4.51% of the total genome.

Table 2. Repetitive elements identified in the golden mussel genome.

Classification*	Total sequence length (bp)	Sequences count	Proportion of genome (%)	Number of distinct classifications
DNA	46 269 487	64 938	3.46	201
LINE	60 245 208	64 949	4.51	224
LTR	28 342 781	50 395	2.12	112
Other (Simple Repeat, Microsatellite, RNA)	216 907	244	0.02	2
Penelope	11 115 342	24 065	0.83	23
Rolling Circle	1 640 436	1 503	0.12	6
SINE	831 095	723	0.06	3
Unclassified	478 115 783	883 466	35.80	1 494

LINE=Long interspersed nuclear elements; LTR=Long terminal repeats; SINE=Short interspersed nuclear element
* Classification in alphabetical order.

Gene prediction

The Ensembl rapid annotation pipeline [33] was used to predict genes. The prediction was supported by homologous proteins and pre-existing golden mussel RNA-seq data, including RNA-seq data from the draft genome project and from the same specimen (xbLimFort5) sequenced for the chromosome-level genome assembly (Supplementary Table S2). A total of 34 862 protein-coding genes were predicted, with 68 899 proteins inferred. Most (53.5%) genes were associated with a single protein, with about 21.8% associated with two proteins, and 24.7% with three or more proteins (Supplementary Table S3). In addition to the protein-coding genes, 58 911 non-coding genes were predicted, most of which (56.5%) were classified as long non-coding RNA (lncRNA) (Table 3).

The number of predicted genes was significantly lower compared to the one reported for the draft genome (60 717) [23]. The draft nature of the previous genome could be a factor influencing prediction, because the genome was assembled in more than 20 000 scaffolds and the QV was low, and predictions can become fragmented and or truncated, overinflating the number of genes. This is in line with our new results, as even though the number of predicted genes for the chromosome-level assembly is lower (34 862), this prediction is more complete as evidenced by a (i) 2.3-fold increase in the number of BUSCO genes found (Supplementary Table S4), (ii) decrease of duplicated and missing BUSCOs and a (iii) 1.8-fold increase in the number of RNAseq reads mapped (Supplementary Data Note 1 - Table 1).

Table 3. Categories of predicted genes.

Statistics	Value
Protein-coding genes	34 862
Non-coding genes	58 911
lncRNA	33 258
Y_RNA	9 316
tRNA	7 582
ribozyme	5 091
misc_RNA	1 641
rRNA	1 410
snRNA	565
snoRNA	47
scaRNA	1

lncRNA=long non-coding RNA; tRNA=transfer RNA; misc_RNA=miscellaneous RNA; rRNA=ribosomal RNA; snRNA=small nuclear RNA; snoRNA=small nucleolar RNA; scaRNA=small Cajal body-specific RNA.

Table 4. Gene prediction statistics.

Statistics	Value
Average gene length (bp)	9 426
Protein-coding genes (bp)	17 765
Non-coding genes (bp)	4 492
Exons	719 821
Average exon length (bp)	229
Proteins	68 899
Average protein length (aa)	462
Gene density (No. genes/100kb)	7.02
Protein-coding genes	2.61
Non-coding genes	4.41

Functional annotation

The longest protein inferred from each gene was selected using the primary_transcript.py script from OrthoFinder (RRID:SCR_017118) v2.5.4 [39]. These proteins were aligned against the SwissProt database (downloaded on June 2, 2022) using BLASTP v2.12.0+ from blast+ package [40] and against the NR database (downloaded on June 24, 2022) using Diamond v2.0.15.153 [41]. Both alignments were done using a threshold of $1e^{-5}$ for the e-value parameter. Out of the 34 862 protein-coding genes, 19,899 (57.08%) had at least one hit against the curated SwissProt database (Figure 3). The eggNOG mapper v2 web server [42] was used to attribute GO terms and KEGG pathways to each gene. At least one GO term and at least one KEGG pathway was associated with 9 746 (27.96%) and 6 183 (17.74%) genes, respectively. To annotate protein domains, an alignment against Pfam (RRID:SCR_004726) was done using the hmmsearch (e-value threshold of $1e^{-5}$) command from HMMER v3.3.1 [43]. A total of 20 963 (60.13%) genes was associated with at least one protein domain. Sequences were labeled as “unannotated” when they did not have a hit to any of the five databases searched (NR, SwissProt, GO, KEGG and Pfam).

Figure 3. UpSetPlot representing the different functional annotations. Horizontal bars on the left represent the total number of genes annotated according to each database. Vertical bars represent overlapping annotations (i.e. genes annotated by a single or a combination of databases), as indicated by the connected dark green circles.

Comparative genomics with other mollusks

Seven bivalves and one gastropod (*Pomacea canaliculata*) species were chosen to search for orthologs to the golden mussel genes (Supplementary Table S5). All proteomes were processed with OrthoFinder's primary_transcript.py script to retrieve only the longest protein associated with each gene. The processed proteomes were then used as input to run OrthoFinder v2.5.4 [39] with default parameters.

Overall, OrthoFinder was able to assign 436 439 genes to orthogroups, representing 86.9% of all mollusks' genes (Supplementary Table S6). The species tree, built with STAG based on the orthogroups, placed species in the expected families, with the gastropod *P. canaliculata* used as the outgroup to root the tree (Figure 4A). Most species had a high proportion of genes assigned to orthogroups, with D. polymorpha showing an inflated number of genes (Figure 4A and Supplementary Table S7). Of all 50 219 orthogroups identified, 7 616 (15.2%) had genes from all nine mollusk species (Supplementary Table S6). For the golden mussel, 30 508 genes (87.5%) were assigned to an orthogroup, with 823 orthogroups assigned as golden mussel specific (Supplementary Table S7). As expected, the species that shared the largest number of genes (14 411) with the golden mussel was *Mytilus galloprovincialis*, which belong to the same family (Mytilidae) (Figure 4B).

Figure 4. OrthoFinder results for the nine mollusk species studied. A) (left) Species tree constructed based on the inferred orthogroups and (right) gene counts for different categories. B) Number of genes shared between each pair of species. The darker the red the smaller the number of shared genes, while the darker the blue the greater their number.

Hemizygosity investigation

We searched for hemizygous regions in the golden mussel genome applying the pipeline of Calcino *et al.* [44] for structural variant detection with a few modifications to use HiFi reads in the analyses (protocol in Supplementary Data Note 2). The pipeline maps reads back to the reference and identifies structural variations using *pbsv* [45] and further scripts. Hemizygous regions can be insertions (subset of reads that have sequence that is not present in the reference), or deletions (where the reference has sequence not present in a subset of the mapped reads). Considering only the detected deletions, the percentage of the golden mussel genome flagged as hemizygous was 6.48%, which is in the range observed for other molluscan species (0.17 - 6.69%) [56]. If we also consider insertions, the hemizygous content increases to 9.79%, which is also in the range of other molluscan species (0.37 - 10.81%) [56]. The chromoMap package was used to plot the distribution of the hemizygous regions over the chromosome-level scaffolds (more details in Supplementary Data Note 2). As observed in other molluscan species, the hemizygous regions were widespread and were not restricted to specific chromosomes or chromosomal regions (Figure 5) [56].

Figure 5. Distribution of hemizygous regions over the 15 chromosome-level scaffolds. The vertical red lines represent the location of the hemizygous regions.

A k-mer count analysis of the sequences in hemizygous regions was performed. A k-mer coverage plot was built for (i) the reads mapped to the whole genome (i.e. to any genomic region); and (ii) the subset of reads that mapped only to the hemizygous regions (Supplementary Data Note 2). The mapped reads used for k-mer coverage analysis were also employed to calculate the read coverage (over sliding windows of 1 kb) of hemizygous regions and to compare it with the read coverage over the whole genome (Supplementary Data Note 2). For both analyses (kmers and read coverage), we see

hemizygous reads falling in the coverage of the heterozygous (1n) regions when compared with the whole genome analysis (Figure 6), affirming that they occur only in one haplotype of the assembly.

Figure 6. Analysis of kmer and read coverage of hemizygous regions. A) The K-mer plots represent the k-mer counts for a k=21. The upper plot was built from all reads mapped to the genome, while the lower plot was built using only reads mapped to hemizygous (more specifically, deletions) regions. B) The read coverage plots were built from a median read coverage calculation of 1Kp windows. The upper plot represents the coverage over the whole genome and the lower plot over the hemizygous regions. For all plots the black vertical lines represent the 1n coverage peak.

DMRT gene family analysis

In addition to the seven bivalve species used in the orthology inference analysis, seven non-bivalve model organisms were chosen to search for potential DMRT genes (Supplementary Table S8). Those species were included because they already have well characterized DMRT genes that could be used to guide the interpretation of the phylogeny. All non-bivalve and bivalve proteomes were processed with the primary_transcripts.py script to get a single (the longest) protein per gene. The processed proteomes were aligned against the Pfam-A database to annotate protein domains. The alignment was done using the hmmscan command from the HMMER (RRID:SCR_005305) v3.1b2 program [43] with a threshold value of $1e^{-5}$ for the -E parameter. After protein domain annotation, all proteins that had one of the following domains were selected as potential DMRT genes: DM (PF00751), DMA (PF03474), DMRT-like (PF15791) or Dmrt1 (PF12374). Additionally, MAB-3 sequence from *Caenorhabditis elegans* (Uniprot Accession O18214) was included due to its well established role in sex differentiation. The potential DMRT proteins were aligned using the clustalw command from CLUSTAL v2.1 [45] and the alignment was trimmed using the trimAl tool (RRID:SCR_017334) v1.4 [46] with the "-automated1" option. After an initial phylogeny tree inference, sequences belonging to clades with no bivalve sequences were removed. Manual inspection to check for remaining isoforms and split gene models was also carried out (Supplementary Data Note 3). The remaining proteins were aligned and trimmed using CLUSTAL and trimAI, followed by manual inspection of the trimmed alignment. The VG+I+G4 model was chosen according to ModelTest-NG v0.1.7 [47] and used to build the final tree with MrBayes (RRID:SCR_012067) v3.2.7a [48,49] for 10 000 000 MCMC generations. Convergence was evaluated by checking if the standard deviation of split frequencies was <0.01. The consensus tree was then manipulated using iToL (RRID:SCR_018174) [50,51] to generate the final figure.

The final DMRT tree was midrooted since no a priori outgroup could be set. Bivalve orthologs to DMRT1L, DMRT2, DMRT3 and DMRT4/5 genes were found (Figure 7). The golden mussel genome contains a single copy for each of the four DMRT genes, as well as *Mytilus galloprovincialis, Mizuhopecten yessoensis* and *Pecten maximus*. A single DMRT2 gene was found in all bivalve species, except for *Crassostrea gigas*, *Crassostrea virginica* and *Dreissena polymorpha*, for which no DMRT2 gene was found (Figure 7; Supplementary Table S9). While DMRT2 genes in vertebrates and insects have shown a single DM domain, most bivalve DMRT2 genes have also shown a C-terminal DMA domain.

After manual correction of a false duplication in *D. polymorpha* (details in Supplementary Data Note 3), DMRT3 genes were found in single copy in all bivalve species. Just like vertebrate and insect genes, DMRT3 from bivalves have both a DM and a DMA domain. DMRT4/5 genes were also found in single copy in all species, except *D. polymorpha* and *Mercenaria mercenaria*, in which three potential DMRT4/5 genes were identified. Most bivalve DMRT4/5 genes have a DM and a DMA domain, except a gene from *D. polymorpha* (KAH3699546.1) and a gene from *M. mercenaria* (XP_045157053.1) that are evolutionarily more distant to the other bivalve DMRT4/5 genes and could therefore represent a different DMRT gene type.

Figure 7. Phylogenetic tree of DMRT genes. Golden mussel genes are marked in bold. The domain representation of the *M. galloprovincialis* gene (VDI32052.1) was shortened (represented by a double slash) due to its significantly larger length for better visualization.

After manual removal of a false duplication in *C. virginica* (details in Supplementary Data Note 3), the DMRT1L genes were found in single-copy in all bivalve species except in *M. mercenaria* and *D. polymorpha* where DMRT1L seems to be missing. Bivalve DMRT1L genes missed both Dmrt1 domain (vertebrate-related) and Dsx domain (insect-related), containing only DM domains. Some bivalve DMRT1L genes contain a single DM domain,

while others (e.g. the golden mussel) contain two DM domains, like MAB-3 from *Caenorhabditis elegans*. DMRT1L bivalve sequences were split into three monophyletic clades: (i) a clade containing Mytilidae (*L. fortunei* and *M. galloprovincialis*) sequences; (ii) another clade containing genes from *C. virginica* and *C. gigas*; and (iii) a clade containing sequences from *P. maximus* and *M. yessoensis*. All DMRT1L clades contained genes whose expression was shown to be male-biased. *C. gigas* DMRT1L has shown to have significantly higher expression in male gonads [19], the same pattern observed for *M. yessoensis* [20]. Regarding the golden mussel, a DMRT-like transcript (GGt_299830_c0_g1_i1) has shown to have male-biased expression in the gonads [52]. We have aligned that transcript against the chromosome-level genome of the golden mussel and verified that it matches the ENSLFOG00000002085.1 gene, which is part of the putative DMRT1L clade. Despite the relevant changes in the sequences of the DMRT1L genes in different bivalve species, it seems that they have kept the characteristic feature of having male-biased expression, which we assume has to do with their role in male sex differentiation.

Re-use potential

In this study, we present a chromosome-level genome for the golden mussel. The high quality and contiguity of this genome will benefit downstream studies that focus on either studying individual gene families of interest or genomic evolution at the chromosome level. One project that will immediately benefit from the new genome is the development of a biotechnology-based solution to control invasive golden mussel populations, which are under development [6]. In the current study, we have identified a putative sex determination/differentiation gene (DMRT1L) in the golden mussel that stands out as a potential target for the control strategy. Further studies should be conducted to confirm that DMRT1L disruption induces incapacity of male golden mussels to sexually develop.

However, a reference genome based on the sequencing of a single specimen does not encompass the genetic diversity of the species. This limitation can compromise the development of efficient biotechnology-based control solutions that rely on specific target gene sequences. For example, Single Nucleotide Polymorphisms (SNPs) at the target site of a CRISPR-Cas9-based gene drive strategy can confer resistance to Cas9 cleavage, rendering the control strategy ineffective [53]. To identify potential critical SNPs and select invariant target sites, it will be necessary to resequence regions of interest in multiple individuals.

Structural variants have been detected in other mollusc species. In those species, a pattern of presence absence variation (PAV) has been reported, in which some genes are present only in some individuals of the population [44]. The chromosome-level genome may be used as a reference for future studies resequencing multiple golden mussel individuals to check whether the species is also under PAV and, if so, which parts of the genome are more or less conserved between individuals. Ultimately, only genes that are not subject to PAV should be considered potential targets for biotechnology-based control strategies.

The chromosome-level genome can also be used as a reference for future population genomic studies. Understanding genomic variation among different golden mussel populations may unveil the routes of dispersion in invaded areas and support better control policies. Besides that, the new genome will support the study of chromosome evolution within Lophotrochozoan and Mytilidae through the comparison to chromosome-level genomes of related species. Lastly, the higher contiguity and accuracy of the new genome greatly benefits gene prediction quality aiming to more reliable studies of the evolution of genes and gene families.

Discussion

The new reference genome for *Limnoperna fortunei* reported in this study has better contiguity, completeness and accuracy metrics compared to the draft assembly, meaning that it is a more complete and reliable resource of information for the study of the golden mussel. Previous studies have shown that highly fragmented draft genomes can contain errors even in coding regions, jeopardizing experimental and *in silico* studies that use its sequences as a reference. For instance, Korlach et al. have shown that the draft genome of two avian species had a series of misassemblies that generated issues (e.g. missing sequences and base call errors) in coding sequences and/or its flanking regions, and those issues could be resolved after a new assembly based on PacBio long reads [54]. The high quality reference genome reported in this study increases the accuracy and completeness of genes of interest for the study of the golden mussel, supporting both fundamental and applied research on this invasive species. In addition to that, the high contiguity of the assembly opens the door to comparative studies at a chromosome scale, shedding light on the evolution of the golden mussel and other genomes.

Structural variation analysis showed that a significant (6.48%) proportion of the golden mussel genome was in a hemizygous state, i.e. the region is present in only one of the homologous chromosomes. The presence of hemizygous regions has already been seen in other molluscan species [44,55]. Compared to the eight molluscan species analyzed in Calcino et al [44], the golden mussel showed the second largest proportion of hemizygosity, only lower than the bivalve *Scapharca* (*Anadara*) *broughtonii* (6.69%). In another Mytilidae species (*Mytilus galloprovincialis*), it has been shown that the presence of hemizygous regions is correlated with the occurrence of gene presence/absence variation (PAV) [55], which, as discussed in the “Re-use potential” section, may have impacts on biotechnology-based control strategies. Future re-

sequencing studies should allow us to move from a genome to a pangenome scenario and to check what set of golden mussel genes (if any) is under PAV.

The DMRT gene family is known for its role in sex determination and differentiation and it has been proposed as a target for biotechnological population control strategies in the malaria mosquito [8]. Using the chromosome-level genome assembled in this study, we have done the first genome-wide characterization of the DMRT gene family in the golden mussel and we were able to identify DMRT1L, DMRT2, DMRT3 and DMRT4/5 orthologs. DMRT2/DMRT11E genes show varying functions. In mouse, DMRT2 is involved in axial skeleton development, while in zebrafish DMRT2a/2b play roles in left-right patterning [56]. However, in arthropods DMRT11E has shown to play a role in sex differentiation. Knockdown of *Drosophila melanogaster* DMRT11E causes sperm malformation [57], while DMRT11E is required for proper oogenesis in the silkworm *Bombyx mori* [58]. DMRT2 function in mollusks is still unclear, however studies of expression profiles suggest its participation in spermatogenic cell differentiation in the pearl oyster *Pinctada fucata* and in the scallop *Chlamys nobilis* [59,60].

In mammals, DMRT3 plays a role in neurogenesis, with mutations associated with locomotion problems in horses and spinal circuit malfunction in mice [61]. DMRT3 has high expression in testis in some mammalian and fish species, suggesting a potential role in testicular development [62,63]. DMRT4/5/99B genes have a well-conserved function in different species being mainly involved in neurogenesis. Mutations of DMRT4 and DMRT5 in vertebrates cause neuronal abnormalities [64–66], just like mutations do in the DMRT99B in arthropods [67,68]. As far as we know, no mutation study has been carried out on mollusks to explore DMRT5 function, although its tissue-wide distribution and expression indicates it may play a role in early embryonic development and various biological processes in *C. nobilis* [59].

Dsx (arthropods), MAB-3 (nematodes) and DMRT1 (vertebrates) genes are members of the DMRT family historically associated with sex determination and differentiation roles [15,69,70]. Although sharing the same function, there is some debate as to whether those genes share a common ancestor. Based on phylogenetic and synteny analyses, Mawaribuchi et al. concluded that those three genes form separate clusters and therefore might have emerged independently in each clade [71]. The phylogenetic analysis for the DMRT family in our study is in agreement, with the addition of a cluster of sex differentiation genes specific to mollusks named DMRT1L. Those genes consistently share a pattern of male-biased expression in the gonads in many other mollusk species [19–21,72] and a recent study has confirmed that knockdown of the DMRT1L in *Crassostrea gigas* cause male gonads to fail to differentiate [73]. If DMRT1L knockdown in the golden mussel shows the same consequences, it can be a strong target for population control strategies of this invasive species.

Data availability

The genome sequence is available in the NCBI under accession GCA_944474755.1, while contigs representing the alternative haplotype are available as GCA_944589985.1. Raw data accessions are summarized in Table 5. All supporting data are available in the GigaScience GigaDB database [74].

Table 5. Accession numbers of raw sequencing data used for the genome assembly project.

Library	Accession(s)
Pacific Biosciences SEQUEL II (HiFi)	ERR9713989-91, ERR9713993
10X Genomics Illumina	ERR9503462-65
Hi-C Illumina	ERR9503466

List of abbreviations

DMRT - Doublesex and Mab-3 related transcription factor

GO - gene ontology

GRIT - Genome Reference Informatics Team

LINE - Long interspersed nuclear elements

lncRNA - long non-coding RNA

LTR - Long terminal repeats

misc_RNA - miscellaneous RNA

PAV - presence-absence variation

RPTP - Receptor-type protein tyrosine phosphatase

rRNA - ribosomal RNA

scaRNA - small Cajal body-specific RNA

SINE - Short Interspersed nuclear element

snRNA - small nuclear RNA

snoRNA - small nucleolar RNA

tRNA - transfer RNA

WSI - Wellcome Sanger Institute

VGP - Vertebrates Genome Project

Competing interests

The authors declare that they have no competing interests.

Funding

This work was financed by the Brazilian National Electric Energy Agency ANEEL R&D program (grant PD-10381-0419/2019) and by the Wellcome Sanger Core Award (220540/Z/20/A). We also thank CTG Brasil, Tijoá Energia and Spic Brasil for funding this project through the ANEEL R&D Program. João Gabriel R. N. Ferreira and Fábio Sendim were recipients of Ph.D. fellowships and Yasmin R. da Cunha was a recipient of a Master's fellowship from CAPES, a federal government agency of the Brazilian Ministry of Education, which supports graduate students and faculty. Genome sequencing and assembly was provided by the Wellcome Sanger Institute Tree of Life Programme in collaboration with the Bio Bureau Biotechnology company.

Author contributions

J.A.A., M.F.R., M.U-S. and M.B. designed the project. M.U-S. and J.G.R.N.F. planned the bioinformatics analyses. J.G.R.N.F. performed the bioinformatics and data analyses. J.G.R.N.F. wrote the first version of the manuscript. D.L.A.S.A, F.S. and Y.R.C. worked on the collection of golden mussel specimens and tissue dissection. All authors contributed to writing and approved the final manuscript.

Acknowledgments

We would like to extend our gratitude to Shane McCarthy, Chenxi Zhou and Andrew Calcino for insightful conversations and discussions, and to the Tree of Life laboratories and the Long Read Team in Sanger Scientific Operations team for their work in extraction and sequencing.

Tree of Life Programme author list

Table 6. A list of the Wellcome Sanger Tree of Life contributors to this Data Note. The author list can also be found in Zenodo [74].

**Programme Lead:** Mark Blaxter
**Associate Director: Delivery and Operations:** Ed Symons
**Head of Production Genomics:** Kerstin Howe
**Tree of Life Samples**<br>Nancy Holroyd, Edel Sheerin, Sophie Potter, Catherine McCarthy
**Tree of Life Laboratory**<br>Lead: Caroline Howard<br>Adam Bates, Isabelle Clayton-Lucey, Amy Denton, Andrew Griffiths, Benjamin Jackson, Haddijatou Mbye, Graeme Oatley, Juan Pablo Narváez Gomez, Liam Prestwood, David Rowland, Abitha Thomas, Aarushi Vaidya
**Tree of Life Assembly**<br>Lead: Shane A. McCarthy<br>Eerik Aunin, William Eagles, Noah Gettle, Ksenia Krasheninnikova, Eugene Myers, Damon-Lee Pointon, Ying Sims, James Torrance, Marcela Uliano-Silva, Chenxi Zhou
**Genome Reference Informatics Team**<br>Lead: Jonathan Wood<br>Dominic Absolon, Joanna Collins, Michael Paulini, Sarah Pelan, Alan Tracey, Bethan Manley

Ethics/compliance statement

The materials that have contributed to this Data Note are in compliance with the Brazilian Biodiversity Law.

References

1. : CBEIH. Centro de Bioengenharia de Espécies Invasoras de Hidrelétricas. https://base.cbeih.org/index.php Accessed 2022 Dec 14.

2. Boltovskoy D, Karatayev A, Burlakova L, Cataldo D, Karatayev V, Sylvester F, et al.. Significant ecosystem-wide effects of the swiftly spreading invasive freshwater bivalve Limnoperna fortunei. *Hydrobiologia*. 2009; doi: 10.1007/s10750-009-9956-9.

3. Cataldo D, O´ Farrell I, Paolucci E, Sylvester F, Boltovskoy D. Impact of the invasive golden mussel (Limnoperna fortunei) on phytoplankton and nutrient cycling. *Aquat Invasions*. Regional Euro-Asian Biological Invasions Centre Oy (REABIC); 2012; doi: 10.3391/ai.2012.7.1.010.

4. De Nys R, Guenther J. 8 - The impact and control of biofouling in marine finfish aquaculture. In: Hellio C, Yebra D, editors. *Advances in Marine Antifouling Coatings and Technologies*. Woodhead Publishing;

5. Prescott TH, Claudi R, Prescott KL. Impact of dreissenid mussels on the infrastructure of dams and hydroelectric power plants. *Quagga and zebra mussels: biology, impacts, and control*. CRC Press Boca Raton, FL; :315–292013;

6. Rebelo MF, Afonso LF, Americo JA, da Silva L, Neto JLB, Dondero F, et al.. A sustainable synthetic biology approach for the control of the invasive golden mussel (Limnoperna fortunei). PeerJ Preprints; 2018 Sep. Report No.: e27164v3.

7. Hammond A, Galizi R, Kyrou K, Simoni A, Siniscalchi C, Katsanos D, et al.. A CRISPR-Cas9 gene drive system targeting female reproduction in the malaria mosquito vector Anopheles gambiae. *Nat Biotechnol*. 2016; doi: 10.1038/nbt.3439.

8. Kyrou K, Hammond AM, Galizi R, Kranjc N, Burt A, Beaghton AK, et al.. A CRISPR–Cas9 gene drive targeting doublesex causes complete population suppression in caged Anopheles gambiae mosquitoes. *Nat Biotechnol*. Nature Publishing Group; 2018; doi: 10.1038/nbt.4245.

9. Kim S, Namekawa SH, Niswander LM, Ward JO, Lee JT, Bardwell VJ, et al.. A mammal-specific Doublesex homolog associates with male sex chromatin and is required for male meiosis. *PLoS Genet*. journals.plos.org; 2007; doi: 10.1371/journal.pgen.0030062.

10. Saúde L, Lourenço R, Gonçalves A, Palmeirim I. terra is a left–right asymmetry gene required for left–right synchronization of the segmentation clock. *Nat Cell Biol*. Nature Publishing Group; 2005; doi: 10.1038/ncb1294.

11. Yoshizawa A, Nakahara Y, Izawa T, Ishitani T, Tsutsumi M, Kuroiwa A, et al.. Zebrafish Dmrta2 regulates neurogenesis in the telencephalon. *Genes Cells*. Wiley Online Library; 2011; doi: 10.1111/j.1365-2443.2011.01555.x.

12. Burtis KC, Baker BS. Drosophila doublesex gene controls somatic sexual differentiation by producing alternatively spliced mRNAs encoding related sex-specific polypeptides. *Cell*. 1989; doi: 10.1016/0092-8674(89)90633-8.

13. Scali C, Catteruccia F, Li Q, Crisanti A. Identification of sex-specific transcripts of the Anopheles gambiae doublesex gene. *J Exp Biol*. 2005; doi: 10.1242/jeb.01819.

14. Shukla JN, Palli SR. Doublesex target genes in the red flour beetle, Tribolium

castaneum. *Sci Rep*. 2012; doi: 10.1038/srep00948.

15. Shen MM, Hodgkin J. mab-3, a gene required for sex-specific yolk protein expression and a male-specific lineage in C. elegans. *Cell*. Elsevier; 1988; doi: 10.1016/0092-8674(88)90117-1.

16. Zhou L, Ma X, Zhu N, Zou Q, Guo K, Bai L, et al.. The role of mab-3 in spermatogenesis and ontogenesis of pinewood nematode, Bursaphelenchus xylophilus. *Pest Manag Sci*. Wiley; 2021; doi: 10.1002/ps.6001.

17. Raymond CS, Murphy MW, O'Sullivan MG, Bardwell VJ, Zarkower D. Dmrt1, a gene related to worm and fly sexual regulators, is required for mammalian testis differentiation. *Genes Dev*. 2000; doi: 10.1101/gad.834100.

18. Yoshimoto S, Ito M. A ZZ/ZW-type sex determination in Xenopus laevis. *FEBS J*. 2011; doi: 10.1111/j.1742-4658.2011.08031.x.

19. Zhang N, Xu F, Guo X. Genomic analysis of the Pacific oyster (Crassostrea gigas) reveals possible conservation of vertebrate sex determination in a mollusc. *G3* . 2014; doi: 10.1534/g3.114.013904.

20. Li R, Zhang L, Li W, Zhang Y, Li Y, Zhang M, et al.. FOXL2 and DMRT1L Are Yin and Yang Genes for Determining Timing of Sex Differentiation in the Bivalve Mollusk Patinopecten yessoensis. *Front Physiol*. 2018; doi: 10.3389/fphys.2018.01166.

21. Evensen KG, Robinson WE, Krick K, Murray HM, Poynton HC. Comparative phylotranscriptomics reveals putative sex differentiating genes across eight diverse bivalve species. *Comp Biochem Physiol Part D Genomics Proteomics*. Elsevier; 2022; doi: 10.1016/j.cbd.2021.100952.

22. McCartney MA, Mallez S, Gohl DM. Genome projects in invasion biology. *Conserv Genet*. 2019; doi: 10.1007/s10592-019-01224-x.

23. Uliano-Silva M, Dondero F, Dan Otto T, Costa I, Lima NCB, Americo JA, et al.. A hybrid-hierarchical genome assembly strategy to sequence the invasive golden mussel, Limnoperna fortunei. *Gigascience*. academic.oup.com; 2018; doi: 10.1093/gigascience/gix128.

24. Ieyama H. Chromosomes and nuclear DNA contents of Limnoperna in Japan (Bivalvia: Mytilidae). *Venus*. jstage.jst.go.jp; 1996;

25. Reis AC, Amaral D, Americo JA, Rebelo MF, de Sousa SM. Cytogenetic characterization of the golden mussel (Limnoperna fortunei) reveals the absence of sex heteromorphic chromosomes. *Annals of the brazilian academy of sciences*.

26. Marcais G, Kingsford C: Jellyfish: A fast k-mer counter. eagle.fish.washington.edu; https://eagle.fish.washington.edu/whale/fish546/Trinity_r2013-08-14_analysis1-2014-02-08-20-44-13.233/bin/trinityrnaseq_r2013_08_14/trinity-plugins/jellyfish/doc/jellyfish.pdf (2012). Accessed 2023 Jun 9.

27. Ranallo-Benavidez TR, Jaron KS, Schatz MC. GenomeScope 2.0 and Smudgeplot for reference-free profiling of polyploid genomes. *Nat Commun*. nature.com; 2020; doi: 10.1038/s41467-020-14998-3.

28. Cheng H, Concepcion GT, Feng X, Zhang H, Li H. Haplotype-resolved de novo assembly using phased assembly graphs with hifiasm. *Nat Methods*. nature.com; 2021; doi: 10.1038/s41592-020-01056-5.

29. Genomics 10x. longranger: 10x Genomics Linked-Read Alignment, Variant Calling, Phasing, and Structural Variant Calling. Github;

30. Garrison E, Marth G. Haplotype-based variant detection from short-read sequencing. arXiv [q-bio.GN].

31. Zhou C, McCarthy SA, Durbin R. YaHS: yet another Hi-C scaffolding tool. *Bioinformatics*. 2023. doi: 10.1093/bioinformatics/btac808.

32. Howe K, Chow W, Collins J, Pelan S, Pointon D-L, Sims Y, et al.. Significantly improving the quality of genome assemblies through curation. *Gigascience*. 2021; doi: 10.1093/gigascience/giaa153.

33. Cunningham F, Allen JE, Allen J, Alvarez-Jarreta J, Amode MR, Armean IM, et al.. Ensembl 2022. *Nucleic Acids Res*. Oxford Academic; 2021; doi: 10.1093/nar/gkab1049.

34. Uliano-Silva M. MitoHiFi: Find, circularise and annotate mitogenome from PacBio assemblies. Github;

35. Rhie A, McCarthy SA, Fedrigo O, Damas J, Formenti G, Koren S, et al.. Towards complete and error-free genome assemblies of all vertebrate species. *Nature*. nature.com; 2021; doi: 10.1038/s41586-021-03451-0.

36. Baril T, Imrie RM, Hayward A. Earl Grey: a fully automated user-friendly transposable element annotation and analysis pipeline.

37. McCartney MA, Auch B, Kono T, Mallez S, Zhang Y, Obille A, et al.. The genome of the zebra mussel, Dreissena polymorpha: a resource for comparative genomics, invasion genetics, and biocontrol. *G3* . 2022; doi: 10.1093/g3journal/jkab423.

38. Calcino AD, de Oliveira AL, Simakov O, Schwaha T, Zieger E, Wollesen T, et al.. The quagga mussel genome and the evolution of freshwater tolerance. *DNA Res*. Oxford Academic; 2019; doi: 10.1093/dnares/dsz019.

39. Emms DM, Kelly S. OrthoFinder: phylogenetic orthology inference for comparative genomics. *Genome Biol*. Springer; 2019; doi: 10.1186/s13059-019-1832-y.

40. Camacho C, Coulouris G, Avagyan V, Ma N, Papadopoulos J, Bealer K, et al.. BLAST+: architecture and applications. *BMC Bioinformatics*. Springer; 2009; doi: 10.1186/1471-2105-10-421.

41. Buchfink B, Xie C, Huson DH. Fast and sensitive protein alignment using DIAMOND. *Nat Methods*. nature.com; 2015; doi: 10.1038/nmeth.3176.

42. Cantalapiedra CP, Hernández-Plaza A, Letunic I, Bork P, Huerta-Cepas J. eggNOG-mapper v2: Functional Annotation, Orthology Assignments, and Domain Prediction at the Metagenomic Scale. *Mol Biol Evol*. academic.oup.com; 2021; doi: 10.1093/molbev/msab293.

43. Finn RD, Clements J, Eddy SR. HMMER web server: interactive sequence similarity searching. *Nucleic Acids Res*. Oxford University Press; 2011; doi: 10.1093/nar/gkr367.

44. Calcino AD, Kenny NJ, Gerdol M. Single individual structural variant detection uncovers widespread hemizygosity in molluscs. *Philos Trans R Soc Lond B Biol Sci*. 2021; doi: 10.1098/rstb.2020.0153.

45. Thompson JD, Gibson TJ, Higgins DG. Multiple sequence alignment using ClustalW and ClustalX. *Curr Protoc Bioinformatics*. Wiley Online Library; 2002; doi: 10.1002/0471250953.bi0203s00.

46. Capella-Gutiérrez S, Silla-Martínez JM, Gabaldón T. trimAl: a tool for automated alignment trimming in large-scale phylogenetic analyses. *Bioinformatics*. academic.oup.com; 2009; doi: 10.1093/bioinformatics/btp348.

47. Darriba D, Posada D, Kozlov AM, Stamatakis A, Morel B, Flouri T. ModelTest-NG: A New and Scalable Tool for the Selection of DNA and Protein Evolutionary Models. *Mol Biol Evol*. academic.oup.com; 2020; doi: 10.1093/molbev/msz189.

48. Huelsenbeck JP, Ronquist F. MRBAYES: Bayesian inference of phylogenetic trees. *Bioinformatics*. 2001; doi: 10.1093/bioinformatics/17.8.754.

49. Ronquist F, Teslenko M, van der Mark P, Ayres DL, Darling A, Höhna S, et al.. MrBayes 3.2: efficient Bayesian phylogenetic inference and model choice across a large model space. *Syst Biol*. academic.oup.com; 2012; doi: 10.1093/sysbio/sys029.

50. Letunic I, Bork P. Interactive Tree Of Life (iTOL): an online tool for phylogenetic tree display and annotation. *Bioinformatics*. academic.oup.com; 2007; doi: 10.1093/bioinformatics/btl529.

51. Letunic I, Bork P. Interactive Tree Of Life (iTOL) v5: an online tool for phylogenetic tree display and annotation. *Nucleic Acids Res*. academic.oup.com; 2021; doi: 10.1093/nar/gkab301.

52. Afonso LF, Americo JA, Soares-Souza GB, Torres ALQ, Wajsenzon IJR, de Freitas Rebelo M. Gonad transcriptome of golden mussel Limnoperna fortunei reveals potential sex differentiation genes. bioRxiv.

53. Drury DW, Dapper AL, Siniard DJ, Zentner GE, Wade MJ. CRISPR/Cas9 gene drives in genetically variable and nonrandomly mating wild populations. *Sci Adv*. 2017; doi: 10.1126/sciadv.1601910.

54. Korlach J, Gedman G, Kingan SB, Chin C-S, Howard JT, Audet J-N, et al.. De novo PacBio long-read and phased avian genome assemblies correct and add to reference genes generated with intermediate and short reads. *Gigascience*. 2017; doi: 10.1093/gigascience/gix085.

55. Gerdol M, Moreira R, Cruz F, Gómez-Garrido J, Vlasova A, Rosani U, et al.. Massive gene presence-absence variation shapes an open pan-genome in the Mediterranean mussel. *Genome Biol*. 2020; doi: 10.1186/s13059-020-02180-3.

56. Lourenço R, Lopes SS, Saúde L. Left-right function of dmrt2 genes is not conserved between zebrafish and mouse. *PLoS One*. 2010; doi: 10.1371/journal.pone.0014438.

57. Yu J, Wu H, Wen Y, Liu Y, Zhou T, Ni B, et al.. Identification of seven genes essential for male fertility through a genome-wide association study of non-obstructive azoospermia and RNA interference-mediated large-scale functional screening in Drosophila. *Hum Mol Genet*. 2015; doi: 10.1093/hmg/ddu557.

58. Kasahara R, Yuzawa T, Fujii T, Aoki F, Suzuki MG. dmrt11E ortholog is a crucial factor for oogenesis of the domesticated silkworm, Bombyx mori. *Insect Biochem Mol Biol*. 2021; doi: 10.1016/j.ibmb.2020.103517.

59. Shi Y, Wang Q, He M. Molecular identification of dmrt2 and dmrt5 and effect of sex steroids on their expressions in Chlamys nobilis. *Aquaculture*. 2014; doi: 10.1016/j.aquaculture.2014.01.021.

60. Yu F-F, Wang M-F, Zhou L, Gui J-F, Yu X-Y. Molecular Cloning and Expression Characterization of Dmrt2 in Akoya Pearl Oysters, Pinctada martensii. *shre*. National Shellfisheries Association; 2011; doi: 10.2983/035.030.0208.

61. Andersson LS, Larhammar M, Memic F, Wootz H, Schwochow D, Rubin C-J, et al.. Mutations in DMRT3 affect locomotion in horses and spinal circuit function in mice. *Nature*. Nature Publishing Group; 2012; doi: 10.1038/nature11399.

62. Hong C-S, Park B-Y, Saint-Jeannet J-P. The function of Dmrt genes in vertebrate development: it is not just about sex. *Dev Biol*. 2007; doi: 10.1016/j.ydbio.2007.07.035.

63. Yamaguchi A, Lee KH, Fujimoto H, Kadomura K, Yasumoto S, Matsuyama M. Expression of the DMRT gene and its roles in early gonadal development of the Japanese pufferfish Takifugu rubripes. *Comp Biochem Physiol Part D Genomics Proteomics*. 2006; doi: 10.1016/j.cbd.2005.08.003.

64. Ratié L, Desmaris E, García-Moreno F, Hoerder-Suabedissen A, Kelman A, Theil T, et al.. Loss of Dmrt5 Affects the Formation of the Subplate and Early Corticogenesis. *Cereb Cortex*. 2020; doi: 10.1093/cercor/bhz310.

65. Graf M, Teo Qi-Wen E-R, Sarusie MV, Rajaei F, Winkler C. Dmrt5 controls corticotrope and gonadotrope differentiation in the zebrafish pituitary. *Mol Endocrinol*. 2015; doi: 10.1210/me.2014-1176.

66. Urquhart JE, Beaman G, Byers H, Roberts NA, Chervinsky E, O'Sullivan J, et al.. DMRTA2 (DMRT5) is mutated in a novel cortical brain malformation. *Clin Genet*. 2016; doi: 10.1111/cge.12734.

67. Kasahara R, Aoki F, Suzuki MG. Deficiency in dmrt99B ortholog causes behavioral abnormalities in the silkworm, Bombyx mori. *Appl Entomol Zool* . 2018; doi: 10.1007/s13355-018-0569-5.

68. Zwarts L, Vanden Broeck L, Cappuyns E, Ayroles JF, Magwire MM, Vulsteke V, et al.. The genetic basis of natural variation in mushroom body size in Drosophila melanogaster. *Nat Commun*. Nature Publishing Group; 2015; doi: 10.1038/ncomms10115.

69. Huang S, Ye L, Chen H. Sex determination and maintenance: the role of DMRT1 and FOXL2. *Asian J Androl*. 2017; doi: 10.4103/1008-682X.194420.

70. Erdman SE, Burtis KC. The Drosophila doublesex proteins share a novel zinc finger related DNA binding domain. *EMBO J*. 1993; doi: 10.1002/j.1460-2075.1993.tb05684.x.

71. Mawaribuchi S, Ito Y, Ito M. Independent evolution for sex determination and differentiation in the DMRT family in animals. *Biol Open*. 2019; doi: 10.1242/bio.041962.

72. Li J, Zhou Y, Zhou Z, Lin C, Wei J, Qin Y, et al.. Comparative transcriptome analysis of three gonadal development stages reveals potential genes involved in gametogenesis of the fluted giant clam (Tridacna squamosa). *BMC Genomics*. 2020; doi: 10.1186/s12864-020-07276-5.

73. Sun D, Yu H, Li Q. Examination of the roles of Foxl2 and Dmrt1 in sex differentiation and gonadal development of oysters by using RNA interference. *Aquaculture*. 2022; doi: 10.1016/j.aquaculture.2021.737732.

74. Rodinho Nunes Ferreira JG, Americo JA, A S do Amaral DL, Sendim F, da Cunha YR, Tree of Life Programme, et al.. Supporting data for "A chromosome-level assembly supports genome-wide investigation of the DMRT gene family in the golden mussel (Limnoperna fortunei)" *GigaScience* Database. 2023. http://dx.doi.org/10.5524/102411

75. Tree of Life. Tree of Life Programme author list. doi: 10.5281/zenodo.8027160

Figure 1 Click here to access/download;Figure;Figure1.png

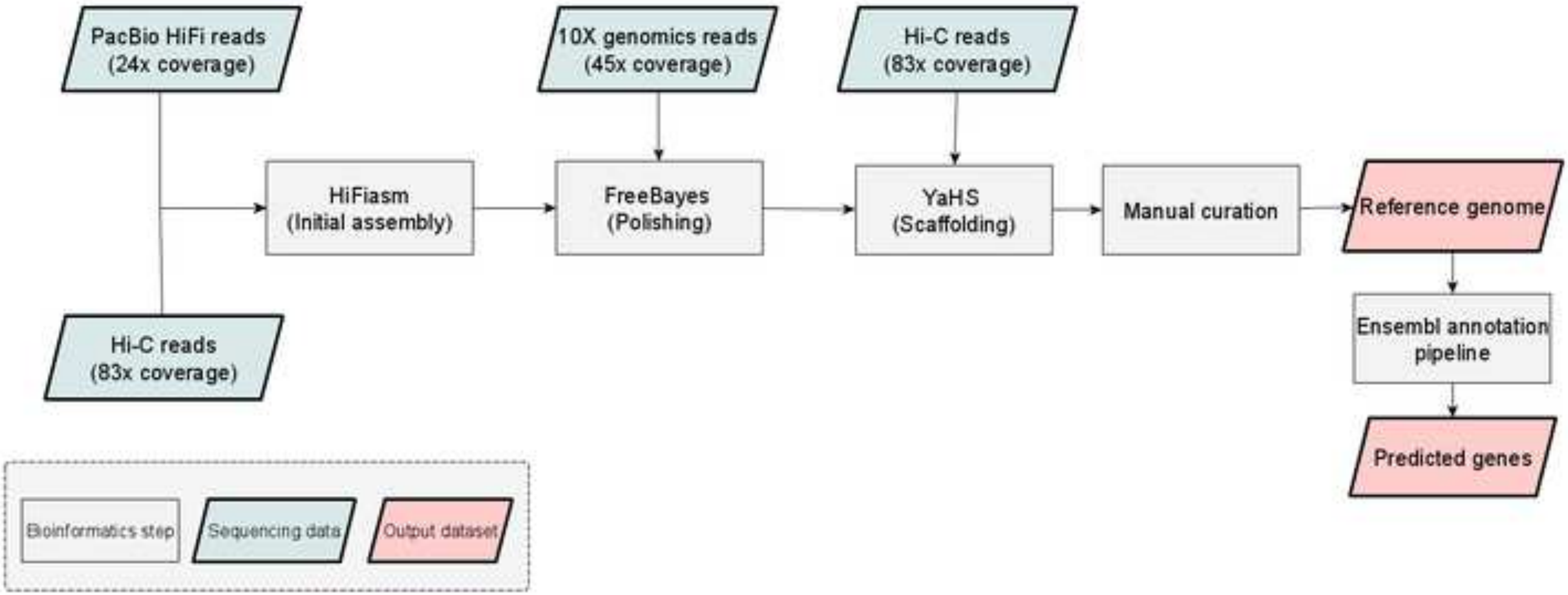

Figure 2

Click here to access/download;Figure;Figure2.png

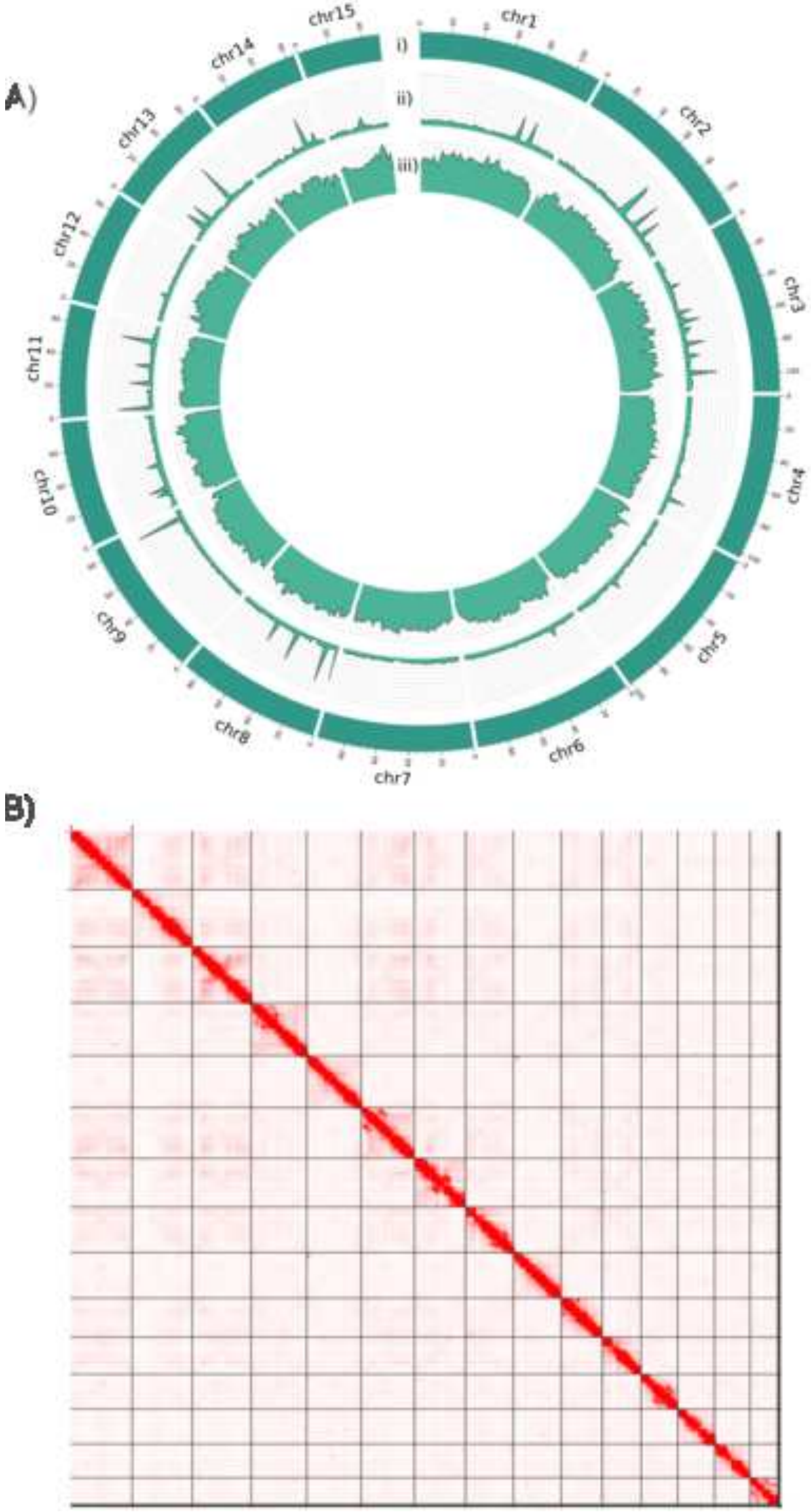

Figure 3

Click here to access/download;Figure;Figure3.png

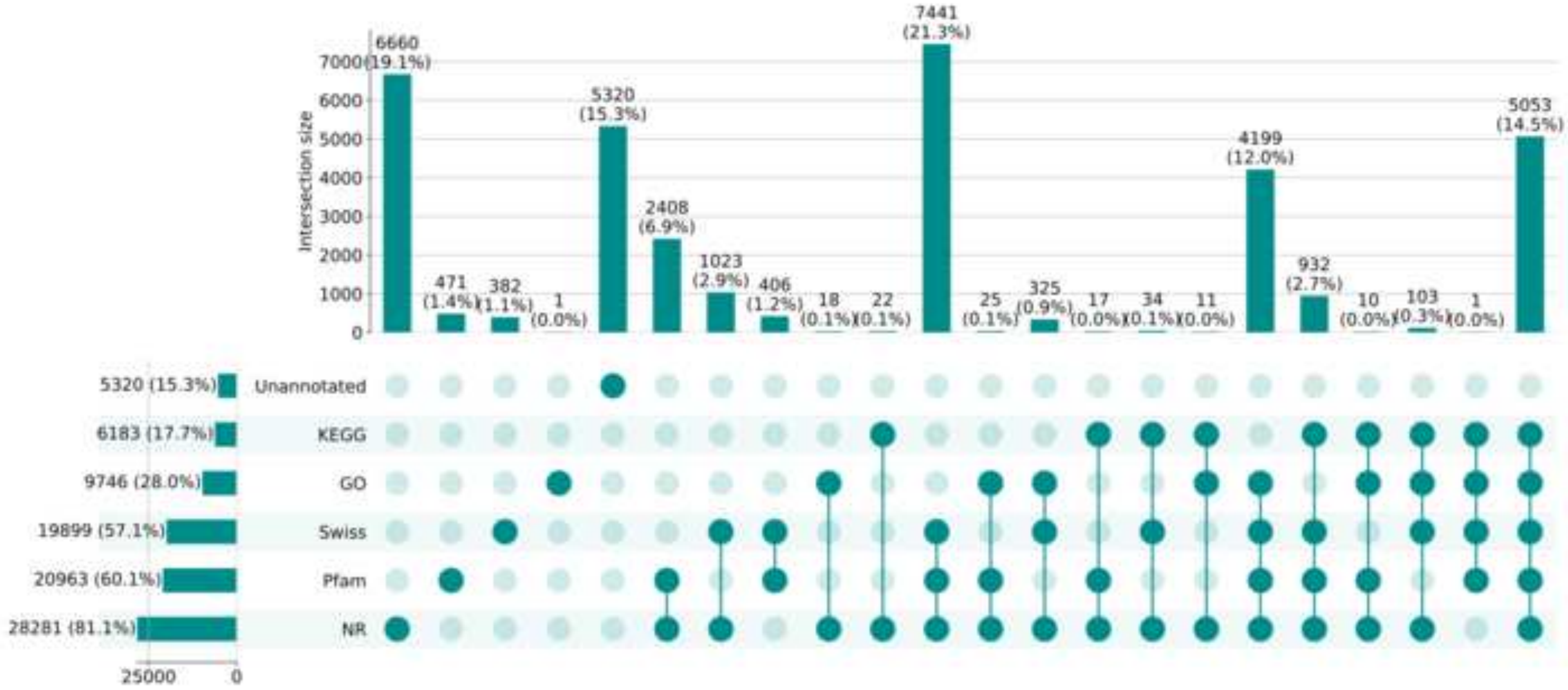

Figure 4 Click here to access/download;Figure;Figure4.png

A)

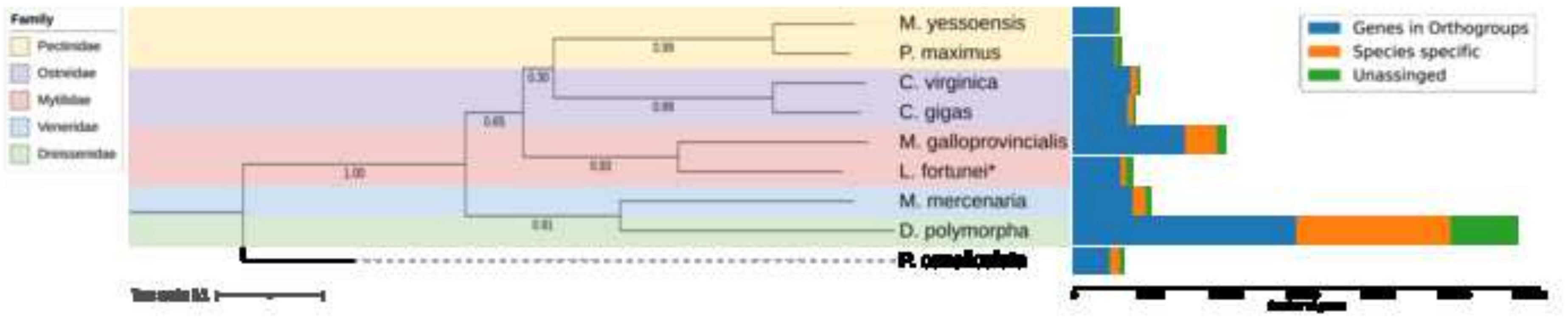

B)

	C. gigas	C. virginica	D. polymorpha	L. fortunei	M. galloprovincialis	M. mercenaria	M. yessoensis	P. canaliculata	P. maximus
C. gigas									
C. virginica	14545								
D. polymorpha	11144	10821							
L. fortunei	11889	11547	11669						
M. galloprovincialis	11738	11359	12167	14411					
M. mercenaria	11004	10661	13214	11192	11253				
M. yessoensis	11802	11489	11251	11846	11667	11047			
P. canaliculata	10183	9963	10113	10274	9926	9958	10350		
P. maximus	11892	11546	11306	11929	11739	11142	14548	10346	

Figure 5

Click here to access/download;Figure;Figure5.png

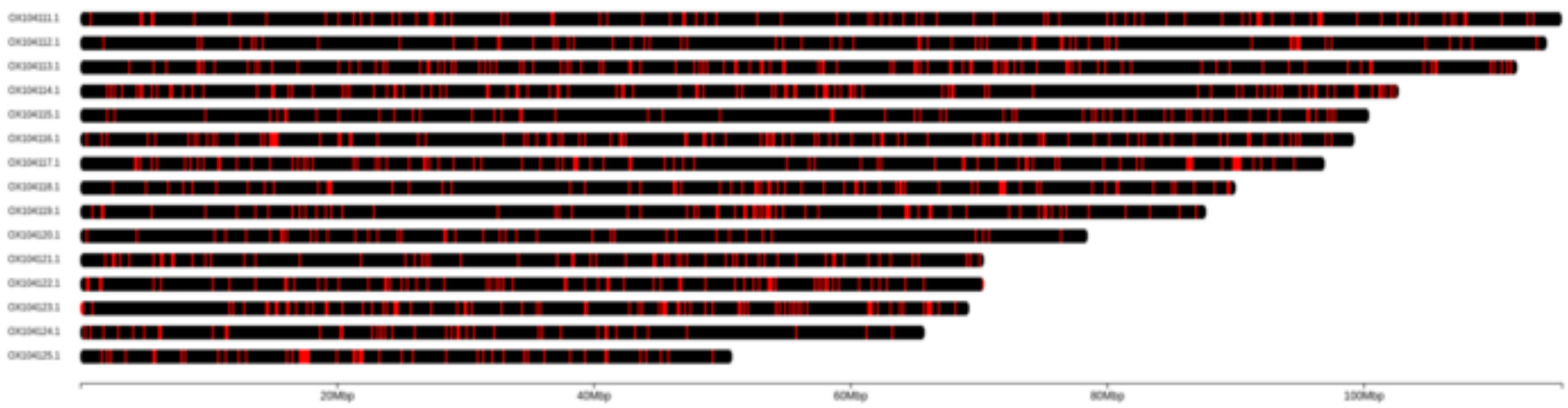

Figure 6

Click here to access/download;Figure;Figure6.png

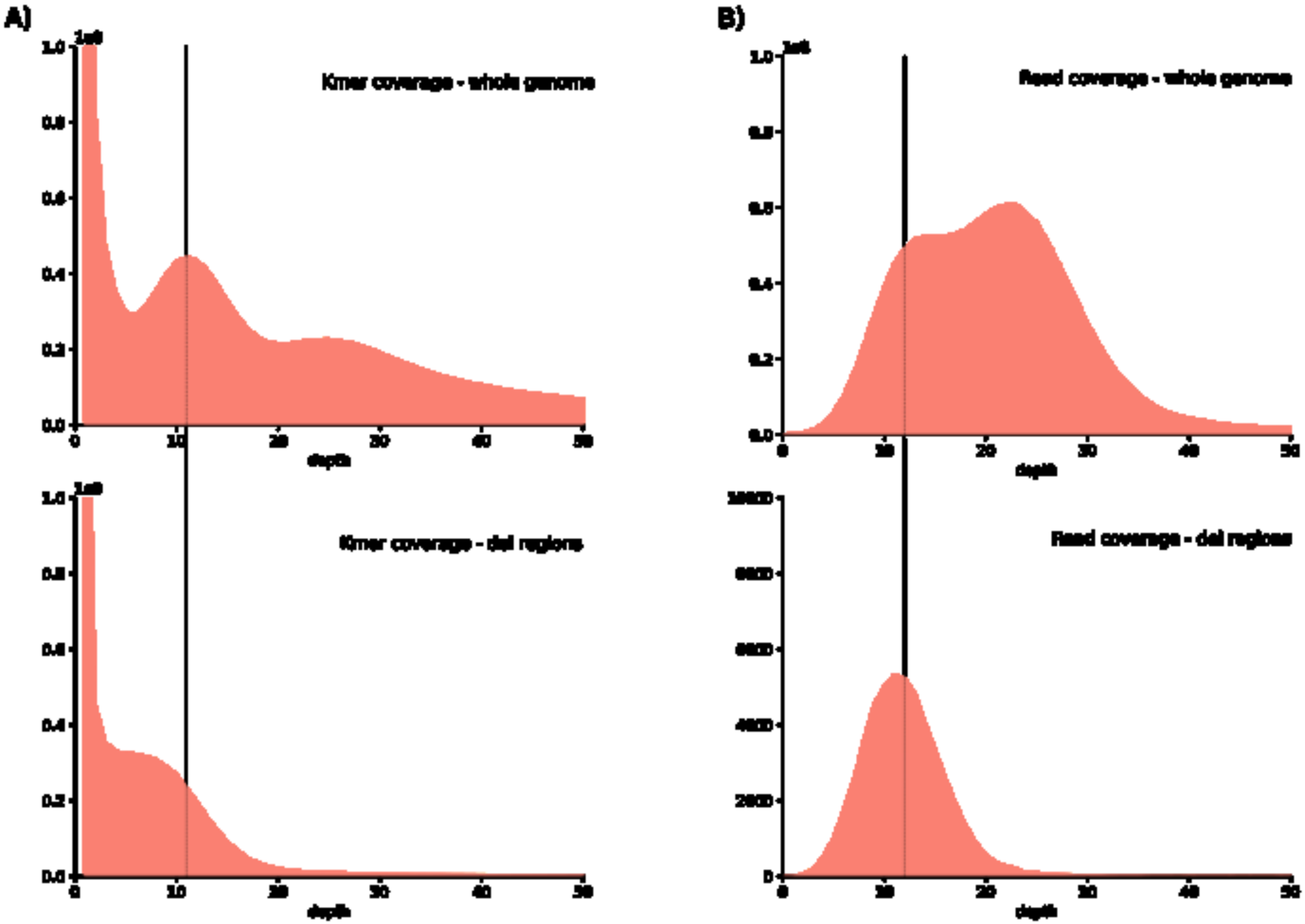

Figure 7

Click here to access/download;Figure;Figure7.png

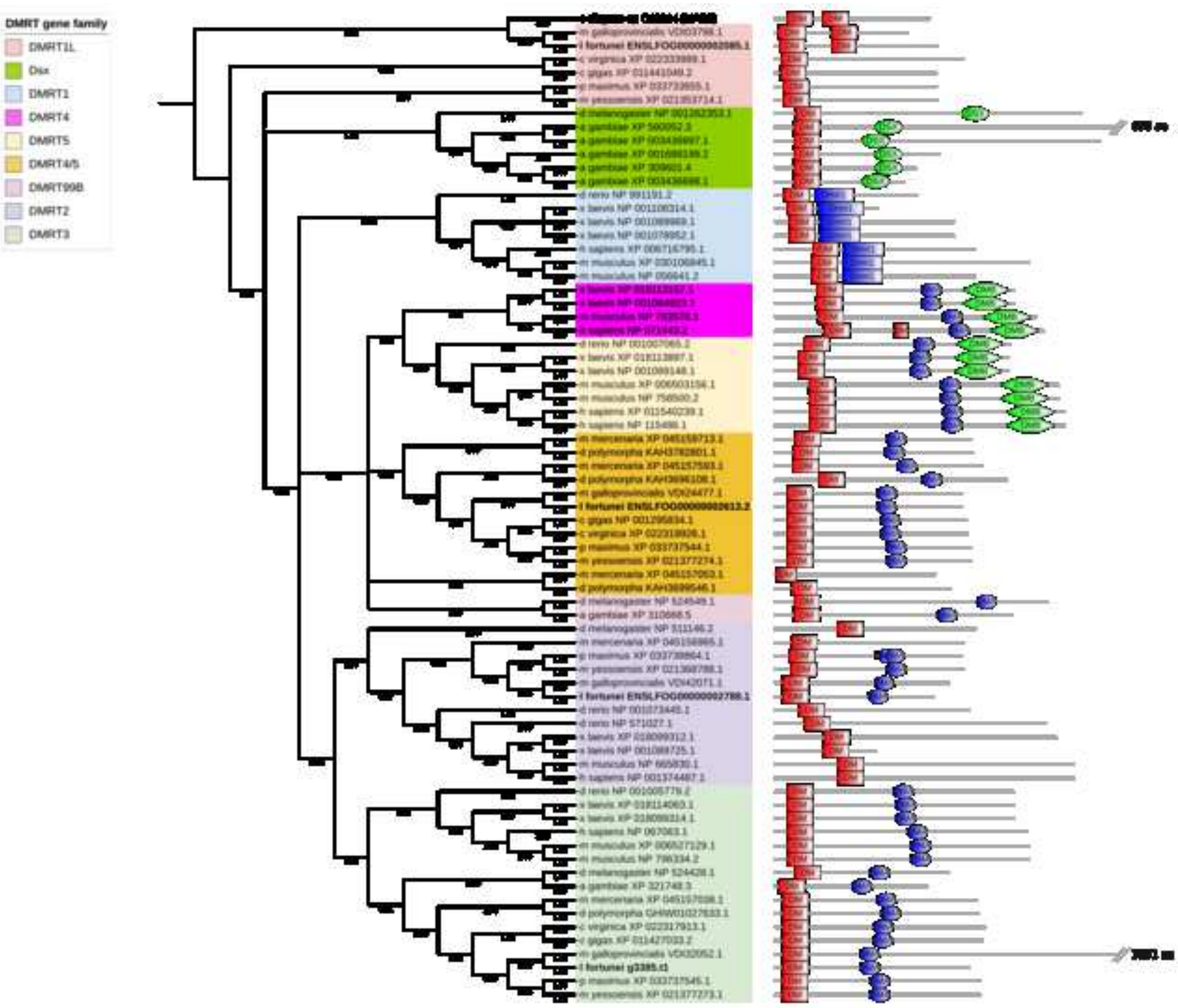

Supplementary material

Click here to access/download
Supplementary Material
Supplementary material.docx

Click here to access/download

Supplementary Material

Supplementary Data Note 1.docx

Supplementary Data Note 2

Click here to access/download
Supplementary Material
Supplementary Data Note 2.docx

Supplementary Data Note 3

Click here to access/download
Supplementary Material
Supplementary Data Note 3.docx

Cover letter Click here to access/download;Personal Cover;cover_letter.docx

Dear Editor-in-Chief,

We are enclosing herewith a manuscript by João G. R. N. Ferreira, Juliana A. Americo, Danielle L. A. S. do Amaral, Fábio Sendim, Yasmin R. da Cunha, Tree of Life Programme, Mark Blaxter, Marcela Uliano-Silva and Mauro de F. Rebelo entitled “A chromosome-level genome supports genome-wide investigation of the DMRT gene family in the golden mussel (*Limnoperna fortunei*)” submitted to GigaScience for possible evaluation. The type of submitted manuscript is Data Note.

The golden mussel is an Asian freshwater bivalve that was introduced in South America almost 30 years ago and since then has spread across the continent, causing both economic and environmental impacts. Traditional control strategies haven’t been able to stop dispersal of the golden mussel, and our group proposes a biotechnology solution based on *gene drive*. A *gene drive* solution for population control of the malaria mosquito has been showing promising results targeting genes involved in sex differentiation, however little is known about sex differentiation in the golden mussel. In addition to that, the current reference genome is highly fragmented, hindering its applications not only for the planning of genome editing experiments but also for the study of the molecular biology of the species as a whole.

In this study, we have developed a new, high-quality reference genome for the golden mussel. The assembly was achieved using a combination of PacBio HiFi, Hi-C and 10X sequencing data, and 99.4% of its sequence is distributed over the 15 largest scaffolds, that putatively represent the 15 chromosomes of the species. Based on the chromosome-level genome we detected widespread hemizygosity in all chromosomes. We have also done a genome-wide characterization of the DMRT gene family, identifying four DMRT genes in the golden mussel. One of those genes (DMRT1L) is assumed to play a role in sex determination and differentiation and therefore represents a potential target for biotechnology based control strategies. The new genome and the findings of this study are expected to support both basic and applied research on this invasive species.

The authors of this study declare no competing interests and all authors have approved the manuscript for submission. The content of the manuscript has not been published or submitted for publication elsewhere, although a preprint in a Genome Announcement format (i.e., containing none of the downstream analyzes) has been published at bioRxiv (doi: https://doi.org/10.1101/2022.09.29.509984)

Sincerely,

Juliana Alves Americo

Universidade Federal do Rio de Janeiro, Instituto de Biofísica Carlos Chagas Filho, Centro de Ciências da Saúde. Av. Carlos Chagas Filho, 373. Bloco G - Sala G2-050 - Cidade Universitária CEP: 21941-902 - Rio de Janeiro - RJ
