# Supplementary material for: A chromosome-level assembly supports genome-wide investigation of the DMRT gene family in the golden mussel (Limnoperna fortunei)
Source: Gigascience. 2023 Sep 30;12:giad072. doi: 10.1093/gigascience/giad072 (PMC10541798; doi:10.1093/gigascience/giad072)
Supplement: giad072_Supplemental_Files [file giad072_supplemental_files.zip › Supplementary Data Note 1.docx]

# Supplementary Data Note 1

The rate of mapping of RNA-seq data against the gene models from the previous draft genome and the chromosome-level genome annotations were compared (Table 1).

**Table 1.** Statistics of RNA-seq mapping against gene models from the draft and from the chromosome-level genome.

| **Statistic (%)** | Draft  (GCA_003130415.1) | Chromosome-level (GCA_944474755.1) |
| --- | --- | --- |
| *Uniquely mapped reads* | 40.02 | 70.85 |
| Reads mapped to multiple loci | 8.69 | 8.23 |
| Reads mapped to too many loci | 0.10 | 0.73 |
| Reads unmapped: too many mismatches | 0.00 | 0.00 |
| Reads unmapped: too short | 51.17 | 19.89 |
| Reads unmapped: other | 0.02 | 0.30 |
| Chimeric reads | 0.00 | 0.00 |

**Commands run for the mapping:**

# trimming reads to remove lower quality end

trimmomatic PE -threads 12 SRR5188384_1.fastq SRR5188384_2.fastq SRR5188384_1.paired.fastq SRR5188384_1.unpaired.fastq SRR5188384_2.paired.fastq SRR5188384_2.unpaired.fastq CROP:135

# generate reference directory for chromosome-level gene models (Limnoperna_fortunei_gca944474755v1.xbLimFort5.1.genes.fna.starDir)
STAR --runMode genomeGenerate --runThreadN 8 --genomeSAindexNbases 13 --genomeDir Limnoperna_fortunei_gca944474755v1.xbLimFort5.1.genes.fna.starDir --genomeFastaFiles Limnoperna_fortunei_gca944474755v1.xbLimFort5.1.genes.fna

# generate reference directory for draft genome gene models

STAR --runMode genomeGenerate --runThreadN 8 --limitGenomeGenerateRAM 42444259594 --genomeSAindexNbases 11 --genomeDir lfortunei_CDS.fasta.starDir --genomeFastaFiles lfortunei_CDS.fasta

# align RNA-seq reads against the genome CDS, adjusting –outFilterScoreMinOverLread and –outFilterMatchNminOverLread to relax stringency of required mapped length

STAR --limitBAMsortRAM 22608676932 --runMode alignReads --runThreadN 14 --outFilterScoreMinOverLread 0.3 --outFilterMatchNminOverLread 0.3 --genomeDir Limnoperna_fortunei_gca944474755v1.xbLimFort5.1.genes.fna.starDir --readFilesIn SRR5188384_1.paired.fastq SRR5188384_2.paired.fastq --outFileNamePrefix RNAseq-STAR. --outSAMtype BAM SortedByCoordinate

# align RNA-seq reads against the draft genome gene models, adjusting –outFilterScoreMinOverLread and –outFilterMatchNminOverLread to relax stringency of required mapped length

STAR --limitBAMsortRAM 22608676932 --runMode alignReads --runThreadN 14 --outFilterScoreMinOverLread 0.3 --outFilterMatchNminOverLread 0.3 --genomeDir Limnoperna_fortunei_gca944474755v1.xbLimFort5.1.genes.fna.starDir --readFilesIn SRR5188384_1.paired.fastq SRR5188384_2.paired.fastq --outFileNamePrefix RNAseq-STAR. --outSAMtype BAM SortedByCoordinate
