# Supplementary material for: A chromosome-level assembly supports genome-wide investigation of the DMRT gene family in the golden mussel (Limnoperna fortunei)
Source: Gigascience. 2023 Sep 30;12:giad072. doi: 10.1093/gigascience/giad072 (PMC10541798; doi:10.1093/gigascience/giad072)
Supplement: giad072_Supplemental_Files [file giad072_supplemental_files.zip › Supplementary Data Note 2.docx]

# Supplementary Data Note 2

### Hemizygosity identification

The identification of the hemizygous regions was done using an adapted version of the protocol reported by Calcino et al., 2020 at the Supplementary material (“Command line arguments from Single individual structural variant detection uncovers widespread hemizygosity in molluscs”).

The following steps of the protocol were run, with the modified steps in bold:

#Find tandem repeats

trf genome.fa 2 7 7 80 10 50 500 -d -h

#Convert trf output to bed

TRFdat_to_bed.py --dat genome.fa.2.7.7.80.10.50.500_mod.dat --bed genome.fa.2.7.7.80.10.50.500.bed

#Convert bed to zero based six field bed with arbitrary sixth field

awk '{print $1"\t"$2-1"\t"$3"\t"$4}' genome.fa.2.7.7.80.10.50.500.bed | sed 's/Sequence://' | awk '{print $0"\t"$3-$2"\t1"}' >genome.fa.2.7.7.80.10.50.500_0based_6field.bed

**#Run pbmm2**

pbmm2 align -j 18 genome.fa pacbio_reads.fofn genome.aligned.bam --sort --preset HIFI --sample sample1

**#Run pbsv**

pbsv discover --hifi --tandem-repeats genome.fa.2.7.7.80.10.50.500_0based_6field.bed genome.aligned.bam genome.svsig.gz

pbsv call --hifi -j 16 genome.fa genome.svsig.gz genome.var.vcf

#Extract DELs that are not homozygous for the alternative allele (possible genome assembly errors) as a zero based six field bed file

grep DEL genome.var.vcf | grep PASS | grep -v '1/1' | awk '{print $1"\t"$2-1"\t"$2+length($4)-1"\t"$3}' | awk '{print $0"\t"$3-$2"\t1"}' >genome.var.DEL.6field.bed

#Extract INSs that are not homozygous for the alternative allele (possible misannotation) as a zero based six field bed file

grep INS genome.var.vcf | grep PASS | grep -v -P '1/1' | awk '{print $1"\t"$2-1"\t"$2+length($5)-1"\t"$3}' | awk '{print $0"\t"$3-$2"\t1"}' >genome.var.INS.6field.bed

### chromoMap

The command to plot the chromoMap was modified to make the vertical lines representing the hemizygous regions thinner and therefore the final aspect of the map more realistic to the 6% proportion of hemizygosity of the genome.

#Generate chromosome file for chromoMap. The head value equals the number of chromosomal scaffolds in the genome.fa file

bioawk -c fastx '{ print $name, length($seq) }' < genome.fa | head -15 | awk '{print $1"\t1\t"$2}' >genome_chrom.txt

#Make feature file for chromoMap of the >10k deletions that are not annotated as tandem repeats

grep DEL genome.var.vcf | grep PASS | grep -v '1/1' | grep -v TANDEM | awk '{print $1"\t"$2-1"\t"$2+length($4)-1"\t"$3}' | awk '{print $0"\t"$3-$2"\t1"}' | awk '$5>9999' | awk '{print $4"\t"$1"\t"$2"\t"$3}' >genome_dels.txt

**#Run chromoMap in R**

chromoMap("genome_chrom.txt","genome_dels.txt",left_margin = 100, n_win.factor = 8, anno_col = "red", chr_length = 2)

### Kmer and read coverage analysis

The k-mer and read coverage plots built as additional evidence of hemizygosity were also built according to Calcino et al., 2020 protocol.

#### Kmer count

The section “2. Kmer analysis of hemizygous regions” was run to build the k-mer coverage plot of reads that mapped to i) the whole genome; and ii) to the hemizygous regions (more specifically to the deletions). For the whole genome plot, no significant change needed to be applied except skipping the first three steps where the original protocol mapped short reads to the genome and our protocol starts from the PacBio HiFi mapping (genome.aligned.bam) previously done using “pbmm2 align”.

# i) Kmer counts for reads mapped anywhere in the genome (whole_genome)

## Get mapped reads in fasta format

samtools view -@ 8 -F 4 -h genome.aligned.bam >all_mapping.sam

reformat.sh in=all_mapping.sam out=all_mapping.fa

## Run jellyfish on all mapped reads

jellyfish count -t 8 -C -m 21 -s 16G all_mapping.fa -o all_reads.jf

jellyfish histo -o all_reads.histo all_reads.jf

For the hemizygous plot, adaptations were needed. The original protocol used “bedmap” to select only the reads whose length was 100% within an hemizygous region. However, we were working with long reads most of which had a length higher than the hemizygous region sizes. Therefore, we first selected reads mapping to large (>=100bp) hemizygous regions (using “bedtools intersect”) and then using an *in-house* script (exclude_large_del_reads.py) we filtered the dataset to eliminate reads that mapped to the hemizygous region with a large deletion/clipping (and therefore probably came from the haplotype that did not have that region). The *in-house* script is available at <https://github.com/jgnunes/golden_mussel_genome/blob/main/scripts/exclude_large_del_reads.py>.

# ii) Kmer counts for reads mapped to hemizygous deletion regions (del_regions)

**## Selecting hemizygous deletions larger than or equal to 100 bp**

awk '$5>99' genome.var.DEL.6field.bed > genome.var.DEL.6field.gt-99.bed

**## Getting reads that map to the hemizygous deletion regions**

bedtools intersect -wa -u -a genome.aligned.bam -b genome.var.DEL.6field.gt-99.bed > 99-deletionsHiFi.bam

**## Exclude reads from haplotype containing deletion and save new dataset to file 99-deletionsHiFi.filtered.bam**

python exclude_large_del_reads.py 99-deletionsHiFi.bam

## Convert filtered reads to FASTA format

reformat.sh in=99-deletionsHiFi.filtered.bam out=del_reads.fa

## Count kmers using jellyfish

jellyfish count -t 8 -C -m 21 -s 16G del_reads.fa -o del_reads.jf

## Generate histogram from kmers count

jellyfish histo -o del_reads.histo del_reads.jf

#### Read coverage

The protocols “4. Read coverage of hemizygous regions” and “5. Read coverage of genome with sliding window” were used to build the read coverage plots of the i) hemizygous regions and ii) the whole genome, respectively.

For the read coverage over the hemizygous regions, since we already had a BAM file containing the reads mapping to the hemizygous regions we were able to skip the first 4 steps from “4. Read coverage of hemizygous regions”, starting from the mosdepth step:

# ii) Read coverage over the hemizygous deletion regions

## Run mosdepth on selected deletions

mosdepth -t 8 -m -b genome.var.DEL.6field.gt-99.bed genome 99-deletionsHiFi.filtered.bam

## Count how many deletions have each level of coverage

zcat genome.regions.bed.gz | awk 'BEGIN{OFS=FS="\t"}{$6=sprintf("%.0f",$5) }1' | cut -f6 | sort -n | uniq -c | sed -e 's/^ *//' -e 's/\ /\t/' | awk '{print $2"\t"$1}' | head -200 >deletion_coverage.txt

No modification was needed in the original protocol to calculate the whole genome read coverage:

# i) Read coverage over the whole genome

## Run genomeCoverageBed to calculate coverage at every position in the genomegenomeCoverageBed -d -ibam genome.aligned.bam >genome.cov

## Split coverage file by scaffold/chromosome

awk '{print>$1".cov"}' genome.cov

## Calculate the median coverage of every 1000 bp window with median_sliding_window.gawk script

for i in *cov ; do cat $i | sh median_sliding_window.gawk | sed 's/\ /\t/g' >`echo $i | sed 's/cov/median/'` ; done

#count how many windows have each coverage value

cat *median | awk 'BEGIN{OFS=FS="\t"}{$4=sprintf("%.0f",$3) }1' | cut -f4 | sort -n | uniq -c | sed -e 's/^ *//' -e 's/\ /\t/' | awk '{print $2"\t"$1}' >coverage_count.txt

### Reference:

Calcino, Andrew D.; Kenny, Nathan J.; Gerdol, Marco (2021): Supplementary material from "Single individual structural variant detection uncovers widespread hemizygosity in molluscs". The Royal Society. Collection. <https://doi.org/10.6084/m9.figshare.c.5324903.v1>
