# Supplementary material for: A chromosome-level assembly supports genome-wide investigation of the DMRT gene family in the golden mussel (Limnoperna fortunei)
Source: Gigascience. 2023 Sep 30;12:giad072. doi: 10.1093/gigascience/giad072 (PMC10541798; doi:10.1093/gigascience/giad072)
Supplement: giad072_Supplemental_Files [file giad072_supplemental_files.zip › Supplementary Data Note 3.docx]

# Supplementary data note 3

The original dataset of DMRT genes was inspected before the inference of the final phylogenetic tree. Below, we describe the manual alterations that were implemented:

DMRT3 genes were found in single copy in all species but *Dreissena polymorpha*, where two putative DMRT3 genes were found (KAH3721156.1 and KAH3721157.1). However, KAH3721156.1 was missing the DMA domain and KAH3721157.1 was missing the DM domain. Those two sequences were shown to be neighbors on chromosome 13 (NC_068367.1) separated by a distance of 880 bp, therefore it was likely that they were incorrectly split during annotation. That hypothesis was confirmed after we searched the publicly available transcriptome GHIW00000000.1 and found a transcript (GHIW01027633.1) that covered both genes. Therefore, the partial gene models (KAH3721156.1 and KAH3721157.1) were replaced by the complete one.

*C. virginica* also had two DMRT1L genes (XP_022333988.1 and XP_022333989.1). However, a manual inspection has shown that the 2 genes mapped to the same loci in the *C. virginica* genome (location: LOC111130972), i.e., they are actually isoforms from the same gene that OrthoFinder’s script to remove isoform duplicates was not able to detect. Therefore, only the longest isoform (XP_022333989.1) was kept in the dataset.
