# Supplementary material for: A chromosome-level assembly supports genome-wide investigation of the DMRT gene family in the golden mussel (Limnoperna fortunei)
Source: Gigascience. 2023 Sep 30;12:giad072. doi: 10.1093/gigascience/giad072 (PMC10541798; doi:10.1093/gigascience/giad072)
Supplement: giad072_Supplemental_Files [file giad072_supplemental_files.zip › Supplementary material.docx]

# Supplementary materials


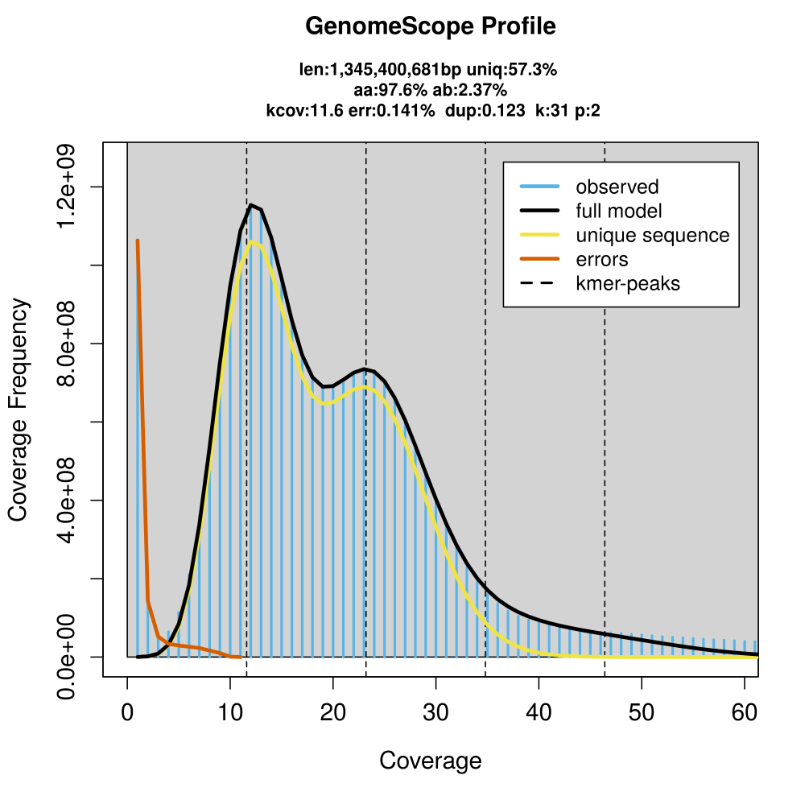


**Supplementary Figure S1.** GenomeScope profile built from PacBio HiFi data.

len = assembly length. uniq = percent of genome which is unique (i.e. non-repetitive); aa = homozygosity rate; ab = heterozygosity rate; kcov = kmer coverage at heterozygous peak; err = error rate of reads; dup = rate of reads duplications; k = kmer size; p = ploidy.


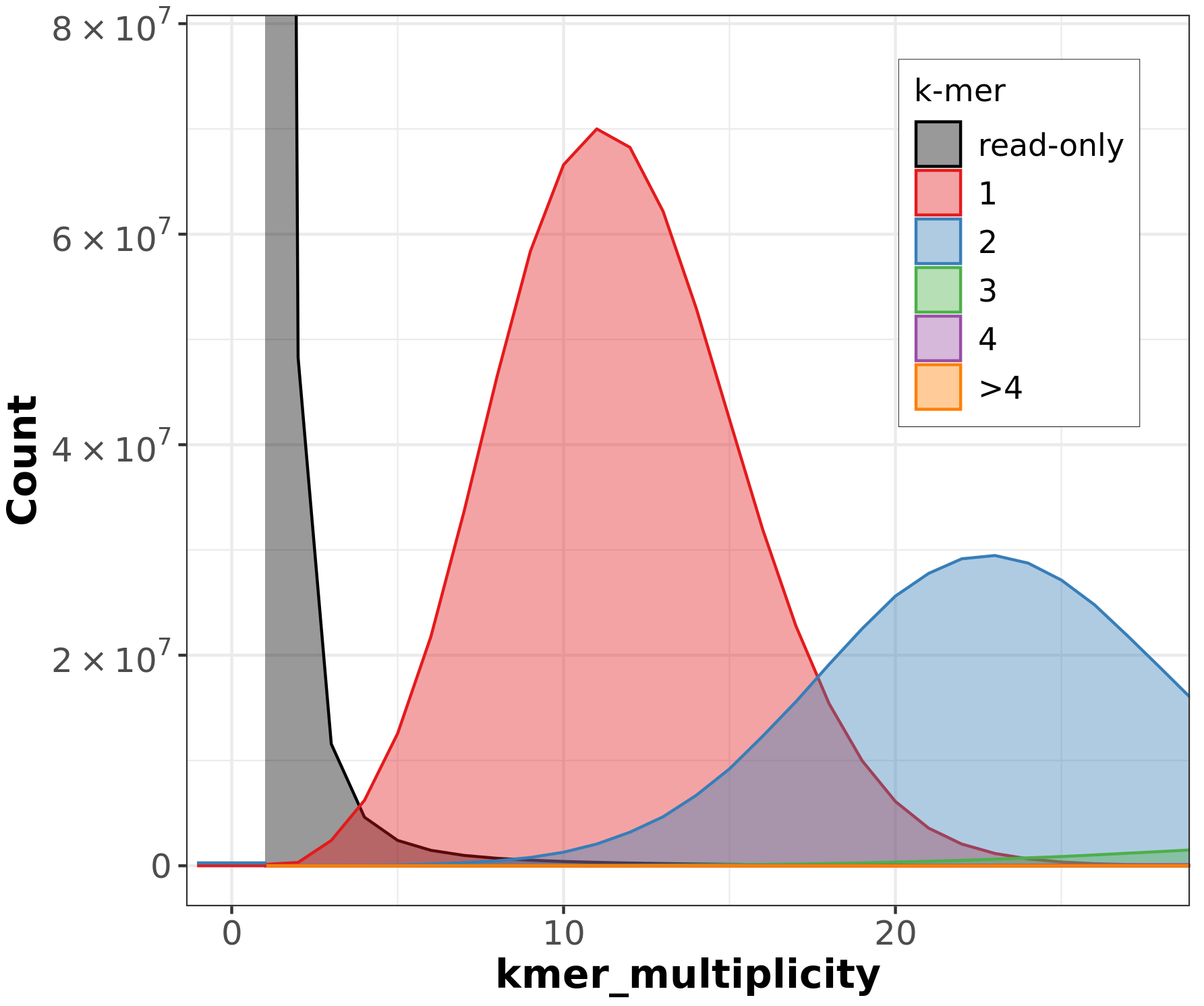

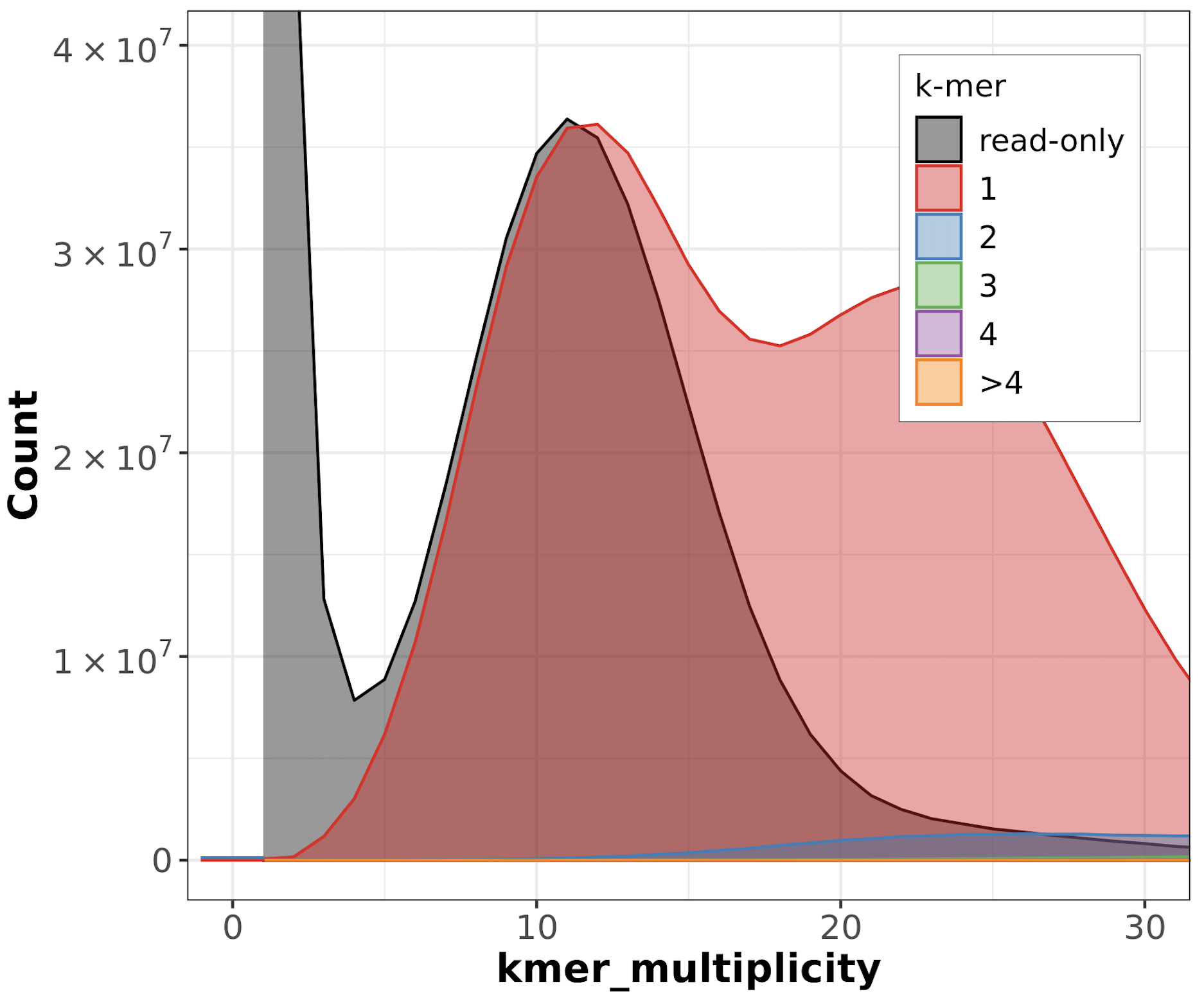
**Supplementary Figure S2.** Merqury plots for (A) the chromossome-level genome and (B) the chromosome-level genome concatenated with its haplotigs (GCA_944589985.1). The “read only” peak in (A) at kmer-multiplicity ~10 represents k-mers that were not found in the primary assembly due to the heterozygous regions. (B) shows kmers found on both haplotypes, the homozygous ones repeated twice (blue color, homozygous peak) and heterozygous only once (red color, heterozygous peak). The grey area in (b) comprises only the erroneous kmers (low frequency) demonstrating the completeness of both assembled haplotypes.

**Supplementary table S1.** General statistics for the golden mussel genome sequence.

**COMPOSITION**     A = 442398358 (33.1%),

C = 224258371 (16.8%),

G = 224472855 (16.8%),

T = 444124758 (33.3%),

N = 305617 (0.0%),

CpG = 52146080 (3.9%)

**SCAFFOLD**      sum = 1335559959,

n = 309,

mean = 4322200.51456311,

largest = 115440917,

smallest = 1000

SCAFFOLD       N50 = 97051362, L50 = 7

SCAFFOLD       N60 = 89737950, L60 = 8

SCAFFOLD       N70 = 78518013, L70 = 10

SCAFFOLD       N80 = 70815894, L80 = 11

SCAFFOLD       N90 = 69091614, L90 = 13

SCAFFOLD       N100 = 1000, L100 = 309

**CONTIG** sum = 1335254342,

n = 1838,

mean = 726471.350380849,

largest = 8 315 943,

smallest = 273

CONTIG  N50 = 1498882, L50 = 277

CONTIG  N60 = 1221040, L60 = 376

CONTIG  N70 = 962899, L70 = 500

CONTIG  N80 = 718770, L80 = 662

CONTIG  N90 = 441659, L90 = 895

CONTIG  N100 = 273, L100 = 1838

**GAP**     sum = 305617,

n = 1529,

mean = 199.880313930674,

largest = 200,

smallest = 17

###

**Supplementary table S2.** Run accessions for the RNA-seq dataset used to support the gene prediction for the chromosome-level genome.

| **Run accession(s)** | ***Study accession: title*** |
| --- | --- |
| ERR9503467 | ERP136725: *Limnoperna fortunei* (golden mussel), genomic and transcriptomic data |
| SRR5188384 | SRP096882: *Limnoperna fortunei* Genome sequencing and assembly |
| SRR11685441-461 | SRP260041: Transcriptome sequencing of golden mussel foot exposed to magnetic nanoparticles |

**Supplementary table S3.** Number of proteins associated with each gene.

| **Number of proteins per gene** | **Number of genes** |
| --- | --- |
| 1 | 18 647 |
| 2 | 7 597 |
| 3 | 4 150 |
| 4 | 2 210 |
| 5 | 1 124 |
| 6 | 535 |
| 7 | 281 |
| 8 | 131 |
| 9 | 89 |
| 10 | 31 |
| 11 | 36 |
| 12 | 15 |
| 13 | 7 |
| 14 | 4 |
| 15 | 2 |
| 19 | 2 |
| 21 | 1 |

**Supplementary table S4.** BUSCO statistics of gene models from the draft and from the chromosome-level genome assembly.

| ***Assembly*** | **BUSCO (metazoa_odb10)** |
| --- | --- |
| Draft (GCA_003130415.1) | C:41.8%[S:37.2%,D:4.6%],F:25.2%,M:33.0%,n:954 |
| Chromosome-level (GCA_944474755.1) | C:94.9%[S:94.1%,D:0.8%],F:2.2%,M:2.9%,n:954 |

**Supplementary table S5.** Mollusk species selected for the OrthoFinder analysis.

| ***Scientific name* (common name)** | **Accession** |
| --- | --- |
| *Crassostrea gigas* (Pacific oyster) | GCA_011032805.1 |
| *Crassostrea virginica* (Eastern oyster) | GCA_002022765.4 |
| *Dreissena polymorpha* (Zebra mussel) | GCA_020536995.1 |
| ***Limnoperna fortunei* (Golden mussel)** | GCA_944474755.1 |
| *Mercenaria mercenaria* (Hard clam) | GCA_014805675.2 |
| *Pecten maximus* (Great scallop) | GCA_902652985.1 |
| *Pomacea canaliculata* (Golden apple snail) | GCF_003073045.1 |
| *Mizuhopecten yessoensis* (Japanese scallop) | GCA_002113885.1 |
| *Mytilus galloprovincialis* (Mediterranean mussel) | GCA_900618805.1 |

**Supplementary table S6.** Overall OrthoFinder statistics.

| ***Statistics*** | **Value** |
| --- | --- |
| Number of species | 9 |
| Number of genes | 502269 |
| Number of genes in orthogroups | 436439 |
| *Number of unassigned genes* | 65830 |
| Number of orthogroups | 50219 |
| Number of species-specific orthogroups | 24095 |
| Number of genes in species-specific orthogroups | 150662 |
| Mean orthogroup size | 8.7 |
| Median orthogroup size | 4.0 |
| *Number of orthogroups with all species present* | 7616 |
| *Number of single-copy orthogroups* | 777 |

**Supplementary table S7.** Per species OrthoFinder statistics.

|  | ***C.  Gigas*** | ***C. Virginica*** | ***D. Polymor-pha*** | ***L. Fortunei*** | ***M. Gallopro-vincialis*** | ***M. Mercena-ria*** | ***M. Yessoen-sis*** | ***P. Canalicu-lata*** | ***P. Maximus*** |
| --- | --- | --- | --- | --- | --- | --- | --- | --- | --- |
| **No of genes** | 36140 | 38195 | 189750 | 34862 | 78735 | 41573 | 28569 | 25470 | 28975 |
| **No of genes in orthogroups** | 34983 | 37081 | 146033 | 30508 | 72887 | 38298 | 26619 | 23404 | 26626 |
| **No of unassigned genes** | 1157 | 1114 | 43717 | 4354 | 5848 | 3275 | 1950 | 2066 | 2349 |
| **% genes in orthogroups** | 96.8 | 97.1 | 77.0 | 87.5 | 92.6 | 92.1 | 93.2 | 91.9 | 91.9 |
| **% unassigned genes** | 3.2 | 2.9 | 23.0 | 12.5 | 7.4 | 7.9 | 6.8 | 8.1 | 8.1 |
| **No of orthogroups containing species** | 16627 | 16261 | 28745 | 16764 | 21909 | 16460 | 15606 | 12308 | 15732 |
| **% orthogroups containing species** | 33.1 | 32.4 | 57.2 | 33.4 | 43.6 | 32.8 | 31.1 | 24.5 | 31.3 |
| **No of species-specific orthogroups** | 870 | 975 | 12823 | 823 | 4930 | 1917 | 293 | 1157 | 307 |
| **No of genes in species-specific orthogroups** | 3638 | 4242 | 101573 | 3421 | 20789 | 8761 | 941 | 6306 | 991 |
| **% genes in species-specific orthogroups** | 10.1 | 11.1 | 53.5 | 9.8 | 26.4 | 21.1 | 3.3 | 24.8 | 3.4 |

**Supplementary Table S8.** Non-bivalve proteomes selected to search for potential DMRT genes.

| **Scientific name** | **Accession** |
| --- | --- |
| *Anopheles gambiae* | GCF_000005575.2 |
| *Danio rerio* | GCF_000002035.6 |
| *Drosophila melanogaster* | GCF_000001215.4 |
| *Homo sapiens* | GCF_000001405.40 |
| *Mus musculus* | GCF_000001635.27 |
| *Xenopus laevis* | GCF_017654675.1 |
| *Anopheles gambiae* | GCF_000005575.2 |

**Supplementary table S9.** DMRT genes found for each bivalve species analyzed.

|  | **DMRT1L** | **DMRT2** | **DMRT3** | **DMRT4/5** |
| --- | --- | --- | --- | --- |
| ***Crassostrea gigas*** | XP_011441049.2 |  | XP 011427033.2 | NP_001295834.1 |
| ***Crassostrea virginica*** | XP_022333989.1 |  | XP_022317913.1 | XP_022319926.1 |
| ***Dreissena polymorpha*** |  |  | GHIW01027633.1 | KAH3699546.1, KAH3782801.1, KAH3696108.1 |
| ***Limnoperna fortunei*** | ENSLFOG00000002085.1 | ENSLFOG00000002788.1 | g3385.t1 | ENSLFOG00000002613.2 |
| ***Mercenaria mercenaria*** |  | XP_045156965.1 | XP_045157038.1 | XP_045157053.1, XP_045159713.1, XP_045157593.1 |
| ***Mytilus galloprovincialis*** | VDI03798.1 | VDI42071.1 | VDI32052.1 | VDI24477.1 |
| ***Mizuhopecten yessoensis*** | XP_021353714.1 | XP 021368788.1 | XP_021377273.1 | XP_021377274.1 |
| ***Pecten maximus*** | XP_033733655.1 | XP_033738864.1 | XP_033737545.1 | XP_033737544.1 |
