# Supplementary material for: A chromosome-level assembly supports genome-wide investigation of the DMRT gene family in the golden mussel (Limnoperna fortunei)
Source: Gigascience. 2023 Sep 30;12:giad072. doi: 10.1093/gigascience/giad072 (PMC10541798; doi:10.1093/gigascience/giad072)
Supplement: giad072_Response_to_Reviewer_Comments_Original_Submission [file giad072_response_to_reviewer_comments_original_submission.pdf]

Author's Response To Reviewer Comments

Dear Editor,
We are pleased to resubmit the revised manuscript titled "A chromosome-level assembly supports genome-wide investigation of the DMRT gene family in the golden mussel (Limnoperna fortunei)" by Ferreira et al. for further evaluation and potential publication in GigaScience.

We sincerely thank the reviewers for their valuable feedback, which greatly improved the quality and clarity of our research. We appreciate their dedication, expertise, and guidance throughout this process.

The reviewers have recognized a significant improvement in the genome assembly of Limnoperna fortunei compared to its original draft version. They also recognized its importance in understanding the mussel's biology, evolutionary relationships within bivalves, and developing control strategies for this invasive species. However, they identified areas where additional metrics, evidence, and methodological improvements were needed to better showcase the strengths of the new genome assembly.

To attend the reviewers requests we have included a section (“Overall genome characteristics”) with k-mer based estimates of genomic features (e.g. heterozygosity rate). The section “Gene family evolution” was removed due to the criticism of poor selection of species, which made it limited for assessing lineage specific gene family expansions/contractions. As suggested by one of the reviewers, we have included preliminary analysis of potential hemizygosity in the golden mussel genome in a new section (“Hemizygosity investigation”). We also made some minor grammatical corrections throughout to improve the manuscript and added a section listing all authors from the Tree of Life Programme, as suggested by Gigascience recommendations on Group Authorship. Lastly, we updated the Ethics/compliance statement to make clear that the samples were obtained following the Brazilian Biodiversity Law.

In the subsequent sections of this letter, we will systematically address each point raised by the reviewers, providing comprehensive responses and incorporating the requested improvements into the revised manuscript.
Thank you for the opportunity to revise and resubmit our manuscript. We are confident that the revised version represents a significant contribution to the field and now meets the high standards for publication set by GigaScience.
Sincerely,

Juliana A. Americo
On behalf of all authors

Reviewer 1

1. "may lead to incomplete or erroneous gene models." -> I think the authors may introduce here some metrics of the first draft assembly that suggest that this was, indeed, the case, for example by mentioning the relatively high rate of missing/fragmented gene models.

Answer: We thank the reviewer for his comments and suggestions. We have now updated the manuscript, and we present such metrics. The sentence presenting these metrics is the following: ”Limitations of this draft genome constrain its applications in resequencing and comparative genomic studies and may lead to incomplete or erroneous gene models, as indicated by the high number of missing (10%) and fragmented (7%) BUSCO genes reported in the original paper [23]”.

2. "Recent advances in sequencing technologies and bioinformatics algorithms" -> I would also add something like "improved library preparation protocols", thinking about what makes both Hi-C and Hifi reads so useful.

Answer: Agreed and added to the text, thanks. The updated version of the sentence is: “Recent advances in library preparation protocols, sequencing technologies and bioinformatics algorithms have made the development of high quality reference genomes scalable and affordable.”

3. I have no methodological concerns, as the procedures described in the paper follow well-established standardized methodologies that have been employed for multiple other invertebrate genome projects in the context of large joint sequencing initiatives. I noticed that RNA-seq has not been mentioned anywhere as a source of data to complement gene annotation. I think the authors should mention whether they have exploited pre-existing data generated for example within the frame of the previous genome sequencing project.

Answer: We added the information about the datasets used to support annotation in the section “Gene prediction” and in the (new) Supplementary Table S2. The updated manuscript reads:

“The Ensembl rapid annotation pipeline [33] was used to predict genes. The prediction was supported by homologous proteins and pre-existing golden mussel RNA-seq data, including RNA-seq data from the draft genome project and from the same specimen (xbLimFort5) sequenced for the chromosome-level genome assembly (Supplementary Table S2)”.

4. "with two datasets: metazoa_odb10 and mollusca_odb10." -> while this makes sense, the Mollusca dataset is notoriously "bad" for such assessments at the present stage due to the low number of species used for its generation and a somewhat taxonomically-biased species composition, which often leads to nonsensical underestimates of completeness even for wonderful genome assemblies. Please consider removing the completeness data generated vs the molluscan dataset altogether, reporting only metazoan BUSCOs.

Answer: We agree with the reviewer, we have now removed the comparison to the molluscan dataset.

5. Gene prediction: how do the authors interpret the discrepancy between the number of gene models obtained with this pipeline and those reported for the previous version of the genome assembly? To what extent does the different quality of the assembly (i.e. lower fragmentation, much better chances to pick intact long genes, etc.) may explain this? To what extent does the annotation pipeline make a difference? I may also suggest to add (if useful) data about the mapping rate of RNA-seq datasets against the gene models of the two assemblies, as this would be another metric supporting a higher completeness of the present version of the assembly.

Answer: We have added the discussion about the difference in the number of genes and also compared the RNAseq mapping rate. The discussion now present in the manuscript is the following: “The number of predicted genes was significantly lower compared to the one reported for the draft genome (60 717) [23]. The draft nature of the previous genome could be a factor influencing prediction, because the genome was assembled in more than 20 000 scaffolds and the QV was low, and predictions can become fragmented and or truncated, overinflating the number of genes. This is in line with our new results, as even though the number of predicted genes for the chromosome-level assembly is lower (34 862), this prediction is much more complete as evidenced by a (i) 2.3-fold increase in the number of BUSCO genes found (Supplementary Table S4), (ii) decrease of duplicated and missing BUSCOs and a (iii) 1.8-fold increase in the number of RNAseq reads mapped (Supplementary Data Note 1 - Table 1).”

6. "10 000 000 MCMC generations" -> how was tree convergence evaluated?

Answer: Added to the manuscript: “Convergence was evaluated by checking if the standard deviation of split frequencies was <0.01”.

7. About the split gene of D. polymorpha, I would say that the interpretation provided by the authors is 100% correct. Rather than diverting the attention of the reader to this technical issue, I would simply suggest the authors to fish out the full length sequence from the transcriptome of the species (this should be available on TSA, if I remember correctly, without the need to de novo reassemble RNA-seq data) and replace the two partial gene models with the single correct full-length sequence. The whole process may be explained with a brief supplementary note.

Answer: Thanks for the suggestion. We have identified a transcript (GHIW01027633.1 from the GHIW00000000.1 transcriptome) that covers both genes and replaced the partial gene models (KAH3721156.1 and KAH3721157.1) by the complete one. The process is described in the Supplementary Data Note 3 and briefly mentioned in the main text: “Manual inspection to check for remaining isoforms and split gene models was also carried out (Supplementary Data Note 3).”

8. "raising the possibility that this domain may be absent in gastropods": yes, this is a possibility, but incorrect gene annotations are also quite frequent in bivalve genomes, so I would suggest the author to either name this alternative

possibility or to spend a few minutes at checking whether assembled transcripts from RNA-seq data support annotated gene models lacking this domain or not.

Answer: We have searched a Pomacea canaliculata transcriptome (datadryad.org/stash/dataset/doi:10.5061/dryad.8n776) but we were not able to find a complete transcript for the DMRT4/5 gene. Given the uncertainty related to the P. canaliculata DMRT4/5 gene model and the fact that gastropods were not the focus of our DMRT analysis, we decided to remove all P. canaliculata sequences from the phylogenetic analysis. The manuscript has also been adjusted to remove all references to those sequences.

9. "they are actually isoforms from the same gene that OrthoFinder's script to remove isoform duplicates was not able to detect." -> this is another technicality that diverts the attention of the reader. It's ok to mention this, but perhaps this would better fit in a supplementary note.

Answer: We have removed that sentence from the manuscript. In the new version of the DMRT tree we have deleted the shortest isoform, which was XP_022333988.1. Therefore, only XP_022333989.1 was kept. The removal is described in Supplementary Data Note 3.

10. The authors made an interesting remark about structural variation in the very last part of their manuscript, suggesting that this reference may be used to study whether gene PAV is present in L. fortunei. In perspective, such studies may be also useful to understand the genomic factors underlying the great invasive potential of this species, since dispensable genes are clearly enriched in functions linked with immunity and survival in Mytilus. I guess the assembly strategy used by the authors produced a primary reference assembly, plus an alternate haplotype, as it happens in many other recent genome assembly approaches that exploit Hi-C data (this is also briefly mentioned in the supplementary data file availability statement). While an in depth analysis of this data goes beyond the scope of the manuscript, I think the authors could report a few additional metrics in the section where they describe the main features of the assembly. For example, while I think high heterozygosity should be expected from this species, I have not seen any estimate reported in the text. Moreover, since heterozygosity rates estimates are usually performed with k-mer based graphs, these tend to mix-up SNP signals with hemizygosity (k-mers derived from both will end up in the same leftmost peak. The authors can easily estimate the fraction of the genome that is apparently in a hemizygous state simply by analyzing the per-base coverage calculated over relatively large window sizes. The distribution obtained with such calculations should (in case of widespread hemizygosity) also create two peaks, i.e. one corresponding to hemizygous regions not shared by the two haplotypes and one corresponding to core regions shared by both.

Answer: We have included a k-mer based estimate of the genome heterozygosity in a new section ("Overall genome characteristics"): "A k-mer based approach was used to estimate overall genome statistics from the PacBio HiFi data. Jellyfish [26] was used to calculate the frequencies of 31-bp long k-mers and GenomeScope [27] was used to build a model to infer genome characteristics. The genome was inferred to be diploid, with an estimated haploid size around 1.3 Gb (Supplementary Figure S1). The expected repeat content was 43% and a high heterozygosity rate was estimated (2.4%)."

We agree with the concern related to the hemizygosity and we have performed a preliminary identification of hemizygous regions. The analysis can be found in the new section: "Hemizygosity investigation". We have also updated other sections of the manuscript with information related to this new analysis. More specifically, we have added a sentence in the "Findings" topic of the "Abstract" section:
"A significant (6.48%) proportion of the genome was found to be in a hemizygous state". We have also added a paragraph in the Discussion section:
"Structural variation analysis showed that a significant (6.48%) proportion of the golden mussel genome was in a hemizygous state, i.e. the region is present in only one of the homologous chromosomes. The presence of hemizygous regions has already been seen in other molluscan species [44,55]. Compared to the eight molluscan species analyzed in Calcino et al [44], the golden mussel showed the second largest proportion of hemizygosity, only lower than the bivalve Scapharca (Anadara) broughtonii (6.69%). In another Mytilidae species (Mytilus galloprovincialis), it has been shown that the presence of hemizygous regions is correlated with the occurrence of gene presence/absence variation (PAV) [55], which, as discussed in the "Re-use potential" section, may have impacts on biotechnology-based control strategies. Future re-sequencing studies should allow us to move from a genome to a pangenome scenario and to check what set of golden mussel genes (if any) is under PAV."

11. Species choice: while I don't want to heavily criticize this, as I understand that this selection of species was enough

to meet the needs of the authors for the present, work, I would also say that this was somewhat limited for assessing lineage-specific gene family expansions and contractions, so the discussion should be a little bit more balanced, acknowledging the limitations of such inferences due to narrow taxonomical sampling. For example, M. philippinarum, P. viridis and B. platifrons, if added, would have provided a much better overview on possible lineage-specific expansions and contractions, intended as those that are not shared by other major mytiloid lineages. In its present form, the expansion/contraction analysis does not really adds much to the manuscript, as it fails to really identify expansion (or contraction) events that are restricted to the Limnoperna lineage, i.e. not shared by other mussels. On the other hand, another way to exploit this analysis would have been to check for convergent traits shared by Dreissena and Limnoperna, as they are both highly invasive and freshwater, despite the fact they belong to largely divergent branches of the bivalve tree of life. In summary, my suggestion would be to remove the CAFE analysis altogether, or alternatively to maintain just a brief mention of the potential usefulness of similar analyses that should however be carried out with a broader taxonomical sampling in order to be informative.

Answer: The CAFE analysis (previous section “Gene family evolution”) was removed from the manuscript as suggested by the reviewer.

12. Just brief comment on the phylogenetic analysis. The text is quite clear, as far as methodology is concerned, and the choice made by the authors are scientifically sound. Yet, I see a couple of minor issues that the authors should check, by looking at domain organization in Fig. 5. MSA is quite tricky whenever proteins displaying duplicated domains are present, as it is the case of some DMRTL1 sequences. The MSA was trimmed to remove noisy bits of sequence an unalignable stuff, yet some manual curation would have been needed in these specific cases and a few other obviously truncated sequences where the DM domain (the only portion shared by all sequence groups) was partial (or even entirely missing, in one case).

Answer: Thanks for the suggestion. We have done manual curation of the MSA after the automatic trimming and made sure we only kept regions that were conserved over sequences. We have also made sure that all domains (DM, Dmrt1, Dsx, DMA and DMB) were properly aligned. The final MSA is being submitted to GigaDB (DMRT_seqs_trimmed.fa) to be publicly available.

Reviewer 2

1. The authors should provide more detailed descriptions of the sequencing data and the assembly process.

Answer: We thank the reviewer for providing valuable input and suggestions in their review of the paper. Details on the assembly process may be found in section “Genome assembly”, and a file containing the commands ran for the assembly was submitted to the GigaDB public database (assembly.sh). As for sequencing data, the accession numbers of all genomic datasets are available under the section “Data availability”. Information on the RNA-seq data used to support gene prediction has been added in section “Gene prediction” and in the (new) Supplementary Table S2.

2. The authors should discuss the potential limitations in using the reference genome to control the invasion.

Answer: A discussion on those limitations has been added to the section “Re-use potential”. More specifically, in the following paragraphs:

“However, a reference genome based on the sequencing of a single specimen does not encompass the genetic diversity of the species. This limitation can compromise the development of efficient biotechnology-based control solutions that rely on specific target gene sequences. For example, Single Nucleotide Polymorphisms (SNPs) at the target site of a CRISPR-Cas9-based gene drive strategy can confer resistance to Cas9 cleavage, rendering the control strategy ineffective [53]. To identify potential critical SNPs and select invariant target sites, it will be necessary to resequence regions of interest in multiple individuals.
Structural variants have been detected in other mollusc species. In those species, a pattern of presence absence variation (PAV) has been reported, in which some genes are present only in some individuals of the population [44]. The chromosome-level genome may be used as a reference for future studies resequencing multiple golden mussel individuals to check whether the species is also under PAV and, if so, which parts of the genome are more or less conserved between individuals. Ultimately, only genes that are not subject to PAV should be considered potential targets for biotechnology-

based control strategies.”

3. The authors should discuss the implications of the findings in a broader context.

Answer: We have also aimed at improving that by adding some content in the “Re-use potential” section: “The chromosome-level genome can also be used as a reference for future population genomic studies. Understanding genomic variation among different golden mussel populations may unveil the routes of dispersion in invaded areas and support better control policies. Besides that, the new genome will support the study of chromosome evolution within Lophotrochozoan and Mytilidae through the comparison to chromosome-level genomes of related species. Lastly, the higher contiguity and accuracy of the new genome greatly benefits gene prediction quality aiming to more reliable studies of the evolution of genes and gene families.”

4. The results of Merqury showed that the QV score of 53 of the chromosome-level genome was 99.999%, indicating a high level of accuracy. It is also recommended to conduct analysis of genome completeness using Merqury to further assess the quality of the genome assembly. In addition, it is also suggested to add genome survey related analysis, as this can help obtain the size and heterozygosity of the genome, which are essential for guiding genome assembly and determining the redundancy of the assembly results.

Answer: Thanks for the suggestion. We have added a new section (“Overall genome characteristics”) containing genome statistics (e.g., heterozygosity rate) based on k-mer analysis of the PacBio HiFi reads:

“A k-mer based approach was used to estimate overall genome statistics from the PacBio HiFi data. Jellyfish [26] was used to calculate the frequencies of 31-bp long k-mers and GenomeScope [27] was used to build a model to infer genome characteristics. The genome was inferred to be diploid, with an estimated haploid size around 1.3 Gb (Supplementary Figure S1). The expected repeat content was 43% and a high heterozygosity rate was estimated (2.4%).”

We have also included more data (section “Genome assembly”) from the Merqury analysis, such as the requested completeness (Table 1) and also the Merqury plots (Supplementary Figure S2). The new sentence in the manuscript read:

“We used Merqury [34] with the PacBio HiFi reads and calculated an assembly-contained-kmer completeness of 99.23%, and a QV of 53, representing a base accuracy of 99.999% (Merqury plots can be found in Supplementary Figure S2)”

5. Please explain why authors chose to compare assembly metrics with Illumina-based genomes, while reference 23 reported a "hybrid-hierarchical genome assembly".

Answer: This is a confusion caused by the way we decided to call the first assembly: although the first version of the golden mussel genome (first assembly) was done with a hybrid strategy (Illumina plus low coverage of PacBio), most of the sequencing data used was Illumina, so we ended up naming it “Illumina-base”. However, we understand that it may be misleading and we have decided to change the way we name the different assemblies. In the revised version of the manuscript, the first assembly is now called “draft”, while the one reported in this study is referred to as “chromosome-level”.
