# Supplementary material for: A chromosome-level assembly supports genome-wide investigation of the DMRT gene family in the golden mussel (Limnoperna fortunei)
Source: Gigascience. 2023 Sep 30;12:giad072. doi: 10.1093/gigascience/giad072 (PMC10541798; doi:10.1093/gigascience/giad072)
Supplement: giad072_Reviewer_1_Report_Original_Submission — Marco Gerdol -- 2/12/2023 Reviewed [file giad072_reviewer_1_report_original_submission.pdf]

Reviewer Report

Title: A chromosome-level assembly supports genome-wide investigation of the DMRT gene family in the golden mussel (Limnoperna fortunei)

Version: Original Submission **Date:** 2/12/2023

Reviewer name: Marco Gerdol

Reviewer Comments to Author:

This manuscript reports the genome sequence of Limnoperna fortunei, a bivalve mollusk native to Asia but highly invasive in South America. The genome assembly now reaches a chromosome-scale, thereby representing a significant improvement compared with its original draft version, which was highly fragmented, hampering large-scale comparative analyses that the authors exemplified in this manuscript with a gene family contraction/expansion analysis. Moreover, the authors provide a hint about the possible practical implications of this novel resource, with the identification of a DMRT gene that may me targeted in the future as a measure of population control of this highly invasive species."may lead to incomplete or erroneous gene models." -> I think the authors may introduce here some metrics of the first draft assembly that suggest that this was, indeed, the case, for example by mentioning the relatively high rate of missing/fragmented gene models."Recent advances in sequencing technologies and bioinformatics algorithms" -> I would also add something like "improved library preparation protocols", thinking about what makes both Hi-C and Hifi reads so useful.I have no methodological concerns, as the procedures described in the paper follow well-established standardized methodologies that have been employed for multiple other invertebrate genome projects in the context of large joint sequencing initiatives.I noticed that RNA-seq has not been mentioned anywhere as a source of data to complement gene annotation. I think the authors should mention whether they have exploited pre-existing data generated for example within the frame of the previous genome sequencing project."with two datasets: metazoa_odb10 and mollusca_odb10." -> while this makes sense, the Mollusca dataset is notoriously "bad" for such assessments at the present stage due to the low number of species used for its generation and a somewhat taxonomically-biased species composition, which often leads to nonsensical underestimates of completeness even for wonderful genome assemblies. Please consider removing the completeness data generated vs the molluscan dataset altogether, reporting only metazoan BUSCOs.Gene prediction: how do the authors interpret the discrepancy between the number of gene models obtained with this pipeline and those reported for the previous version of the genome assembly? To what extent does the different quality of the assembly (i.e. lower fragmentation, much better chances to pick intact long genes, etc.) may explain this? To what extent does the annotation pipeline make a difference? I may also suggest to add (if useful) data about the mapping rate of RNA-seq datasets against the gene models of the two assemblies, as this would be another metric supporting a higher completeness of the present version of the assembly."10 000 000 MCMC generations" -> how was tree convergence evaluated?About the split gene of D. polymorpha, I would say that the interpretation provided by the authors is 100% correct. Rather than diverting the attention of the reader to this technical issue, I would simply suggest the authors to fish out the full

length sequence from the transcriptome of the species (this should be available on TSA, if I remember correctly, without the need to de novo reassemble RNA-seq data) and replace the two partial gene models with the single correct full-length sequence. The whole process may be explained with a brief supplementary note."raising the possibility that this domain may be absent in gastropods": yes, this is a possibility, but incorrect gene annotations are also quite frequent in bivalve genomes, so I would suggest the author to either name this alternative possibility or to spend a few minutes at checking whether assembled transcripts from RNA-seq data support annotated gene models lacking this domain or not."they are actually isoforms from the same gene that OrthoFinder's script to remove isoform duplicates was not able to detect." -> this is another technicality that diverts the attention of the reader. It's ok to mention this, but perhaps this would better fit in a supplementary note.The authors made an interesting remark about structural variation in the very last part of their manuscript, suggesting that this reference may be used to study whether gene PAV is present in L. fortunei. In perspective, such studies may be also useful to understand the genomic factors underlying the great invasive potential of this species, since dispensable genes are clearly enriched in functions linked with immunity and survival in Mytilus. I guess the assembly strategy used by the authors produced a primary reference assembly, plus an alternate haplotype, as it happens in many other recent genome assembly approaches that exploit Hi-C data (this is also briefly mentioned in the supplementary data file availability statement). While an in depth analysis of this data goes beyond the scope of the manuscript, I think the authors could report a few additional metrics in the section where they describe the main features of the assembly. For example, while I think high heterozygosity should be expected from this species, I have not seen any estimate reported in the text. Moreover, since heterozygosity rates estimates are usually performed with k-mer based graphs, these tend to mix-up SNP signals with hemizygosity (k-mers derived from both will end up in the same leftmost peak. The authors can easily estimate the fraction of the genome that is apparently in a hemizygous state simply by analyzing the per-base coverage calculated over relatively large window sizes. The distribution obtained with such calculations should (in case of widespread hemizygosity) also create two peaks, i.e. one corresponding to hemizygous regions not shared by the two haplotypes and one corresponding to core regions shared by both.Species choice: while I don't want to heavily criticize this, as I understand that this selection of species was enough to meet the needs of the authors for the present, work, I would also say that this was somewhat limited for assessing lineage-specific gene family expansions and contractions, so the discussion should be a little bit more balanced, acknowledging the limitations of such inferences due to narrow taxonomical sampling. For example, M. philippinarum, P. viridis and B. platifrons, if added, would have provided a much better overview on possible lineage-specific expansions and contractions, intended as those that are not shared by other major mytiloid lineages. In its present form, the expansion/contraction analysis does not really adds much to the manuscript, as it fails to really identify expansion (or contraction) events that are restricted to the Limnoperna lineage, i.e. not shared by other mussels. On the other hand, another way to exploit this analysis would have been to check for convergent traits shared by Dreissena and Limnoperna, as they are both highly invasive and freshwater, despite the fact they belong to largely divergent branches of the bivalve tree of life. In summary, my suggestion would be to remove the CAFE analysis altogether, or alternatively to maintain just a brief mention of the potential usefulness of similar analyses that should however be carried out with a broader taxonomical sampling in order to be informative.Just brief comment on the phylogenetic analysis. The text is quite clear, as far as

methodology is concerned, and the choice made by the authors are scientifically sound. Yet, I see a couple of minor issues that the authors should check, by looking at domain organization in Fig. 5. MSA is quite tricky whenever proteins displaying duplicated domains are present, as it is the case of some DMRTL1 sequences. The MSA was trimmed to remove noisy bits of sequence an unalignable stuff, yet some manual curation would have been needed in these specific cases and a few other obviously truncated sequences where the DM domain (the only portion shared by all sequence groups) was partial (or even entirely missing, in one case).

Level of Interest

Please indicate how interesting you found the manuscript: Choose an item.

Quality of Written English

Please indicate the quality of language in the manuscript: Choose an item.

Declaration of Competing Interests

Please complete a declaration of competing interests, considering the following questions:

- Have you in the past five years received reimbursements, fees, funding, or salary from an organisation that may in any way gain or lose financially from the publication of this manuscript, either now or in the future?
- Do you hold any stocks or shares in an organisation that may in any way gain or lose financially from the publication of this manuscript, either now or in the future?
- Do you hold or are you currently applying for any patents relating to the content of the manuscript?
- Have you received reimbursements, fees, funding, or salary from an organization that holds or has applied for patents relating to the content of the manuscript?
- Do you have any other financial competing interests?
- Do you have any non-financial competing interests in relation to this paper?

If you can answer no to all of the above, write 'I declare that I have no competing interests' below. If your reply is yes to any, please give details below.

I declare that I have no competing interests.

I agree to the open peer review policy of the journal. I understand that my name will be included on my report to the authors and, if the manuscript is accepted for publication, my named report including any attachments I upload will be posted on the website along with the authors' responses. I agree for my report to be made available under an Open Access Creative Commons CC-BY license (http://creativecommons.org/licenses/by/4.0/). I understand that any comments which I do not wish to

be included in my named report can be included as confidential comments to the editors, which will not be published.

Choose an item.

To further support our reviewers, we have joined with Publons, where you can gain additional credit to further highlight your hard work (see: https://publons.com/journal/530/gigascience). On publication of this paper, your review will be automatically added to Publons, you can then choose whether or not to claim your Publons credit. I understand this statement.

Yes Choose an item.
