# Supplementary material for: A chromosome-level assembly supports genome-wide investigation of the DMRT gene family in the golden mussel (Limnoperna fortunei)
Source: Gigascience. 2023 Sep 30;12:giad072. doi: 10.1093/gigascience/giad072 (PMC10541798; doi:10.1093/gigascience/giad072)
Supplement: giad072_Reviewer_2_Report_Original_Submission — Qingzhi Wang -- 2/14/2023 Reviewed [file giad072_reviewer_2_report_original_submission.pdf]

**Reviewer Report**

**Title: A chromosome-level assembly supports genome-wide investigation of the DMRT gene family in the golden mussel (Limnoperna fortunei)**

**Version: Original Submission** **Date:** 2/14/2023

**Reviewer name: Qingzhi Wang**

**Reviewer Comments to Author:**

The authors do a good job of identifying a knowledge gap and then address this need with multiple sequencing platforms and a series of overlapping techniques to analyze the sequences. This is an impressive work, of which provide the first chromosome-level reference genome for the golden mussel. High-quality of genome sequence information would be very useful in better understanding of biological function, their ecology and evolutionary relationships within bivalve species. The whole-genome assembly contains 99.4% of its total length and a scaffold N50 of 97.05 Mb, which should be valuable for understanding the biology of this species. The authors have done a great job in predicting 34 862 protein-coding genes, of which 84.7% were functionally annotated. The Orthofinder and CAFE analyses have revealed expanded gene families involved in structural and defense mechanisms. In addition, the manuscript contains a useful description providing a genomic landscape of DMRT gene family, and therefore this genome report will be a convenient resource for researchers using this genome sequence information as a target for population control strategies in other species.Overall, the authors have done a good job in providing a high-quality reference genome for the golden mussel and demonstrating its potential applications in biotechnology-based strategies. However, there are a few points that need to be addressed to improve the quality of this manuscript.I have several minor points for clarifications that may improve the manuscript (see below).1. The authors should provide more detailed descriptions of the sequencing data and the assembly process.2. The authors should discuss the potential limitations in using the reference genome to control the invasion.3. The authors should discuss the implications of the findings in a broader context.4. The results of Merqury showed that the QV score of 53 of the chromosome-level genome was 99.999%, indicating a high level of accuracy. It is also recommended to conduct analysis of genome completeness using Merqury to further assess the quality of the genome assembly. In addition, it is also suggested to add genome survey related analysis, as this can help obtain the size and heterozygosity of the genome, which are essential for guiding genome assembly and determining the redundancy of the assembly results.5. Please explain why authors chose to compare assembly metrics with Illumina-based genomes, while reference 23 reported a "hybrid-hierarchical genome assembly".

**Level of Interest**

Please indicate how interesting you found the manuscript: Choose an item.

**Quality of Written English**

Please indicate the quality of language in the manuscript: Choose an item.

**Declaration of Competing Interests**

Please complete a declaration of competing interests, considering the following questions:

- Have you in the past five years received reimbursements, fees, funding, or salary from an organisation that may in any way gain or lose financially from the publication of this manuscript, either now or in the future?
- Do you hold any stocks or shares in an organisation that may in any way gain or lose financially from the publication of this manuscript, either now or in the future?
- Do you hold or are you currently applying for any patents relating to the content of the manuscript?
- Have you received reimbursements, fees, funding, or salary from an organization that holds or has applied for patents relating to the content of the manuscript?
- Do you have any other financial competing interests?
- Do you have any non-financial competing interests in relation to this paper?

If you can answer no to all of the above, write 'I declare that I have no competing interests' below. If your reply is yes to any, please give details below.

I declare that I have no competing interests.

I agree to the open peer review policy of the journal. I understand that my name will be included on my report to the authors and, if the manuscript is accepted for publication, my named report including any attachments I upload will be posted on the website along with the authors' responses. I agree for my report to be made available under an Open Access Creative Commons CC-BY license (http://creativecommons.org/licenses/by/4.0/). I understand that any comments which I do not wish to be included in my named report can be included as confidential comments to the editors, which will not be published.

Choose an item.

To further support our reviewers, we have joined with Publons, where you can gain additional credit to further highlight your hard work (see: https://publons.com/journal/530/gigascience). On publication of this paper, your review will be automatically added to Publons, you can then choose whether or not to claim your Publons credit. I understand this statement.

Yes Choose an item.
